# Molecular basis for shifted receptor recognition by an encephalitic arbovirus

**DOI:** 10.1016/j.cell.2025.03.029

**Published:** 2025-04-04

**Authors:** Xiaoyi Fan, Wanyu Li, Jessica Oros, Jessica A. Plante, Brooke M. Mitchell, Jesse S. Plung, Himanish Basu, Sivapratha Nagappan-Chettiar, Joshua M. Boeckers, Laurentia V. Tjang, Colin J. Mann, Vesna Brusic, Tierra K. Buck, Haley Varnum, Pan Yang, Linzy M. Malcolm, So Yoen Choi, William M. de Souza, Isaac M. Chiu, Hisashi Umemori, Scott C. Weaver, Kenneth S. Plante, Jonathan Abraham

**Affiliations:** 1 Department of Microbiology, Blavatnik Institute, Harvard Medical School, Boston, MA, USA; 2 World Reference Center for Emerging Viruses and Arboviruses, University of Texas Medical Branch, Galveston, TX, USA; 3 Department of Microbiology and Immunology, University of Texas Medical Branch, Galveston, TX, USA; 4 Institute for Human Infections and Immunity, University of Texas Medical Branch, Galveston, TX, USA; 5 Department of Immunology, Blavatnik Institute, Harvard Medical School, Boston, MA, USA; 6 Department of Neurology, F.M. Kirby Neurobiology Center, Boston Children’s Hospital, Harvard Medical School, Boston, MA, USA; 7 Department of Microbiology, Immunology, and Molecular Genetics, University of Kentucky College of Medicine, KY, USA; 8 Department of Medicine, Division of Infectious Diseases, Brigham & Women’s Hospital, Boston, MA, USA; 9 Center for Integrated Solutions for Infectious Diseases, Broad Institute of Harvard and MIT, Cambridge, MA, USA; 10 Howard Hughes Medical Institute, Boston, MA, USA; 11 These authors contributed equally; 12 Lead contact

## Abstract

Western equine encephalitis virus (WEEV) is an arbovirus that historically caused large outbreaks of encephalitis throughout the Americas. WEEV binds PCDH10 as a receptor, and highly virulent ancestral WEEV strains also bind LDLR-related proteins. As WEEV declined as a human pathogen in North America over the past century, isolates have lost the ability to bind mammalian receptors while still recognizing avian receptors. To explain shifts in receptor dependencies and assess the risk of WEEV re-emergence, we determined cryo-electron microscopy structures of WEEV bound to human PCDH10, avian PCDH10, and human VLDLR. We show that one to three E2 glycoprotein substitutions are sufficient for a nonpathogenic strain to regain the ability to bind mammalian receptors. A soluble VLDLR fragment protects mice from lethal challenge by a virulent ancestral WEEV strain. Because WEEV recently re-emerged in South America after decades of inactivity, our findings have important implications for outbreak preparedness.

## In brief

Cryo-EM structures and mutational analyses reveal western equine encephalitis virus E2–E1 glycoprotein polymorphisms that determine shifted receptor usage and allow for sequence-based prediction of strain compatibility with human receptors.

## Graphical Abstract

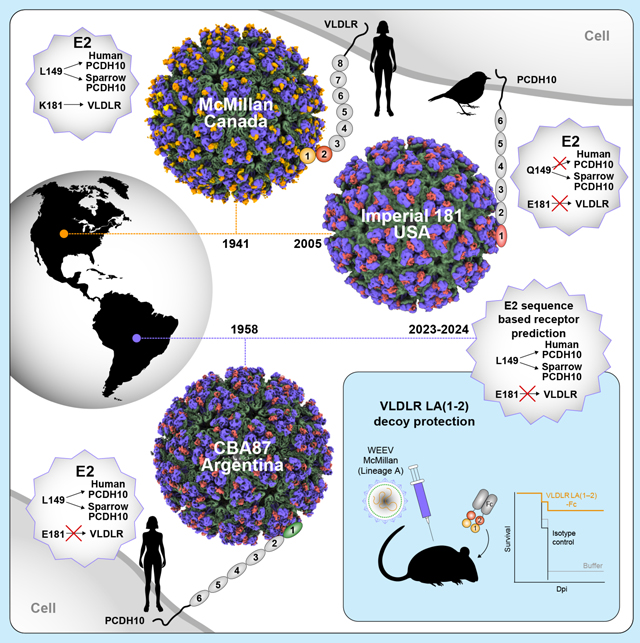

## INTRODUCTION

Several alphaviruses cause encephalitis outbreaks in humans and equids with unpredictable frequency and scale, including western equine encephalitis virus (*Alphavirus western*, WEEV), eastern equine encephalitis virus (EEEV), and Venezuelan equine encephalitis virus (VEEV).^1–3^ WEEV infection results in mild, asymptomatic illness or encephalitis, which can leave survivors with neurological sequalae.^3^ WEEV caused large outbreaks in the early 20^th^ century in North America, but outbreaks have since decreased in frequency and scale.^4^ The last documented human case of WEEV in North America was in 1999. Until recently, the last WEEV outbreak in South America was over three decades ago,^5^ with sporadic spillover events observed thereafter.^6^ However, WEEV re-emerged in South America in 2023, causing over 2,500 equine cases and over 200 human cases in three countries.^7–11^

**Figure 1.**
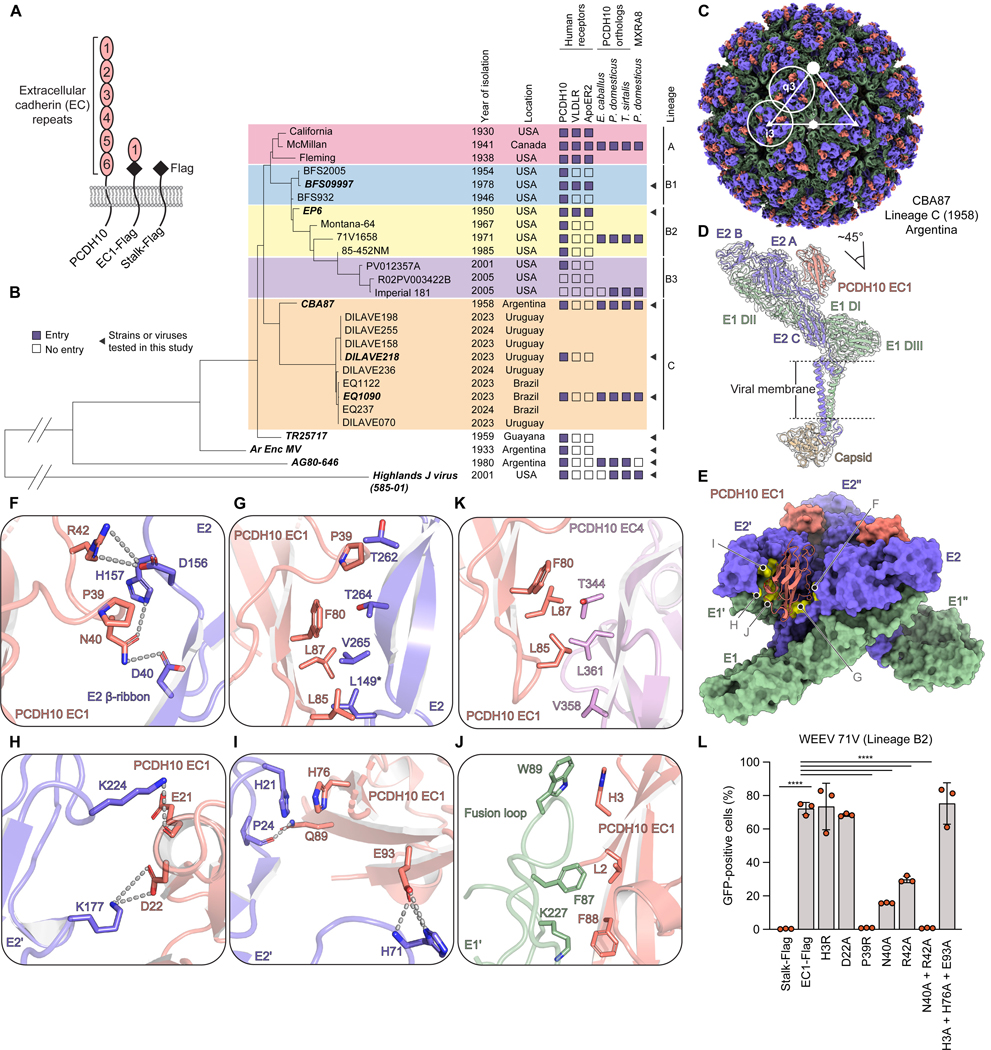
Structural basis for WEEV recognition of human PCDH10. (A) Schematic diagrams of PCDH10 and Flag-tagged constructs. (B) Partial phylogenetic tree and summary of K562 infectivity assays with GFP-expressing RVPs. Strains newly tested in this study are marked with black triangles; others are from Li et al.^15^ See also Figure S1. (C) Cryo-EM maps of *Hs*PCDH10_EC1_-Fc bound to WEEV CBA87 VLP with E2 in purple, E1 in green, and PCDH10 EC1 in pink. Icosahedral symmetry axes (i5, i3, i2) are indicated with a closed circle, triangle, and hexagon, respectively. The icosahedral i3 and q3 E2–E1 trimers are circled and indicated. (D) Ribbon diagram of a single WEEV E2–E1 heterodimer and PCDH10 EC1 fitted into its associated cryo-EM density map. The EC1 insertion angle relative to the E2–E1 trimer is shown. (E) CBA87 E2–E1 trimer bound by PCDH10 EC1 (surface rendered) with one EC1 as ribbons. WEEV E2–E1 residues that interact with EC1 are shown in yellow. Interaction details are in panels F to J. (F–J) PCDH10 EC1 interactions with WEEV E2–E1 (F and G) and E2’–E1’ protomers (H–J). Key polymorphic residue E2 L149 is marked with an asterisk. Hydrogen bonds and salt bridges are shown as dashed lines. (K) Interface between human PCDH10 EC1 and EC4 in a crystal structure of the PCDH10 EC1–4 homodimer (PDB: 6VFW).^19^ (L) Infection of K562 cells expressing WT (EC1-Flag) and mutant human PCDH10 EC1 constructs by GFP-expressing WEEV 71V RVPs at MOI=0.5, quantified by flow cytometry. Data are mean ± s.d. from three experiments performed in duplicates or triplicates (*n* = 3) (L). One-way ANOVA with Dunnett’s multiple comparisons test, ****P<0.0001 compared to stalk-Flag (L).

The alphavirus genome encodes four nonstructural proteins and structural proteins capsid, E3, E2, 6K, TF, and E1.^2^ The glycoproteins E2 and E1, which form heterodimers, assemble as 80 trimers on the virion surface that mediate binding to receptors and fusion with cellular membranes.^12–14^ WEEV E2–E1 binds protocadherin 10 (PCDH10), an adhesion molecule that is enriched in the brain and regulates synapse development.^15–18^ The PCDH10 ectodomain contains six extracellular cadherin repeats (EC1–6) connected by loops rigidified by calcium coordination (Figure 1A),^19^ and WEEV E2–E1 binds EC1.^15,16^ PCDH10 shares no homology with other alphavirus receptors (e.g., matrix remodeling-associated protein 8 (MXRA8) or low-density lipoprotein receptor (LDLR)-related proteins).^20–27^

WEEV strains isolated in North America are divided into lineages A and B; lineage B is subdivided into B1, B2, and B3 (Figures 1B, S1). Later lineages displaced earlier ones, and B3 is the most recently detected sublineage in North America.^4^ Strains from the most recent WEEV outbreak in South America are in a newly designated lineage C.^7^ While human PCDH10 is a receptor for most WEEV strains we previously tested,^15^ lineage A strains, which were isolated in the 1930s–1940s and are highly virulent in animal models, also bind very low-density lipoprotein receptor (VLDLR) and apolipoprotein E receptor 2 (ApoER2) (Figure 1B).^15,28^ Imperial 181, a B3 WEEV strain isolated from a mosquito pool in California in 2005, is nonpathogenic in animal models.^4,29^ Imperial 181 does not bind human or murine PCDH10 but binds the PCDH10 ortholog of house sparrows (*Passer domesticus*), which are an enzootic host for WEEV, and the ortholog of common garter snakes (*Thamnophis sirtalis*), which are proposed overwintering hosts.^15,30,31^ The basis for shifting patterns of receptor binding by different WEEV strains isolated in North America over the past century is unknown, nor are the receptor-binding properties of lineage C strains that recently re-emerged in South America.

Here, we conducted structural and functional analyses of WEEV interactions with human and avian receptors to define E2–E1 determinants of receptor binding, showing that only one to three E2–E1 substitutions explain the dramatic shifts in WEEV receptor recognition over the past century.

## RESULTS

### Structure of WEEV bound to human PCDH10

For structural analysis, we produced CBA87 virus-like particles (VLPs) (Figure S2A). This lineage C strain, isolated in Córdoba, Argentina, in 1958, has been used in investigational VLP-based vaccines modified for high-yield expression.^32^ In biolayer interferometry experiments, monomeric soluble EC1 interacted with immobilized CBA87 VLPs with a K_D_ of 5.6 μM (Figures S2A–E). This affinity is similar to that of the PCDH10 ectodomain for itself during homodimerization (K_D_ of 3.6 μM).^19^

We determined cryo-EM structures of CBA87 VLPs alone or bound to human PCDH10_EC1_-Fc (Figures 1C–E, S3A–G, and S4A). PCDH10 EC1 inserts into clefts formed by adjacent E2–E1 heterodimers and makes extensive contacts with E2 and E1 on both sides of each cleft, burying a large surface area (~1,500 Å^2^) (Figures 1D, 1E, and S3H). EC1 is in the same conformation when bound to WEEV E2–E1 or to EC4 in the PCDH10 homodimer (PDB: 6VFW)^19^ (Figure S3I).

One face of the cadherin repeat makes prominent contacts with the E2 β-ribbon connector (Figure S4D). These contacts involve PCDH10 residues N40 and R42 and WEEV residues D40, D156, and H157 (Figure 1F). Additionally, PCDH10 residues P39, F80, L85, and L87 contact E2 residues L149, T262, T264, and V265 (Figure 1G). The contralateral face of the cadherin repeat makes several contacts with the adjacent E2–E1 protomer (E2’–E1’) (Figure S4E). PCDH10 residues D22 and E21 make polar contacts with E2’ domain B residues K177 and K224 (Figure 1H). PCDH10 residue H76 and E2 residue H21 participate in stacking interactions (Figure 1I). PCDH10 residues L2 and H3 contact E1’ fusion loop residues F87 and W89, and PCDH10 residue F88 contacts E1’ residue K227 (Figure 1J). Other than the two fusion loop contact residues, WEEV E2–E1 residues that contact PCDH10 are not conserved in EEEV or VEEV, explaining why they do not bind PCDH10 (Figures S5A and S5B).^15^

Interestingly, the hydrophobic interactions WEEV E2 makes with PCDH10 EC1 are similar to how EC1 interacts with EC4 during PCDH10 antiparallel homodimerization as part of its physiological function.^19^ PCDH10 EC1 residues F80, L85, and L87 contact PCDH10 EC4 or WEEV E2 through similarly organized hydrophobic interactions (Figures 1G and 1K). Superposition of the EC1–EC4 homodimer (PDB: 6VFW)^19^ with the EC1-bound WEEV E2–E1 reveals steric clashes between one copy of EC1–EC4 and WEEV E2–E1 (Figure S3J). Furthermore, the EC1 surfaces contacted by WEEV E2–E1 or EC4 overlap (Figure S3K). Therefore, the EC1 surface that WEEV E2–E1 contacts is occluded in the PCDH10 homodimer, and only monomeric PCDH10 may facilitate WEEV E2–E1-mediated entry.

### Functional assessment of WEEV–PCDH10 interactions

Fourteen human PCDH10 EC1 residues contact CBA87 E2–E1 (Figure S5C). These residues are highly conserved among PCDH10 orthologs (human, murine, equine, avian, and reptilian) that serve as WEEV receptors (Figure S5C).^15^ To evaluate interactions between WEEV E2–E1 and human PCDH10, we performed infectivity assays on K562 cells, a lymphoblast-derived cell line that does not express PCDH10.^15,33^ We also use single-cycle reporter virus particles (RVPs) containing the Ross River virus genome with the E3–E2–6K/TF–E1 coding sequence replaced with GFP, and heterologous alphavirus glycoproteins on the virion surface.^34^ We transduced K562 cells with a truncated PCDH10 construct containing wild-type (WT) or mutated EC1 (Figure 1A). For infectivity assays, because our goal was to study the evolution of North American WEEV strains, we chose the lineage B2 WEEV strain 71V1658 (71V). Like CBA87, 71V binds PCDH10 but not VLDLR or ApoER2 (Figure 1B).^15^ Additionally, CBA87 E2–E1 residues that contact human PCDH10 are conserved in 71V (Figures S5A and S5B).

For most of the WEEV–PCDH10 interface, alanine substitution of individual or multiple PCDH10 residues that contact WEEV E2–E1 did not affect 71V RVP entry (Figures 1L and S6A–C), except for two EC1 residues, N40 and R42, that make polar contacts with E2 (Figure 1F). N40A and R42A individually reduced 71V RVP infection, but when combined, they abolished infection (Figure 1L). The EC1 P39R substitution, which would introduce steric clashes with nearby E2 residues, abolished 71V RVP entry (Figure 1L). Thus, in most cases, the large interaction interface between PCDH10 EC1 and WEEV E2–E1 tolerates substitutions that alter individual contacts with the receptor.

### WEEV interactions with avian PCDH10

**Figure 2.**
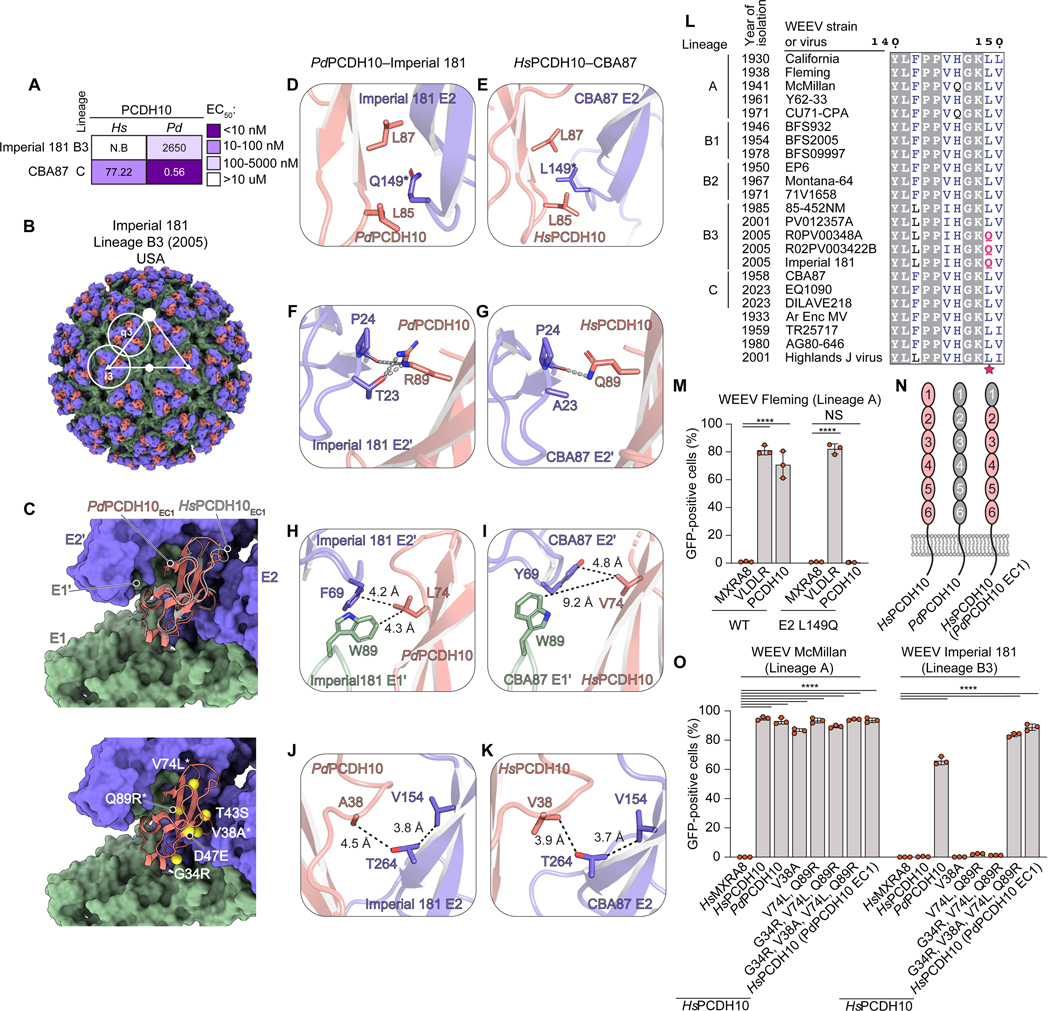
Structural basis for WEEV recognition of avian PCDH10. (A) EC_50_ values from ELISAs with *Hs*PCDH10_EC1_-Fc or *Pd*PCDH10_EC1_-Fc on immobilized WEEV VLPs. (B) Cryo-EM map of *Pd*PCDH10_EC1_-Fc bound to Imperial 181 VLP. E2 is in purple, E1 is in green, and PCDH10 EC1 is in pink. (C) *Top panel*: Top view of superposition of *Pd*PCDH10 EC1–Imperial 181 E2–E1 trimer and *Hs*PCDH10 EC1–CBA87 E2–E1 trimer. *Bottom panel*: Top view of *Pd*PCDH10 EC1–Imperial 181 E2–E1 trimer, highlighting six polymorphisms between *Hs* and *Pd*PCDH10 EC1. (D–K) Comparison of contact residues of *Pd*PCDH10-bound Imperial 181 (D, F, H, J) and *Hs*PCDH10-bound CBA87 (E, G, I, K), showing polar contacts (gray dashed lines) and the closest distances between PCDH10 residues and atoms on WEEV E2 (black dashed lines) (H–K). E2 residue L/Q149 is marked with an asterisk. (L) Partial WEEV E2 sequence alignment. E2 residue 149 is indicated with a star. Light gray background indicates completely conserved residues. Boxes indicate positions where a single majority residue or multiple chemically similar residues are present. E2 Q149 is colored pink. The panel was generated using ESPript 3.0.^58^ (M) K562 cells expressing *Hs*MXRA8, *Hs*VLDLR or *Hs*PCDH10 were infected with GFP-expressing WT or mutant Fleming RVPs at MOI=1. (N) Schematic diagrams of WT *Hs*PCDH10, *Pd*PCDH10, and a *Hs*PCDH10 chimeric construct with *Pd*PCDH10 EC1. (O) K562 cells expressing *Hs*MXRA8, *Hs*PCDH10, *Pd*PCDH10, *Hs*PCDH10 (*Pd*PCDH10 EC1), or *Hs*PCDH10 mutants were infected with GFP-expressing McMillan or Imperial 181 RVPs at MOI=3. Data are mean ± s.d. from two experiments performed in duplicates or triplicates (*n* = 3) (M, O). Two-way ANOVA with Dunnett’s multiple comparisons test, ****P<0.0001; ***P<0.001 (M, O); NS: not significant. See also Figure S6E.

Imperial 181 can bind sparrow PCDH10 but not human PCDH10 as a receptor.^15^ We used enzyme-linked immunosorbent assays (ELISAs) with VLPs and human (*Hs*) and sparrow (*Pd*) PCDH10_EC1_-Fc to compare half maximal effective concentration (EC_50_) values as a surrogate for affinity measurements. *Pd*PCDH10_EC1_-Fc, but not *Hs*PCDH10_EC1_-Fc, bound Imperial 181 VLPs. *Pd*PCDH10_EC1_-Fc and *Hs*PCDH10_EC1_-Fc bound CBA87 VLPs with different apparent affinities (1 nM and 77 nM, respectively). *Pd*PCDH10_EC1_-Fc bound Imperial 181 VLPs much less tightly (2.6 μM) than it bound CBA87 (1 nM) VLPs (Figures 2A and S2F).

To determine why Imperial 181 binds sparrow but not human PCDH10, we obtained the cryo-EM structure of Imperial 181 VLPs bound to *Pd*PCDH10_EC1_-Fc (Figures S4B and S7A–S7D). Human and sparrow PCDH10 bind WEEV with similar binding modes and contact residues (Figures 2C, S5A, S5B, and S7E). In the structure of CBA87 bound to human PCDH10, E2 L149 is in a cluster of hydrophobic residues that interacts with PCDH10 EC1. The analogous residue in Imperial 181, Q149, is polar but still positioned towards the same hydrophobic cluster (Figures 2D and 2E). Sparrow PCDH10 residue R89 makes polar contacts with E2 residues T23 and P24 (Figure 2F). In human PCDH10, R89 is replaced by glutamine (Q89) whose side chain only contacts the backbone carbonyl of E2 P24, providing weaker contributions to receptor interactions. The hydroxyl group of the T23 side chain in Imperial 181 E2 (A23 in CBA87) also contributes polar interactions (Figure 2G). Furthermore, the side chain of sparrow PCDH10 residue L74 is near a hydrophobic pocket involving E1’ fusion loop residue W89 (Figure 2H). In human PCDH10, this leucine is replaced by valine, whose smaller side chain does not contact E1’ W89 (Figure 2I).

E2 L149 is highly conserved in WEEV strains in lineage A, B1, B2, and is only replaced by a glutamine in lineage B3 strains recently isolated in the USA (2005), including Imperial 181 (Figures 2L and S8A). We previously tested B3 strain R02PV003422B and found that it also does not bind human PCDH10 (Figure 1B).^15^ The E2 L149Q substitution would likely disrupt key hydrophobic contacts with human PCDH10 EC1. Consistent with this notion, Fleming RVPs containing the E2 L149Q mutation could not infect cells expressing human PCDH10 (Figures 2M and S6D). The E2 L149Q polymorphism likely explains why some B3 strains do not bind human PCDH10.

### Multiple PCDH10 polymorphisms impact WEEV recognition

The ability of Imperial 181 to bind sparrow PCDH10 despite L149Q disrupting favorable hydrophobic contacts suggest that the sparrow ortholog makes compensatory contacts with E2–E1. Despite sequence differences in their EC repeats 2–6 and cytoplasmic tails, sparrow PCDH10 EC1 has only six substitutions compared to human PCDH10 EC1 (Figures 2C and S9). A chimeric PCDH10 construct where sparrow EC1 replaces human PCDH10 EC1 (*Hs*PCDH10(*Pd*PCDH10 EC1)) could support Imperial 181 RVP entry (Figures 2N, 2O, and S6E), suggesting that features in human EC1 alone explain why Imperial 181 does not bind human PCDH10.

Among these six polymorphic EC1 residues, only the V38A, V74L and Q89R substitutions involve residues that are close enough (<4 Å) to Imperial 181 E2–E1 to directly influence receptor binding (Figure 2C). The V74L and Q89R substitutions in human PCDH10 EC1 could in principle enable new hydrophobic and polar contacts with Imperial 181 E2, but they did not support Imperial 181 RVP entry when introduced into human EC1 (Figures 2F–I, 2O, and S6E). The G34R substitution involves a receptor residue that is not at the interface and, accordingly, had no effect on human PCDH10 recognition when added to V74L and Q89R (Figures 2O and S6E). The V38A substitution would remove unfavorable interactions between the valine side chain in human PCDH10 and E2 T264 (Figures 2J and 2K), but introducing the V38A substitution alone did not allow for Imperial 181 recognition (Figures 2O and S6E). When combined with G34R, V74L, and Q89R, the V38A substitution converted human PCDH10 into an efficient receptor for Imperial 181. McMillan was unaffected by any of the tested PCDH10 polymorphisms (Figure 2O). In sum, multiple favorable interactions formed by sparrow PCDH10 polymorphic residues absent in human PCDH10 are required for Imperial 181 recognition.

### Structural basis for VLDLR recognition

The VLDLR ligand-binding domain (LBD) contains eight cysteine-rich LA repeats (Figure S6F), each containing a Ca^2+^ ion coordinated by acidic residues next to an aromatic residue.^35^ These residues usually interact with basic residues on physiological ligands and viruses.^21,22,24–27,36–39^ Importantly, the critical basic residues in alphaviruses E2 or E1 glycoproteins that bind LA repeats are not conserved (Figures S5A and S5B).^22,24–27,40^

**Figure 3.**
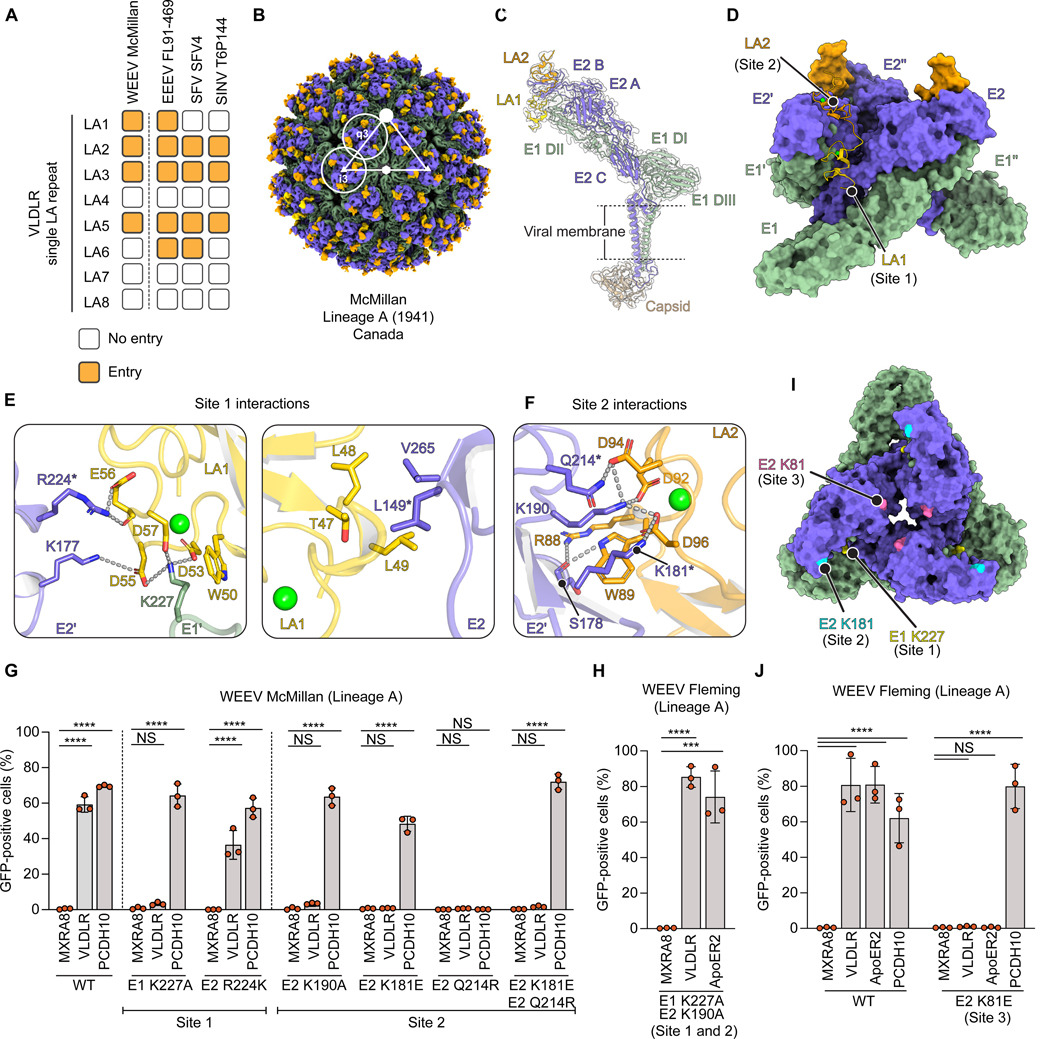
VLDLR recognition by ancestral WEEV strains. (A) Infectivity of GFP-expressing RVPs for the indicated alphaviruses with K562 cells expressing VLDLR single LA repeat constructs (see Figures S6F–H). EEEV, SFV, and SINV data are from a prior study.^24^ “Entry” indicates that the construct mediates statistically significant (*P* < 0.05) RVP infection when compared to LBD-lacking control. (B) Cryo-EM map of VLDLR_LBD_-Fc bound to McMillan VLP. E2 is purple, E1 is in green, VLDLR LA1 in is light yellow, and LA2 is in dark yellow. See also Figure S10. (C) Ribbon diagram of a single WEEV E2–E1 protomer and VLDLR LA(1–2) fitted into the cryo-EM density map. E2–E1 domains are indicated. (D) McMillan E2–E1 trimer bound to VLDLR (surface rendered) with one VLDLR LA(1–2) protomer rendered as ribbons. (E and F) McMillan contact with VLDLR LA1 (E) or LA2 (F) showing polar contacts (dashed lines), Ca^2+^ ions (green spheres), and polymorphic interacting residues (asterisks). (G) K562 cells expressing human MXRA8, VLDLR, or PCDH10 were infected by GFP-expressing WT or mutant WEEV McMillan RVPs at MOI=0.5. (H) K562 cells expressing human MXRA8, VLDLR, or ApoER2 were infected with GFP-expressing E1 K227A (site 1) + E2 K190A (site 2) mutant Fleming RVPs at MOI=1. (I) Top view of the WEEV E2–E1 trimer showing three potential binding sites for VLDLR LA repeats. Key residues are: E1 K227 (site 1), E2 K181 (site 2), and E2 K81 (site 3). (J) K562 cells expressing human MXRA8, VLDLR, ApoER2, or PCDH10 were infected with GFP-expressing WT or E2 K81E (site 3) mutant Fleming RVPs at MOI=1. Data are mean ± s.d. from three experiments performed in triplicates (*n* = 3) (G, H, J). One-way ANOVA with Dunnett’s multiple comparison test (H). Two-way ANOVA with Dunnett’s multiple comparisons test (G and J). ****P<0.0001 (G, H, J). MXRA8 vs. ApoER2 ***P = 0.0001 (H); NS: not significant.

We mapped LA repeat dependencies of WEEV McMillan, isolated from a human individual in Canada in 1941, using K562 cells stably expressing VLDLR truncation constructs with single LA repeats replacing the LBD (Figures S6F–H). McMillan could infect cells expressing LA1, LA2, LA3, and LA5 (Figures 3A and S6G), showing distinct LA repeat preferences compared to other alphaviruses (EEEV, Semliki Forest virus (SFV) and Sindbis virus (SINV)).^24–26,40^

We determined the cryo-EM structure of WEEV McMillan VLP bound to VLDLR_LBD_-Fc (containing eight LA repeats) (Figures S10A–C). In the receptor-bound E2–E1 trimer, two LA repeats bind clefts and contact adjacent E2–E1 protomers (Figures 3B–D). High-resolution maps allowed us to unambiguously build LA1 and LA2, with LA1 positioned deepest in the cleft (site 1), consistent with McMillan RVPs infection of K562 cells expressing VLDLR LA1 or LA2 (Figures 3A and S10D).

During interactions with VLDLR LA repeats, LA1 and LA2 bury 746 Å^2^ in each cleft of the McMillan E2–E1 trimer (Figure S3H). The LA repeat Ca^2+^-coordinating acidic residues usually make critical contacts with one or two basic residues on ligands, while the adjacent LA repeat aromatic residue stacks against the aliphatic portion of the lysine or arginine side chain.^36,37^ In site 1 of the VLDLR-bound WEEV complex, the side chains of LA1 acidic residues encircle WEEV E1ʹ residue K227, and the aliphatic portion of K227 stacks against LA1 W50. Acidic residues in LA1 also contact two E2ʹ domain B residues, K177 and R224 (Figure S4F). LA1 T47, L48, and L49 make nonpolar contacts with E2 β-ribbon residues L149 and V265 (Figures 3E). In site 2, the side chains of LA2 acidic residues interact with K190 on E2’ domain B, with the aliphatic portion of the K190 side chain stacking against LA2 W89 (Figure 3F). Additional polar contacts that involve the side chain or main chain atoms of WEEV E2ʹ residues K181, Q214 and S178, and LA2 residues D94 and R88, further anchor the LA repeat into place.

To assess contact residues, we infected K562 cells expressing receptors with WT or mutant McMillan RVPs. E1 residue K227 (site 1) and E2 residue K190 (site 2) are universally conserved in WEEV strains (Figures S8B, S11 and S12). McMillan RVPs containing either the E1 K227A or E2 K190A mutations were unable to infect K562 cells expressing VLDLR, despite retaining the ability to infect cells expressing human PCDH10 (Figures 3G and S6D). These observations suggest that simultaneous LA repeat engagement of sites 1 and 2 on McMillan E2–E1 is required for efficient VLDLR binding.

We next tested whether naturally occurring polymorphisms on the E2–E1 glycoproteins in site 1 (L149Q or R224K) or site 2 (K181E or Q214R) explain why previously examined lineage B strains do not bind VLDLR or ApoER2 (Figure S8B).^15^ The site 2 K181E or Q214R substitutions when individually introduced into McMillan RVPs abolished infection of K562 cells expressing VLDLR, suggesting that K181 and Q214 are required for VLDLR binding by McMillan. The E2 Q214R substitution, in addition to impairing VLDLR binding, unexpectedly prevented McMillan RVP infection of cells expressing PCDH10, despite E2 residue 214 not being near the PCDH10 binding site (Figure 3G). Because R214 can make a salt bridge with E181 (Figure S10E), we hypothesized that the E2 Q214R substitution destabilizes E2 domain B by closely positioning three basic residues (R214, K181, K190). McMillan RVPs containing both site 2 substitutions (K181E and Q214R), allowing R214 to potentially make the salt bridge with E181, could infect K562 cells expressing PCDH10. The site 1 R224K substitution when introduced into McMillan RVPs did not prevent infection of K562 cells expressing VLDLR, suggesting that lysine and arginine are interchangeable at this position (Figure 3G).

### Fleming uses a distinct VLDLR binding site

Most lineage A WEEV sequences contain K181 and Q214 in E2 (Figure S8B), suggesting they use the same VLDLR binding mode as McMillan. Fleming has E2 E181 (site 2), which impairs McMillan binding to VLDLR based on our mutational analysis, yet Fleming can still bind VLDLR (Figures 1B and 3G).^15^ Alanine substitution of E1 K227 (site 1) and E2 K190 (site 2) in Fleming RVPs had no impact on VLDLR-dependent entry (Figure 3H), suggesting that Fleming binds to VLDLR through a distinct binding mode. We examined the sequence of Fleming E2 and E1 proteins to identify potential binding sites involving unique, surface exposed lysine residues and identified E2 residue K81 (site 3), which is positioned near the threefold axis of the Fleming E2–E1 trimer but replaced by glutamate in other lineage A strains (Figures 3I and S8B). Interestingly, E2 K81 is adjacent to another basic residue, K82, that could also be recruited to participate in LA repeat binding by Fleming (Figure S11). The E2 K81E substitution abrogated Fleming RVP infection of K562 cells expressing VLDLR or ApoER2, while preserving PCDH10-dependent entry (Figures 3J and S6D). We propose the key determinant of LA repeat binding for WEEV Fleming is near the threefold axis of the trimer in a third potential LA repeat binding site.

### E2 substitutions reinstate receptor binding and neurotropism

PCDH10 and LDLR-related proteins are expressed on brain cells. Imperial 181 does not bind VLDLR, ApoER2, or PCDH10, and has been shown to replicate poorly in the brain of infected mice,^4,29,41^ suggesting an impaired ability to infect neurons and cause encephalitis compared to virulent WEEV strains.

**Figure 4.**
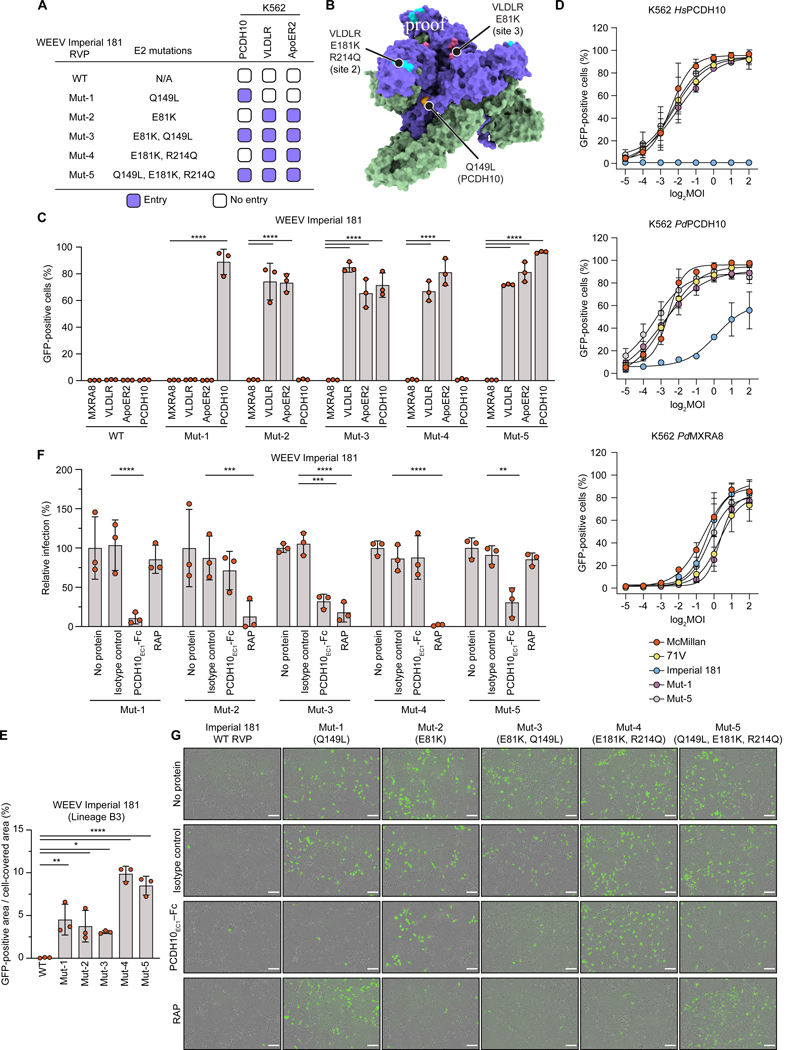
WEEV E2 protein polymorphisms affecting receptor recognition and neurotropism. (A) Imperial 181 mutants generated and summary of K562 infectivity assay in (C). (B) Side view of the WEEV E2–E1 trimer highlighting mutated E2 residues. (C) K562 cells expressing MXRA8, VLDLR, ApoER2, or PCDH10 were infected with WT or mutant Imperial 181 RVPs at MOI=1. (D) K562 cells expressing *Hs*PCDH10, *Pd*PCDH10, or *Pd*MXRA8 were infected with the indicated GFP-expressing RVPs at various MOIs. (E and F) Primary murine cortical neurons were infected with GFP-expressing WT or mutant Imperial 181 RVPs at MOI=2 in the absence of additional proteins (E and F) or in the presence of 316 μg ml^−1^ *Hs*PCDH10_EC1_-Fc, 100 μg ml^−1^ RAP, or 316 μg ml^−1^ isotype control (F). Absolute infection levels in the absence of additional proteins are shown in (E) and relative infection levels in the presence of indicated proteins normalized to infection levels in the absence of additional proteins are shown in (F). Infection was monitored through a live cell imaging system. (G) Representative merged images of GFP and bright field from (E and F). Scale bars: 100 μm. Data are mean ± s.d. from three experiments performed in duplicates or triplicates (*n* = 3) (C, D, E, and F). One-way ANOVA with Dunnett’s multiple comparisons test (E). Two-way ANOVA with Dunnett’s multiple comparisons test (C, and F). ****P < 0.0001 (C, E and F). WT vs. Mut-1 **P = 0.0028; WT vs. Mut-2 *P < 0.0111; WT vs. Mut-3 *P = 0.038 (E). Mut-2 isotype control vs. RAP ***P = 0.0004; Mut-3 isotype control vs. PCDH10_EC1_-Fc ***P = 0.0005; Mut-5 isotype control vs. PCDH10_EC1_-Fc **P = 0.0045 (F).

To test whether the lack of PCDH10, VLDLR, or ApoER2 binding explains why Imperial 181 poorly infects neurons, we generated five Imperial 181 RVP mutants containing E2 substitutions at polymorphic sites that should restore binding to the receptors. Mutant 1 (Mut-1) RVPs contain the E2 Q149L substitution that should restore human PCDH10 binding. Mut-2 RVPs contain the E2 E81K substitution that should restore LA repeat binding at site 3. Mut-3 RVPs contain the Q149L and E81K (site 3) substitutions. Mut-4 RVPs contain the E181K+R214Q substitutions that should restore LA repeat binding at sites 1 and 2. Mut-5 RVPs contain the Q149L and R214Q+E181K (sites 1+2) substitutions. K562 infectivity assays with cells overexpressing human PCDH10, VLDLR, and ApoER2 confirmed restoration of the expected receptor-binding properties for these mutant RVPs (Figures 4A–4C).

We assessed the relative efficiency of PCDH10 recognition by mutant Imperial 181 RVPs containing the Q149L substitution. We infected K562 cells expressing human or sparrow PCDH10 with Imperial 181 RVPs at different multiplicities of infection (MOI), with RVP titers determined on Vero E6 cells, which can be infected by Imperial 181 RVPs independently of PCDH10 or LDLR-related proteins.^15^ Though Imperial 181 RVPs recognized sparrow PCDH10 to infect K562 cells, they required a much higher MOI than McMillan or 71V RVPs to reach 50% infection (Figure 4D), suggesting that Imperial 181 uses sparrow PCDH10 less efficiently as a receptor. Interestingly, Mut-1 and Mut-5, containing the E2 Q149L substitution, not only gained the ability to infect cells expressing human PCDH10, but more efficiently infected cells expressing sparrow PCDH10 (Figure 4D). This observation suggests the E2 L149Q substitution decreases affinity for both human and sparrow PCDH10 as it removes favorable hydrophobic contacts. All tested RVPs could infect K562 cells expressing sparrow MXRA8 with similar efficiencies, suggesting the E2 L149Q polymorphism does not affect binding to sparrow MXRA8, despite also being at the avian MXRA8–E2 interface (Figures 4D and S5A).

We next used the five mutant RVPs to infect primary embryonic murine cortical neurons. WT Imperial 181 RVPs showed no infection, while the five mutants successfully infected these neurons. Treatment with *Hs*PCDH10_EC1_-Fc blocked infection by all mutants with restored mammalian PCDH10 recognition (Mut-1, Mut-3, and Mut-5). Treatment with the near-universal LDLR family ligand antagonist receptor-associated protein (RAP), which blocks alphavirus binding to VLDLR and ApoER2,^34^ blocked entry of mutants with restored VLDLR/ApoER2 but not PCDH10 recognition (Mut-2 and Mut-4). The “Fleming-like” composite Mut-3 was likewise blocked by RAP, suggesting that despite containing L149 in E2, Mut-3 still depends on LDLR-family receptors to infect these neurons. However, the “McMillan-like” composite Mut-5 was not affected by RAP, suggesting that Mut-5, unlike Mut-3, primarily depends on PCDH10 to infect these neurons (Figures 4E–4G).

### Sequence-based prediction of receptor binding

**Figure 5.**
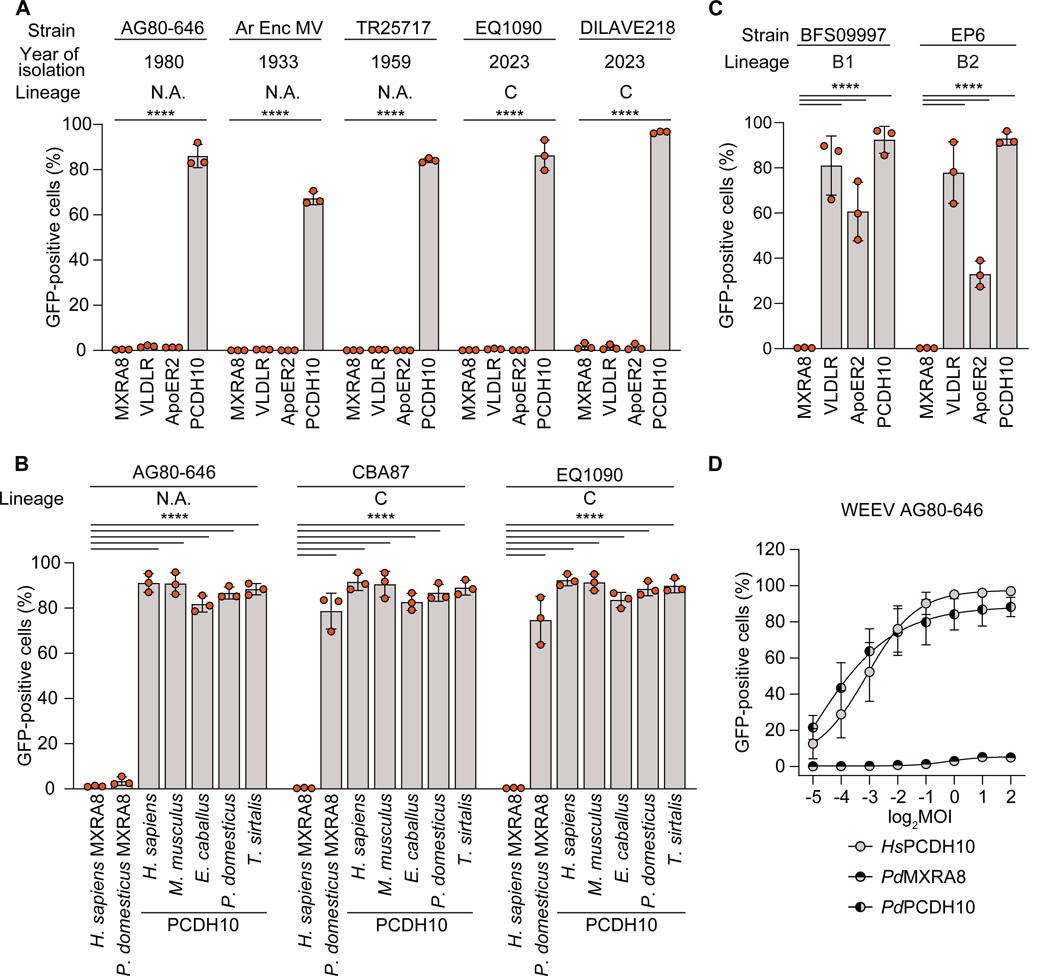
Prediction of WEEV strain receptor usage based on E2 glycoprotein sequences. (A) K562 cells expressing the indicated human receptors were infected with GFP-expressing RVPs for the indicated South American WEEV strains at MOI=0.5. (B) K562 cells expressing the indicated receptor orthologs were infected with GFP-expressing RVPs for the indicated South American WEEV strains at MOI=0.5. (C) K562 cells expressing the indicated human receptors were infected with the indicated GFP-expressing WEEV strain RVPs at MOI=0.5. (D) K562 cells expressing *Hs*PCDH10, *Pd*PCDH10, or *Pd*MXRA8 were infected with GFP-expressing AG80–646 RVPs at indicated MOIs. Data are mean ± s.d. from three experiments performed in duplicates or triplicates (*n* = 3) (A, B, C and D). Two-way ANOVA with Dunnett’s multiple comparisons test (A, B, C). ****P < 0.0001 (A, B, and C).

We next used knowledge of the E2 polymorphisms that influence WEEV recognition of human PCDH10 or VLDLR/ApoER2 to predict receptor dependencies of strains not yet experimentally tested. We focused on South American WEEV strains, including three with no lineage assignment (AG80–646, Ar Enc MV, and TR25717) and two lineage C strains isolated from the 2023–2024 outbreak, EQ1090 (Brazil, 2023) and DILAVE218 (Uruguay, 2023). All these strains contain L149 in E2 that we predict would confer binding to human PCDH10 (Figure 2L). They all lack lysine residues at E2 positions 181 (site 2) or 81 (site 3) and thus should not bind VLDLR or ApoER2 (Figure S8B). We confirmed our predictions using K562 infectivity assays (Figure 5A). In addition, AG80–646, CBA87, and EQ1090 recognized vertebrate PCDH10 orthologs for cellular entry (Figure 5B).

We next examined VLDLR binding sites in North American WEEV strains that we had not previously tested. We found that two strains, BFS09997 (sublineage B1) and EP6 (sublineage B2), contain K181 in E2, which would restore LA repeat binding site 2 and may allow these strains to bind VLDLR and ApoER2 (Figure S8B). BFS09997 and EP6 RVPs indeed infected K562 cells expressing VLDLR and ApoER2 (Figures 5C). Interestingly, both strains have R214, which our mutagenesis studies suggested prevents VLDLR/ApoER2 binding by the McMillan E2–E1 glycoprotein (Figure 3G). These two lineage B strains can tolerate the combination of E2 K181 and R214, which could be related to compensatory changes elsewhere in their E2–E1 glycoproteins. Our data suggest that E2 K181 is a determinant of VLDLR and ApoER2 binding that can be acquired by strains outside of the ancestral A lineage.

### Shift in receptors of a South American WEEV strain

AG80–646 is an enzootic WEEV strain isolated from mosquitoes in Argentina in 1980^42^ and likely represents a lineage highly divergent from lineage C. While all WEEV strains examined to date can bind avian MXRA8,^15,43^ we found that AG80–646 RVPs could not recognize sparrow MXRA8 to infect K562 cells, despite the ability to recognize sparrow PCDH10 (Figure 5B). Infectivity studies with AG80–646 RVPs at a range of MOIs on K562 cells expressing sparrow MXRA8 suggest that this strain has no affinity for sparrow MXRA8 (Figure 5D). Like WEEV lineages in North America, South American WEEV lineages may have also undergone shifts in their receptor-binding properties as they diverged.

### A WEEV-related alphavirus binds PCDH10

**Figure 6.**
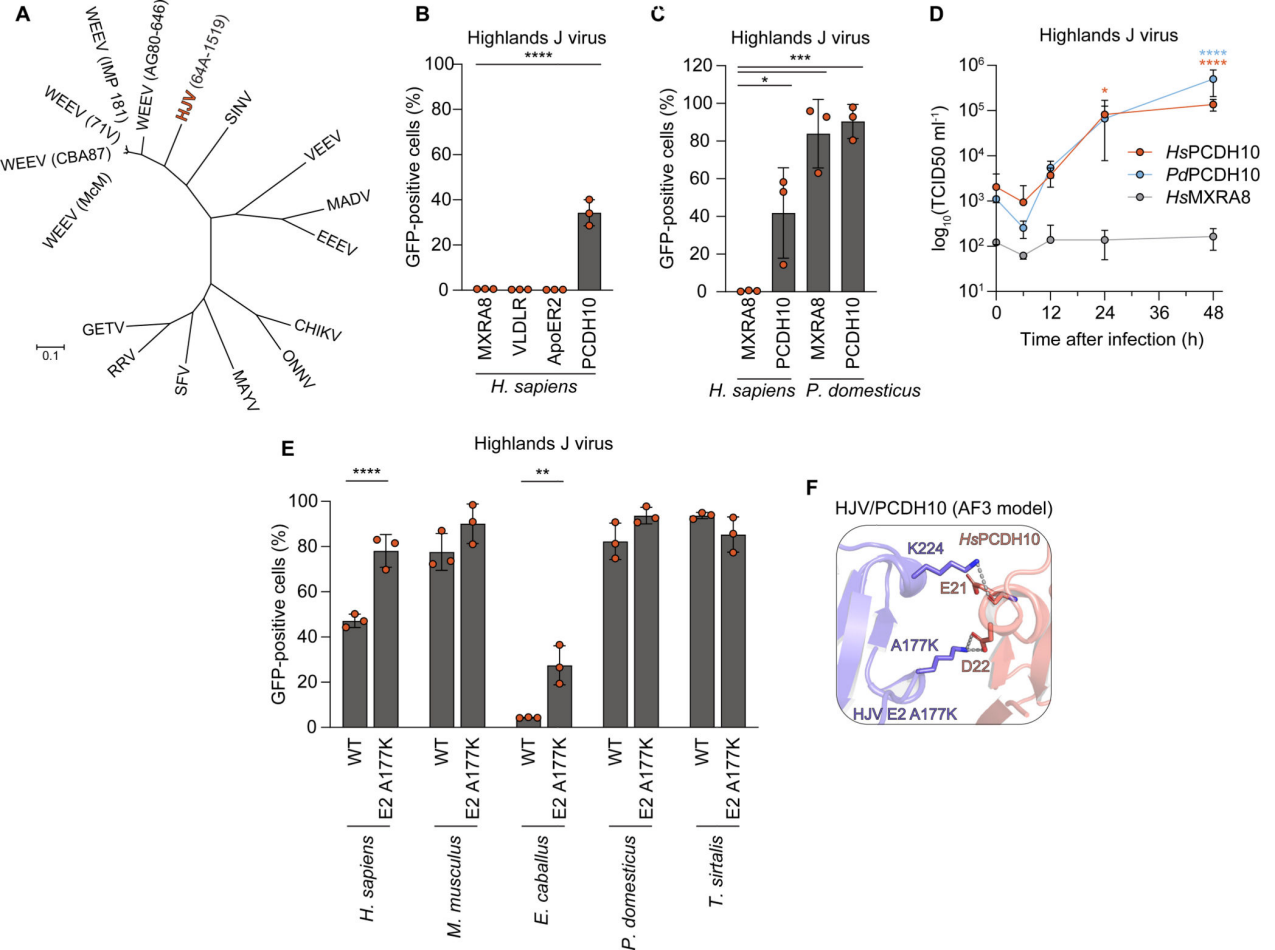
A WEEV-related North American alphavirus uses PCDH10 as a receptor. (A) Maximum likelihood phylogenetic tree of select alphaviruses based on the structural polyprotein coding sequences. WEEV and HJV strains are indicated in parentheses. IMP181: Imperial 181. McM: McMillan. See Table S1 for GenBank accession numbers. (B and C) K562 cells expressing indicated human or animal receptor orthologs were infected with GFP-expressing HJV 585–01 RVPs at MOI=1. (D) Viral replication for HJV (strain B 230) in transduced K562 cells at an MOI of 0.01. (E) Infection of K562 cells expressing PCDH10 orthologs with GFP-expressing WT and mutant HJV 585–01 RVPs at MOI=1. (F) AF3-predicted model^48^ of HJV 585–01 with an E2 A177K substitution bound by human PCDH10 EC1. See Figure S8C for predicted local distance difference test (pLDDT) scores. Data are mean ± s.d. from three experiments performed in duplicates or triplicates (*n* = 3) (B–E). One-way ANOVA with Dunnett’s multiple comparisons test (B and C). Two-way ANOVA with Šídák’s multiple comparisons test (D and E). ****P < 0.0001(B, D and E). *Hs*MXRA8 vs. *Hs*PCDH10 *P = 0.0299; *Hs*MXRA8 vs. *Pd*MXRA8 ***P = 0.0005; *Hs*MXRA8 vs. *Pd*PCDH10 ***P = 0.0003 (C). 24 h *Hs*MXRA8 vs. *Hs*PCDH10 *P = 0.0144 (D). *Ec*PCDH10 WT vs. E2 A177K **P = 0.0015 (E).

Highlands J virus (HJV) is a North American alphavirus closely related to WEEV that circulates on the East Coast of the USA (Figures 6A, and S1). HJV has an avian reservoir and is pathogenic in certain avian species.^44,45^ Although cases of human HJV infections have been documented incidentally in individuals co-infected with St. Louis encephalitis virus,^46^ and HJV was associated with a case of fatal equine encephalitis,^47^ HJV is not generally considered to be an equine or human pathogen and has no known receptors.

PCDH10 contact residues are conserved in the HJV E2–E1 glycoproteins, including E2 L149 (Figure S8A). LA repeat contact residues, however, are not conserved (Figure S8B). Accordingly, HJV RVPs recognized PCDH10 orthologs but not VLDLR or ApoER2 to infect K562 cells (Figures 6B and 6E). Interestingly, avian MXRA8 could serve as a receptor for HJV RVPs, consistent with a close evolutionary relationship between HJV and WEEV (Figure 6C). We also found that replication-competent HJV (strain B 230) replicated faster and to higher levels in K562 cells expressing both human and sparrow PCDH10, but not in cells expressing human MXRA8 (Figure 6D).

The HJV strain used in RVP production (585–01) could not efficiently enter cells expressing equine PCDH10 (Figure 6E). Sequence alignments and the PCDH10-bound WEEV structure revealed that a K177A substitution in HJV E2 may remove a salt bridge with PCDH10 D22 (Figure S8A). An interaction between an HJV E2–E1 trimer with E2 containing a lysine in position 177 (A177K) and human PCDH10 could be predicted using AlphaFold 3^48^ (Figures 6F and S8C). Supporting our predictions, HJV RVPs containing the A177K E2 substitution could infect K562 cells expressing equine PCDH10 (Figure 6E). Thus, the structures in combination with AlphaFold 3 modeling can clarify E2–E1 polymorphisms that modulate alphavirus receptor binding properties.

### E2 substitutions impact recognition of alternate receptors

**Figure 7.**
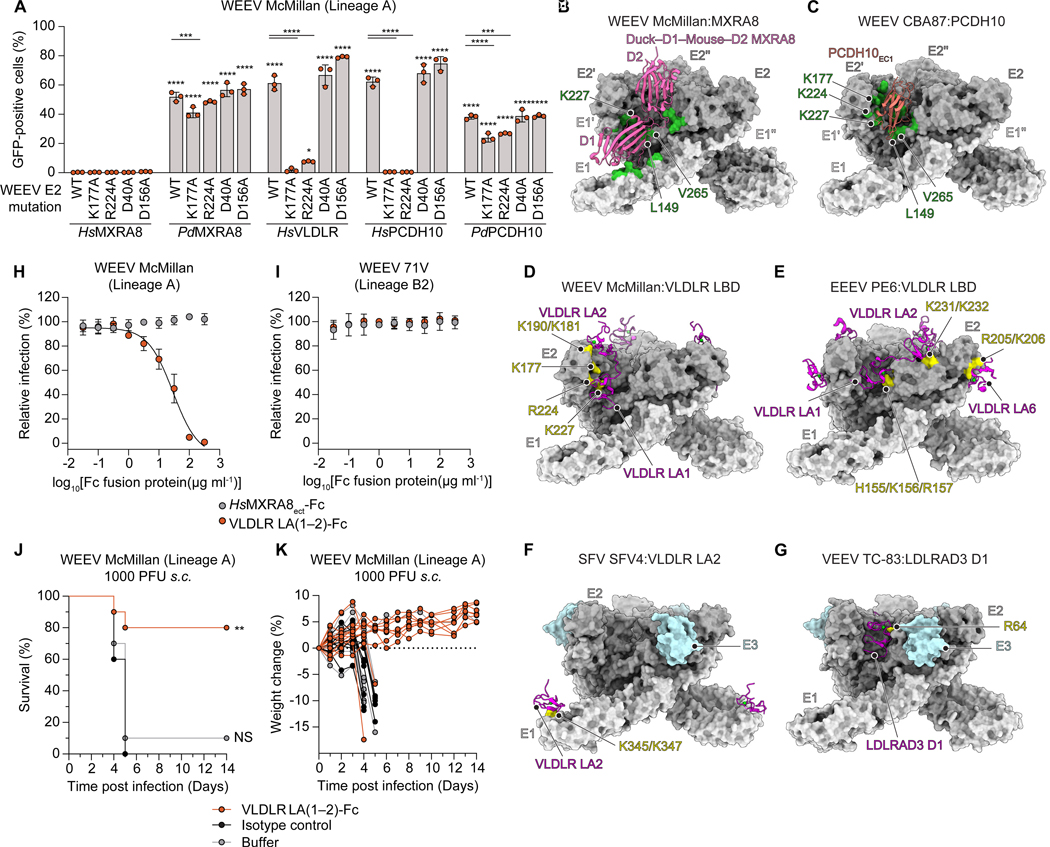
Comparison of alphavirus interactions with receptors and protective effect of a VLDLR LA(1–2) decoy protein. (A) K562 cells expressing indicated human or animal receptor orthologs were infected with GFP-expressing WT or mutant McMillan RVPs at MOI=1. Datasets are compared within groups (shown at the top) and with *Hs*MXRA8 (above each bar graph). (B) McMillan E2–E1 trimer (surface rendered) bound by a chimeric Duck–D1–Mouse–D2 MXRA8 (ribbons) (PDB ID: 8SQN).^43^ MXRA8 contact residues on E2–E1 are in green. Shared contact residues between duck MXRA8 and PCDH10 (E2 L149, E2 V265, and E1 K227) are indicated. (C) CBA87 E2–E1 trimer (surface rendered) bound by one human PCDH10 EC1 (ribbons). PCDH10 contact residues on E2–E1 are in green. Shared contact residues between PCDH10 and VLDLR (E2 K177, E2 K224, and E1 K227) or PCDH10 and duck MXRA8 (E2 L149, E2 V265 and E1 K227) are indicated. (D–G) Comparison of alphavirus E2–E1 trimers (surface rendered) bound by LA repeats (ribbon). The key basic residues on WEEV McMillan (D), EEEV PE6 (E) (PDB ID: 8UFB),^25^ VEEV TC-83 (G) (PDB ID: 7FFF)^22^, and SFV SFV4 (F) (PDB ID: 8UA8),^24^ are in yellow. LA repeats are in magenta; E2–E1 glycoproteins are in different shades of gray; E3 is in light blue. Calcium ions are in green. D1: domain 1. (H and I) McMillan (H) or 71V (I) RVPs were pre-incubated with the indicated Fc fusion proteins and used to infect K562 cells expressing human PCDH10. (J and K) Six-week-old CD1 mice were administered VLDLR LA(1–2)-Fc fusion protein, an isotype control antibody, or a buffer diluent intraperitoneally 6 h before subcutaneous inoculation with 1000 PFU of WEEV McMillan rescued from a molecular clone. Survival (J) and weight change (K) of the mice were monitored. Data are mean ± s.d. from three experiments performed in duplicates or triplicates (*n* = 3) (A, H, and I). ****P < 0.0001; *Pd*MXRA8 WT vs. K177A ***P = 0.0003; *Pd*PCDH10 ET vs. R224A ***P = 0.0001; R224A *Hs*MXRA8 vs. *Hs*VLDLR *P = 0.0178 (A). For VLDLR LA(1–2)-Fc protection experiment (J): buffer only, *n* = 10; VLDLR LA(1–2)-Fc, *n* = 10; isotype control, *n* = 10 mice. Log-rank (Mantel–Cox) test comparing VLDLR LA(1–2)-Fc or isotype control to buffer. **P<0.01; NS, not significant.

McMillan binds human and sparrow PCDH10, human VLDLR and ApoER2, and sparrow MXRA8 (Figure 1B).^15,43^ We examined whether McMillan E2 residues that contact one or more receptors affect recognition of other receptors. Alanine substitutions of E2 D40 and D156, which contact PCDH10 EC1 residues N40 and R42, individually had no effect on McMillan recognition of PCDH10 or VLDLR (Figures 1F and 7A). Alanine substitutions of E2 K177 and R224, which contact both PCDH10 and VLDLR LA1, ablated recognition of human but not sparrow PCDH10, and also decreased VLDLR-dependent entry (Figures 7A and S5A). All the E2 substitutions tested minimally impacted McMillan recognition of sparrow MXRA8, as none of them involve avian MXRA8 contact residues (Figures S5A and S5B).^43^ Our analysis suggests that WEEV E2 is more tolerant of individual substitutions when recognizing avian PCDH10 but less with human PCDH10 or VLDLR.

### Comparison with other alphavirus receptor-bound structures

The cleft between adjacent E2–E1 heterodimers serves as the major site of receptor engagement, as seen in WEEV with PCDH10 or VLDLR, and in prior structures of WEEV with avian MXRA8, VEEV with LDLRAD3, EEEV with VLDLR (Figures 7B–7F) and Chikungunya virus (CHIKV) with mammalian MXRA8.^20–26,43^ The exception is SFV, which uses a surface on E1 domain III outside the cleft (Figure 7G).^24,27,40,49^ Buried surface areas (BSA) vary significantly across these receptor-virus complexes. PCDH10 and MXRA8 have a larger BSA when interacting with WEEV and CHIKV E2–E1 glycoproteins compared to the individual LA repeats in VLDLR or LDLRAD3 in their interactions with WEEV McMillan, EEEV, SFV and VEEV (Figure S3H). Interestingly, while VEEV, EEEV, SFV, and WEEV all bind LA repeats, binding modes differ markedly. This variation arises from the distinct positioning of critical basic residues on E2–E1 that interact with calcium-coordinating acidic residues in the LA repeats (Figures 7D–G).

### A VLDLR LA1–LA2 receptor decoy protects against lethal WEEV challenge

Adams et al. recently reported that an Fc-fusion receptor decoy containing VLDLR LA1 and LA2 (VLDLR LA(1–2)-Fc) protects against lethal EEEV infection in mice when administered six hours prior to viral challenge.^25^ Our structures suggest that VLDLR LA(1–2)-Fc would block access to both VLDLR and PCDH10 and may also be active against McMillan. Indeed, VLDLR LA(1–2)-Fc neutralized McMillan RVP infection of K562 cells expressing PCDH10 with an EC_50_ of 1.4 μg ml^−1^ (Figures 7H and 7I). Eighty percent of CD1 mice that received a single, 25 mg kg^−1^ dose of VLDLR LA(1–2)-Fc six hours prior to subcutaneous inoculation with 1000 plaque-forming units of WEEV McMillan survived, whereas all of the mice that received an isotype control IgG succumbed to infection (Figures 7J and 7K). VLDLR LA(1–2)-Fc thus has broad activity against EEEV and a virulent WEEV strain that binds LDLR-related proteins.

## DISCUSSION

We report here structures of WEEV bound to two of its structurally unrelated receptors, PCDH10 and VLDLR. Extensive mutational analyses based on the structures allow us to define the polymorphisms in WEEV E2 that have driven shifts in the receptor-binding properties of strains isolated over the past century (Figure S8D).

Imperial 181 has a much lower apparent affinity to sparrow PCDH10 compared to CBA87 (Figure 2A), suggesting the involvement of additional receptors in facilitating Imperial 181 entry in sparrow hosts. Efficient interactions with avian MXRA8 seem to be maintained in WT or mutant viruses that contain E2 Q149, which can abrogate binding to human PCDH10 (Figures 4D and S7F). Examining a previous structure of duck MXRA8 bound to McMillan E2–E1 (PDB: 8DAN),^43^ we found that the E2 L149Q substitution could be easily accommodated at the interface (Figure S7G). Thus, MXRA8 may play a role as a higher affinity receptor during enzootic transmission of Imperial 181-like North American WEEV strains in avian hosts.

Our phylogenetic tree using the structural polyprotein coding sequences of 57 WEEV strains shows that E2 L149Q, which abolishes human PCDH10 binding, is a signature substitution of the most recently isolated North American B3 clade, to which Imperial 181 belongs (Figure S1). Our tree also shows that newer North American isolates tend to be placed in new clades instead of extending older clades, confirming previous findings of rapid clade displacement in circulating WEEV.^4,50^ If clade displacement has continued in the past two decades, the E2 Q149 clade may have displaced older clades, marking further epizootic decline of this pathogen. Indeed, strains in this clade were isolated in California and Texas (Table S1), suggestive of its geographic expansion. Further environmental sampling is required to test this hypothesis.

McMillan and California form a clade within lineage A that can be defined as containing a lysine at E2 position 181, which is a critical determinant of VLDLR and ApoER2 binding (Figures 3F and 3G). K181, however, is likely replaced by E181 in the common ancestor of lineage A, since Fleming contains glutamate at that position and lies basal to California and McMillan in the phylogenetic tree (Figure S1). The California/McMillan clade likely evolved from an ancestor containing E181 in the E2 protein that acquired a E181K substitution; this ancestor went on to spill over and resulted in epidemics in the 1930s–1940s. The California/McMillan clade would later become extinct and displaced. Identification of two lineage B strains that contain the E2 E181K substitution and can bind VLDLR and ApoER2 (BFS09997 and EP6) (Figure 5C) supports the notion that E2 E181K may be a transitory mutation that could arise stochastically. Of note, while McMillan and California were more extensively passaged, BFS09997 and EP6 were minimally passaged, suggesting that E2 E181K is a naturally occurring substitution as opposed to the result of passaging.

Our mutational analysis suggests that Fleming uses a different surface containing E2 K81 to bind VLDLR and ApoER2. A previous study proposed that the E2 E81K substitution in Fleming may be a result of cell culture adaptation during passaging.^51^ Mn548, a group B2 strain that also contains E2 K81, has an unknown source of isolation and passage history, making it difficult to assess the origin of the E2 K81 polymorphism (Figures S1, and S8B). Nonetheless, our finding suggests that E2 K81 polymorphism, which creates new LA repeat binding sites, is important to monitor during environmental surveillance. Using Imperial 181 mutant RVPs, we observed that Fleming’s distinct VLDLR binding mode (as reconstructed in Mut-3) results in stronger dependence on LDLR-related receptors during neuronal entry, reflected by the blocking of Mut-3 infection of murine neurons by RAP (Figures 4F and 4G). Infection by the McMillan-like mutant (Mut-5), however, was not blocked by RAP in these experiments. These findings suggest that different LA repeat binding modes in WEEV strains may influence relative dependencies on PCDH10 and LDLR-related receptors during entry.

Strains from the South American C lineage all contain leucine at E2 position 149, allowing them to bind human and equine PCDH10. While WEEV has submerged as a pathogen in North America, in South America, serological evidence suggests that WEEV continued to spill over into epizootic hosts well into the 21^st^ century.^52–54^ The ability of WEEV to persist in epizootic circulation in South America may explain the preservation of E2 L149, as well as mammalian PCDH10 recognition, by lineage C.

Two isoforms of PCDH10 are expressed from the *PCDH10* gene through alternative splicing, producing distinct cytoplasmic domains with identical ectodomains, including EC1.^55^ Because EC1 is sufficient as a binding site for WEEV and the cytoplasmic tail of PCDH10 is dispensable for entry,^15^ WEEV would not be expected to preferentially recognize any specific isoform. Interestingly, the EC1 residues of PCDH10 that interact with WEEV are not conserved in its two closest δ2 protocadherin family members, PCDH17 and PCDH19, suggesting WEEV cannot infect cells by binding to PCDH17 or PCDH19 (Figure S5D).^19^

We observed full occupancy of the 240 potential PCDH10 EC1 binding sites in cryo-EM maps for the full VLPs. However, superimposing the X-ray crystal structure of PCDH10 EC1–6 (PDB ID: 6VG4)^19^ with the structure of WEEV bound to EC1 revealed steric clash at EC2 if all three clefts of the WEEV E2–E1 trimers are bound by the full receptor ectodomain (Figure S3L). We suspect that each WEEV E2–E1 trimer may engage only one PCDH10 molecule. This is reminiscent of rhinovirus-C binding to its receptor cadherin related protein 3, in which EC1 also interacts with the virus, and EC2 sterically limits occupancy.^56^

A study by Mossel et al. examining the effects of WEEV E2 substitutions on pathogenicity in mice showed that E2 Q214R, which impairs McMillan binding to PCDH10, VLDLR, or ApoER2, drastically decreases McMillan’s mortality in infected mice. E2 K181E, which impairs binding to VLDLR and ApoER2 but not PCDH10, moderately decreased mortality while delaying the mean time to death by two days (Figures 3G and S8E).^41^ Thus, the ability to bind PCDH10, VLDLR, and ApoER2 likely influences WEEV pathogenicity in mice. Indeed, multiple recent studies support the notion that VLDLR promotes neuronal infection and lethal neuropathogenesis of McMillan.^15,28,57^ Although lineage A has gone extinct and currently known epizootic lineage B and C strains do not bind VLDLR, we found evidence that enzootic WEEV strains of any lineage may sporadically acquire VLDLR recognition. Should these WEEV variants with potentially increased neurovirulence re-emerge, our *in vivo* study suggests that VLDLR LA(1–2)-Fc could be a therapeutic option.

Overall, our structures could facilitate further studies examining the contributions of individual receptors to WEEV pathogenesis and set the stage for rational enhancement of receptor decoy potency. Our findings also provide a guide for estimating the potential threat any given WEEV strain might pose based on sequence polymorphisms in its E2–E1 glycoproteins as determinants of virus receptor dependencies and propensities for neurotropism, facilitating environmental surveillance and bolstering outbreak preparedness.

### Limitations of the study

We used ELISAs to estimate apparent affinities of PCDH10_EC1_-Fc fusion proteins with VLPs. Because of the dimeric nature of Fc-fusion proteins, these apparent affinities would be an overestimate of affinities due to avidity. Biolayer interferometry experiments with immobilized VLPs and monomeric EC1 constructs could be used for accurate assessment of affinities. We tested the prophylactic but not therapeutic efficacy of VLDLR LA(1–2)-Fc at a single dose, which resulted in 80% survival in mice treated with the decoy protein. It is possible that higher doses of treatment could offer full protection against lethal outcomes. The post-exposure protective efficacy of this decoy protein requires further investigation.

## RESOURCE AVAILABILITY

### Lead Contact

Further information and requests for resources and reagents should be directed to and will be fulfilled by the Lead Contact, Jonathan Abraham (jonathan_abraham@hms.harvard.edu).

### Materials Availability

Reagents generated in this study are available from the Lead Contact upon request with completed material transfer agreements.

### Data and Code Availability

- Protein Data Bank (PBD) and Electron Microscopy Data Bank (EMDB) identification numbers for the cryo-EM structures and maps reported in this manuscript are available as of the date of publication. Identification numbers are listed in the key resources table.
- This paper does not report original code.
- Any additional information required to reanalyze the data reported in this paper is available from the Lead Contact.

## STAR METHODS

### EXPERIMENTAL MODEL AND STUDY PARTICIPANT DETAILS

#### Cell lines

All cell lines used in this study are listed in the key resources table. HEK 293T (human kidney epithelial, ATCC CRL-11268), Vero E6 (*Cercopithecus aethiops* kidney epithelial, ATCC CRL-1586) were maintained in Dulbecco’s modified Eagle’s medium (DMEM, Gibco) supplemented with 10% (v/v) fetal bovine serum (FBS) and 25 mM HEPES (Thermo Fisher Scientific). K562 (human chronic myelogenous leukemia, ATCC CCL-243) cells were maintained in RPMI1640 (Thermo Fisher Scientific) supplemented with 10% (v/v) FBS, 25 mM HEPES, and 1% (v/v) penicillin-streptomycin. Expi293F cells (Thermo Fisher Scientific Cat#: A14527) were maintained in Expi293 Expression Medium (Thermo Fisher Scientific). Cell lines were not authenticated. Absence of mycoplasma was confirmed through monthly mycoplasma testing using e-Myco PCR detection kit (Bulldog Bio Cat#: 25234).

#### Viruses

Highlands J virus (strain B230) was obtained ATCC (Cat#: VR262). Western equine encephalitis virus (strain McMillan) was rescued from an infectious clone^60^.

### METHOD DETAILS

#### Rescue of WEEV McMillan and plaque assays

Ten micrograms of plasmids encoding the full-length McMillan clone^60^ were digested with NotI, and the linearized DNA was extracted using the phenol-chloroform method. One microgram of the linearized DNA was used as the template for *in vitro* transcription using mMESSAGE mMACHINE T7 (Invitrogen). Vero E6 cells were detached using 0.25% (v/v) trypsin-EDTA, washed three times in Dulbecco’s phosphate-buffered saline (DPBS), and resuspended in DPBS in a 4 mm gap cuvette. *In vitro* transcribed RNA was added to the cells. The mixture of cells and RNA were subjected to three 250 V, 10 ms pulses at 1s intervals in an ECM380 square wave electroporation system (BTX). Cells were then incubated at room temperature for 10 min. Subsequently, cells were transferred to a culture flask in culture medium with reduced FBS, and maintained at 37 °C with 5% CO_2_. Two days post-electroporation, cytopathic effects were observed, and the culture supernatant was harvested, centrifuged to clear cellular debris, and stored at −80 °C.

The titer of WEEV McMillan was determined by plaque assays. The virus stock was serially diluted tenfold in DPBS supplemented with 2% (v/v) FBS and used to inoculate confluent monolayers of Vero E6 cells in 12-well plates for 1 h at 37 °C with 5% CO_2_. Cells were then overlaid with minimum essential medium (MEM) (Gibco) supplemented with 2% (v/v) FBS, 1% (v/v) GlutaMax (Gibco), 1% (v/v) sodium bicarbonate (7.5% solution) (Gibco), 1% (v/v) penicillin-streptomycin, and 0.4% LE agarose (Promega). Plates were incubated for two days at 37 °C with 5% CO_2_ prior to fixation with 10% (v/v) buffered formalin (Thermo Fisher Scientific). Monolayers were stained with 1% (v/v) crystal violet (Sigma) and plaques were visualized with the aid of a light box.

#### Production and purification of virus-like particles

The genes encoding the structural polyprotein (capsid-E3-E2-(6K/TF)-E1) of WEEV strains CBA87 (GenBank: DQ432026.1), McMillan (GenBank: DQ393792.1), and Imperial 181 (GenBank: GQ287641.1) with capsid K67N mutation used to improve the yield of VLP production^32^ were separately cloned into the pVRC vector. We also produced EEEV VLPs using a vector that encodes the structural polyprotein of EEEV strain PE6 (GenBank: AAU95735.1) with capsid mutation K67N.^32^ Expi293F^™^ (Thermo Fisher Scientific) cells were cultured at 37 °C with 5% CO_2_ in the Expi293^™^ expression medium. We transfected Expi293F^™^ cells with pVRC vectors encoding WEEV structural polyproteins using ExpiFectamine^™^ 293 Transfection Kit (Thermo Fisher Scientific) according to the manufacturer’s instructions. We collected culture supernatant 5 d post-transfection and pelleted cells by centrifugation at 3,000 x *g* for 20 min. The clarified supernatant was ultracentrifuged with a sucrose cushion consisting of 5 ml of 35% (w/v) sucrose and 5 ml of 70% (w/v) sucrose at 110,000 x *g* for 5 h in a Beckman SW28Ti rotor at 4 °C. The VLPs were pooled from the interface of the 35% (w/v) and 70% (w/v) sucrose cushions, then buffer exchanged to lower the sucrose concentration to less than 20% (v/v) at a volume of 1 ml using a 100-kDa Amicon filter (Sigma). The VLPs were loaded onto a 20%–70% (v/v) continuous sucrose density gradient and centrifuged for 1.5 h in a Beckman SW41 rotor at 210,000 x *g* at 4 °C. The VLP band was collected and exchanged into DPBS (Thermo Fisher Scientific Cat#: 14190–144) using a 100-kDa Amicon filter. We confirmed integrity and purity of VLPs using SDS-PAGE (Figures S2A and S7A).

#### Expression and purification of recombinant proteins

The genes encoding the EC1 domain of human PCDH10 (residues 19–122, GenBank NP_116586.1),^15^ the human VLDLR LBD (residues 31–355, GenBank NP_003374.3),^34^ truncated human VLDLR LA(1–2) (residues 31–110), or the human MXRA8 ectodomain (residues 20–337, GenBank NP_001269511.1)^34^ with the human IgG1 Fc region at their C termini were separately cloned into the pVRC vector. Design VLDLR LA(1–2)-Fc was based on a prior study and included a GGGSGGS linker.^25^ We subcloned the EC1 domain of *P. domesticus* PCDH10 (residues 19–122, GenBank XP_064272571.1) into the same pVRC vector, provided by A. Schmidt.^70^ To purify PCDH10_EC1_-Fc and MXRA8_ect_-Fc, we transfected Expi293F^™^ cells with plasmids encoding the fusion proteins using ExpiFectamine^™^ 293 Transfection Kit (Thermo Fisher Scientific Cat#: A14525) according to the manufacturer’s instructions. Supernatants were collected 5 d post-transfection, centrifuged at 4,000 x *g* for 30 min and purified with MabSelect^™^ PrismA protein A affinity resin (Cytiva Cat#: 17549801) using the manufacturer’s protocol. The proteins were further purified by size-exclusion chromatography on a Superdex 200 increase 10/300 column (Cytiva). Proteins were stored in Tris Buffered Saline (TBS) (20 mM Tris, 150 mM NaCl, pH 7.5).

To purify VLDLR_LBD_-Fc, we co-transfected Expi293F cells with the pVRC vector encoding VLDLR_LBD_-Fc and a pCAGGS vector encoding the chaperon RAP (residues 1–353, GenBank NP_002328). Supernatants were collected 5 d post-transfection and purified with MabSelect^™^ PrismA protein A affinity resin. We separated the VLDLR_LBD_-Fc from RAP on the column by washing the column with 300 volumes of 10 mM EDTA in TBS overnight. Then VLDLR_LBD_-Fc was refolded on the column by washing with 100 column volumes of 2 mM CaCl_2_ in TBS and eluted using the manufacturer’s protocol. The proteins were concentrated and further purified by size-exclusion chromatography on a Superdex 200 increase 10/300 GL column. RAP was stored in TBS and VLDLR_LBD_-Fc was stored in TBS containing 2 mM CaCl_2_. VLDLR LA(1–2)-Fc was similarly generated. Control IgG (C1A-H12 anti-SARS-CoV-2 spike protein antibody) was generated based on a prior study^71^. Proteins used for in vivo experiments were not subjected to size exclusion chromatography other than for a small aliquot analyzed for quality control purposes, which confirmed that the material eluted as a single peak. Proteins were also tested for the presence of endotoxin, which was measured as <0.1 endotoxin units ml^–1^ using a Pierce Chromogenic Endotoxin Quantification Kit (Thermo Fisher Scientific).

The genes encoding the EC1 domain of human PCDH10 (residues 19–122, GenBank NP_116586.1) were cloned into a pVRC vector with a C-terminal twin-strep tag for purification. We transfected Expi293F^™^ cells with plasmid using ExpiFectamine^™^ 293 Transfection Kit according to the manufacturer’s instructions. After centrifugation at 4,000 x *g* for 30 min, supernatants were incubated with Strep-Tactin^®^ XT Sepharose resin (IBA Lifesciences) for 1 h at 4 °C. The beads were then washed using TBS, and the bound proteins were eluted using 50 mM biotin in TBS. The proteins were further purified by size-exclusion chromatography on a Superdex 200 increase 10/300 column equilibrated with TBS.

#### Biolayer interferometry binding assays

We performed biolayer interferometry experiments with an Octet RED96e (Sartorius) and analyzed data using ForteBio Data Analysis HT version 12.0.1.55 software. The anti-WEEV E2–E1 monoclonal antibody SKW11 (a gift from M. Sutton and M. Roederer)^59^ was loaded onto Anti-Human IgG Fc Capture (AHC) Biosensors (Sartorius Cat#: 18–5063) at a concentration of 250 nM in kinetic buffer (TBS supplemented with 2 mM CaCl_2_, 0.1% (w/v) bovine serum albumin and 0.01% Tween) for 600 s. After a baseline measurement in kinetic buffer for 60s, tips were dipped into wells containing 1 μM WEEV CBA87 VLPs for 1 h. The signal was then allowed to equilibrate for another 1 h in kinetic buffer. The tips were washed with the kinetic buffer for 60 s to obtain a baseline reading, then the biosensors were dipped into wells containing the serial dilutions of human PCDH10_EC1_-twin-strep (concentration range of 10 μM to 312.5 nM) in kinetic buffer for 900 s. Finally, a 300 s dissociation in kinetic buffer was performed. Data analysis was performed using a standard 1:1 binding model.

#### ELISA

Apparent affinities of *Hs*PCDH10 EC1-Fc and *Pd*PCDH10_EC1_-Fc for CBA87 and Imperial 181 VLPs were also separately determined by ELISA. Two hundred nanograms of VLPs were immobilized on ELISA MaxiSorp plates (Thermo Scientific Cat#: 439454) overnight at 4 °C. The coated plates were blocked by PBS supplemented with 3% (w/v) bovine serum albumin for 1 h at room temperature, then were washed five times with PBS. Serial dilutions of *Hs*PCDH10_EC1_-Fc or *Pd*PCDH10_EC1_-Fc proteins were prepared and added to plates and incubated for 1 h at room temperature. After incubation with Fc-fusion proteins, the plates were washed five times with PBS, and then 100 μl per well of horseradish-peroxidase-conjugated anti-Human IgG (Sigma Cat#: A0170) diluted at ratio of 1:20,000 in PBS supplemented with 3% bovine serum albumin were added for incubation for 1 h at room temperature. The plates were washed five times with PBS, then incubated with 100 μl per well of 1-step TMB ELISA solution for 3 min at room temperature in the dark and the reactions were stopped by addition of 100 μl per well of 2 N sulfuric acid. Absorbance was read at an optical density of 450 nm using BioTek multi-mode reader. EEEV PE6 VLPs were used as negative controls in experiments.

#### Truncation and mutagenesis, and stable cell line generation

K562 cells stably transduced to express human PCDH10 (GenBank NM_032961.3), human VLDLR (GenBank NP_003374.3), human MXRA8 (GenBank NM_032348.3), human ApoER2 isoform 2 (GenBank NM_004631.5), *P. domesticus* PCDH10, *E. caballus* PCDH10 (GenBank XM_023636548.1), *T. sirtalis* PCDH10 (GenBank XM_014072689.1), *M. musculus* PCDH10 (GenBank: NP_001091640.1) were generated in a prior study where cell-surface receptor expression was confirmed.^15^ *P. domesticus* MXRA8^15^ was cloned into a lentiGuide-Puro (Addgene: #52963, gift from Feng Zhang)^72^ expression vector with the exception that the N-terminal Flag tag was omitted.

For the single PCDH10 EC1 construct, residues 19–690 in the PCDH10 precursor protein were replaced with residues 19–122 and adding a Flag tag (DYKDDDDK) between S696 and G697. For the *Hs*PCDH10 (*Pd*PCDH10 EC1) chimera, residues 19–122 in the *Hs*PCDH10 precursor protein were replaced with residues 19–122 in the *Pd*PCDH10 precursor protein. For PCDH10 stalk-Flag, residues 19–690 in the precursor protein sequence were removed and a Flag tag (DYKDDDDK) was added between S696 and G697. Point mutations were generated on the background of the EC1 construct or the full-length PCDH10 using site-directed mutagenesis. VLDLR truncation constructs were generated based on a prior study.^24^ WT and mutant PCDH10 and VLDLR constructs were cloned into a lentiGuide-Puro (Addgene #52963) expression vector.

To generate lentiviruses for stable transduction, lentiGuide-Puro plasmids containing the transgene were co-transfected with psPAX2 (Addgene #12260) and PMD2.G (Addgene #12259) at a ratio of 3:2:1 into HEK 293T cells using Lipofectamine 3000 (Thermo Fisher Scientific). Lentiviruses were harvested 2 d post-transfection and used to transduce K562 cells. Successfully transduced K562 cells were selected for using puromycin at 2 μg ml^−1^. Cell lines were confirmed to express the constructs of interest at the plasma membrane by cell surface antibody staining. Antibodies are listed in the key resources table.

#### Cell surface antibody staining

Primary antibodies used in cell surface receptor staining include polyclonal anti-PCDH10 (Proteintech 21859–1-AP), anti-VLDLR (GeneTex GTX79552), anti-MXRA8 (MBL International W040–3), rabbit IgG isotype (Proteintech 30000–0-AP) and mouse IgG isotype (BD Biosciences BDB557351). Cells were incubated in blocking buffer (5% (v/v) goat serum in PBS) for 30 min at 4°C. Primary antibodies were diluted to 10 μg ml^−1^ in binding buffer (2% (v/v) goat serum in PBS) immediately before use. Following blocking, cells were washed once in binding buffer and subsequently incubated with primary antibodies (10 μg ml^−1^) in binding buffer for 30 min at 4°C. Cells were washed three times in binding buffer and subsequently incubated with a PE-conjugated donkey anti-rabbit F(ab’)_2_ fragment (Jackson ImmunoResearch 711–116-152) or a PE-conjugated donkey anti-mouse F(ab’)_2_ fragment (Jackson ImmunoResearch 715–116-150) diluted 1:200 in binding buffer for 30 min at 4 °C. Cells were washed twice in binding buffer and twice in PBS, fixed in 2% (v/v) formalin and subjected to detection of surface staining using an iQue3 Screener PLUS (Intellicyt) with ForeCyt (Sartorius) software. Antibody staining was visualized using FlowJo (version 10.6.2).

For cells expressing Flag-tagged constructs, following blocking as described above, cells were incubated with an APC-conjugated anti-DYKDDDDK (Flag) antibody (BioLegend 637307) or an APC-conjugated control antibody (BioLegend 402306) at 5 μg ml^−1^ in binding buffer for 30 min at 4°C. Cells were then washed twice in binding buffer, twice in PBS. Surface receptor expression was detected using an iQue3 Screener PLUS (Intellicyt) with IntelliCyt ForeCyt Standard Edition version 8.1.7524 (Sartorius) software. Antibody staining was visualized using FlowJo (version 10.6.2).

#### Reporter virus particle generation

RVPs were generated based on a prior study^34^ using a dual vector system: a modified pRR64 Ross River virus replicon^73^ provided by R. Kuhn (Purdue University) (the SP6 promoter is replaced with a CMV promoter, the E3-E2–6K/TF-E1 sequence is replaced with a turbo GFP reporter preceded by a porcine teschovirus-1 2A self-cleaving peptide), and a pCAGGS vector expressing heterologous WEEV or HJV E3-E2–6K/TF-E1 proteins. The two vectors were co-transfected into HEK 293T cells using Lipofectamine 3000 (Thermo Fisher). Four to six hours post-transfection, we replaced media with Opti-MEM (Thermo Fisher) supplemented with 5% (v/v) FBS, 25 mM HEPES, and 5 mM sodium butyrate. We harvested supernatant 2 d post-transfection, centrifuged supernatant at 4,000 rpm for 5 min, filtered using a 0.45 μm filter, and froze aliquots at −80 °C for storage.

WEEV E3-E2–6K/TF-E1 coding sequences cloned into the pCAGGS vector include: 71V1658 (GenBank NC_003908.1), Fleming (GenBank MN477208.1), McMillan (GenBank GQ287640.1), Imperial 181 (GenBank GQ287641.1), CBA87 (GenBank KT844543.1), BFS09997 (GenBank KJ554974.1), EP6 (GenBank KJ554967.1), AG80–646 (GenBank NC_075015.1), TR25717 (GenBank KT844541.1), EQ1090 (GenBank PP544260.1), DILAVE218 (GenBank PP620644.1). For HJV, strain 585–01 (GenBank NC_012561.1) was used. Mutant E3-E2–6K/TF-E1 sequences were generated using site-directed mutagenesis.

Titration of RVPs for MOI calculation was performed on Vero E6 cells seeded in a 96-well plate using a serial tenfold dilution of the RVP stocks. Twenty-four hours post-infection, numbers of GFP-positive cells were counted and used to calculate RVP titer as infectious unit per milliliter (IU ml^−1^), assuming that at high dilution factors, each GFP-positive cell is infected with one RVP given that RVPs only infect cells for one cycle.

#### Reporter virus particle entry assays

We incubated transduced K562 cells with RVPs. Twenty-four hours post-infection, cells were harvested, washed twice with PBS, and fixed in PBS containing 2% (v/v) formalin. GFP expression was measured by flow cytometry using an iQue3 Screener PLUS (Intellicyt) with IntelliCyt ForeCyt Standard Edition version 8.1.7524 (Sartorius) software. An example of the flow cytometry gating scheme used to quantify GFP-expressing RVP infection is provided in Figure S6C.

For assessment of efficiency of receptor recognition, RVPs were titrated on K562 cells expressing cognate receptors in a twofold dilution series, with multiplicity of MOI calculated based on titers measured on Vero E6 cells.

#### WEEV RVP neutralization assays with Fc fusion proteins

We pre-incubated GFP-expressing WEEV RVPs in the presence of Fc fusion proteins in culture medium for 30 min at 37 °C. The mixtures were added to the cells. Twenty-four hours post-infection, cells were washed twice in PBS and fixed in 2% (v/v) formalin. RVP entry was measured using an iQue3 Screener PLUS (Intellicyt) with IntelliCyt ForeCyt Standard Edition version 8.1.7524 (Sartorius) software. We calculated relative infection as follows: Relative infection (%) = (percentage of GFP-positive cells in the presence of Fc fusion proteins)/(percentage of GFP-positive cells in the absence of Fc fusion proteins) × 100.

#### Mouse cortical neuron culture and infection

Embryonic day 17 mouse cortical neurons were purchased (Thermo Fisher Scientific Cat#: A15586) and cultured according to the manufacturer’s protocol with modifications. Briefly, neurons were thawed and plated on 96 well plates coated with Poly-D-Lysine at 4.5 μg cm^−2^ at 40,000 neurons per well, in neurobasal medium (Thermo Fisher Scientific Cat#: 21103) supplemented with 0.5 mM Glutamax (Thermo Fisher Scientific Cat#: 35050), 2% (v/v) B-27 (Thermo Fisher Scientific Cat#: 17504), and 10 μM Y-27632 (Stemcell Technologies Cat#: 72302). Twenty-four hours post-plating, a full medium change was performed to remove Y-27632. Half medium change was performed every third day until infection. Neurons were infected with WEEV Imperial 181 WT or mutant RVPs at an MOI of 2 in the presence or absence of a control IgG (316 μg ml^−1^), PCDH10_EC1_-Fc (316 μg ml^−1^), or RAP (100 μg ml^−1^). Eight hours post-infection, neurons were scanned using the Incucyte S3 Live Cell Imaging system (Sartorius) with Incucyte S3 Software version 2023A Rev2 (Sartorius) using a 20× objective. GFP-positive neurons were scored as cells with a threshold signal greater than 3 green calibrated units (GCU) above background, using a Surface Fit background subtraction method. The neuronal cell body area in each image was obtained by analyzing phase-contrast images using the Incucyte S3 Software (version 2023B). To calculate the percentage of positive cells, at the time point of 8 h post-infection, the area of GFP signal above background was divided by the total area covered by neuronal cell bodies and was multiplied by 100. Relative infection was calculated as follows: Relative infection (%) in the presence of recombinant proteins = (Percentage of GFP-positive cells in the presence of recombinant proteins) / (Percentage of GFP-positive cells under mock treatment) × 100.

#### Replication kinetics assay with HJV

K562 cells (2.5 × 10^6^) transduced to overexpress human PCDH10, sparrow PCDH10, or human MXRA8 were pelleted by centrifugation at 1,388 rpm for 2 min, after which the cell pellets were resuspended in 1 ml of maintenance medium (RPMI1640 supplemented with 2% (v/v) FBS, 25 mM HEPES, 1% (v/v) penicillin-streptomycin) containing HJV (strain B230) at an MOI of 0.01. The infection was allowed to proceed for 1 h at 37 °C and 5% CO_2_, after which the cells were washed three times with 10 ml DPBS (Sigma) by resuspension followed by centrifugation. The cells were then resuspended in 5 ml maintenance medium. Immediately following this final resuspension, and again at 6, 12, 24 and 48 h post-infection, 500 μl supernatant was collected from each sample and stored at −80 °C. The removed volume was replaced with 500 μl fresh maintenance medium each time, and samples were returned to the incubator. Sample titers were determined by TCID_50_ on Vero E6 cells.

For tissue culture infectious dose 50 (TCID_50_) assays, culture samples containing HJV were serially diluted tenfold in Dulbecco’s modified Eagle’s medium (DMEM, Gibco) supplemented with 10% (v/v) FBS and 25 mM HEPES (Thermo Fisher Scientific). Diluted samples were added to monolayers of Vero E6 cells in a 96-well format, with 8 wells infected for each dilution. Infection was allowed to proceed for 5 days. TCID_50_ was calculated as the dilution factor showing cytopathic effect in 50% of wells (4 out of 8) by using the Spearman-Kärber method^74^.

#### *In vivo* protection study

Mouse studies were performed as approved by the University of Texas Medical Branch Institutional Animal Care and Use Committee (protocol number 1708051) in accordance with the NIH Guidance for the Care and Use of Laboratory Animals. Mice were fed a 19% protein diet (Teklad, 2919, Irradiated), had 12 h light:dark cycle (06:00–18:00), and were housed in a facility maintained at a temperature range of 20–26 °C with a humidity range of 30–70%. Food and water were provided *ad libitum*. Sample sizes for mouse studies were determined based on previously published results for similar in vivo experiments.^15^ Mixed-sex cohorts (*n* = 5 female and *n* = 5 male) of seven-to-eight-week-old CD-1 IGS mice (Charles River, Wilmington, MA) received a 25 mg kg^−1^ dose of either VLDLR LA(1–2)-Fc, isotype control IgG (anti-SARS-CoV-2 spike protein antibody C1A-H12)^71^, or buffer diluent (Tris-buffered saline (TBS) supplemented with 2 mM CaCl_2_) through the intraperitoneal route. Six hours later, all mice were infected with 1,000 PFU WEEV McMillan via the subcutaneous route in the left rear footpad. Health checks and weight measurements were performed daily up to 14 days post-infection. We were not blinded to the treatment or infection status of the mice, also for safety reasons, since WEEV can cause severe disease in humans.

#### Cryo-EM sample preparation and data collection

We first mixed 3 μl of WEEV VLPs at a concentration of 3.0 mg ml^−1^ in DPBS (Thermo Fisher Scientific Cat#: 14190–144) with 3 μl of *Hs*PCDH10_EC1_-Fc protein at 3.0 mg ml^−1^, then immediately applied 3 μl of sample to glow-discharged Quantifoil grids (R 0.6/1 300 mesh, gold, EMS Cat#: Q350AR-06) and blotted once for 5 s after a wait time of 15 s in 100% humidity at 4 °C and plunged into liquid ethane using an FEI Vitrobot Mark IV (Thermo Fisher Scientific). WEEV Imperial 181 VLPs were also incubated with *Pd*PCDH10_EC1_-Fc for 4 h, and samples were frozen on Quantifoil grids (R 2/2 300 mesh, gold, EMS Cat#: Q3100AR2). WEEV McMillan VLPs were also incubated with VLDLR_LBD_-Fc, and samples were immediately frozen on Quantifoil grids. We also applied 3 μl of WEEV CBA87 VLP sample onto glow-discharged Quantifoil grids and plunged into liquid ethane to prepare cryo-EM samples with same parameters as WEEV VLP:PCDH10_EC1_-Fc complex using an FEI Vitrobot Mark IV. Cryo-EM data collection was performed using a 300 kV FEI Titan Krios microscope (Thermo Fisher Scientific) equipped with a K3 (Gatan) or a Falcon 4 (Thermo Fisher Scientific) direct electron detector at the Harvard Cryo-Electron Microscopy Center. Automated single-particle data acquisition was performed with SerialEM or EPU, with a magnification of ×81,000 (K3) or ×130,000 (Falcon 4) in counting mode, which yielded a calibrated pixel size of 1.06 Å (K3) or 0.94 Å (Falcon 4) and the datasets were collected at defocus ranges of −0.6 to −1.8 μm or −0.8 to −1.8 μm.

#### Cryo-EM data processing

Raw movie stacks were corrected for beam-induced motion using MotionCor2 (version 1.6.4).^64^ The parameters of the contrast transfer function (CTF) for all micrographs were estimated by CTFDIND-4.1 (version 4.1.14).^65^ For the WEEV CBA87 VLP in complex with *Hs*PCDH10_EC1_-Fc, a total 22,075 particles were auto-picked from 7,749 micrographs using crYOLO (version 1.8.2),^62^ and particles were extracted with binned two times (pixel size 2.12 Å) in RELION 3.1 (version 3.1.4).^61^ After several rounds of reference-free 2D classification, 19,527 particles were selected from good 2D classes and subjected to 3D classification with icosahedral symmetry, and these particles were classified into five classes using the 12 Å density map of WEEV VLP (EMD-5210)^75^ as the initial reference model. After 3D classification, a total of 13,141 particles were selected and subjected to further 3D auto-refinement with icosahedral symmetry, finally yielding 4.8 Å reconstruction of the WEEV CBA87 VLP:*Hs*PCDH10_EC1_-Fc complex. To improve the resolution of the map, we used the block-based reconstruction method^76^ centered on the q3 E2–E1 trimer. 788,460 blocks, each comprising four E2–E1 trimers near quasi-three-fold axes (q3), were extracted and subjected to 3D classifications without alignment. 343,889 particles were selected for initial refinement with C1 symmetry, which was followed by CTF refinement, and a final round of refinement that generated a 3.3 Å map of the q3 E2–E1 trimers with their associated capsids. We then used masked refinement to obtain a 2.9 Å density map of a E2–E1 trimer with their associated capsid protomers. This map was processed using DeepEMhancer (version 20210511)^63^ for cryo-EM volume post-processing to generate a map for model building. Additional information regarding the workflow, number of images, and particles is found in Figure S3A and Table S2.

The cryo-EM WEEV VLP alone data were processed with similar data processing procedure using Relion 3.1 (version 3.1.4),^61^ which generated a 5.4 Å map of the WEEV CBA87 VLP alone. We then obtained a 3.4 Å map of the q3 E2–E1 trimers with their associated capsids by block-based reconstruction method.^76^ This map was processed using DeepEMhancer (version 20210511)^63^ for cryo-EM volume post-processing to generate a map for model building. Additional information regarding the workflow, number of images, and particles is found in Figure S3B and Table S2. The cryo-EM dataset of WEEV Imperial 181 VLP bound to *Pd*PCDH10_EC1_-Fc data was processed with a similar approach using RELION 3.1 (version 3.1.4),^61^ which generated a 4.8 Å map of the WEEV Imperial 181 VLP bound to *Pd*PCDH10_EC1_-Fc. We then obtained a 2.8 Å map of a E2–E1 trimer bound to *Pd*PCDH10_EC1_-Fc using the block-based reconstruction method.^76^ Additional information regarding the work-flow, number of images, and particles is found in Figure S7B and Table S2.

For the WEEV McMillan VLP in complex with VLDLR_LBD_-Fc, we processed the first dataset with similar data processing steps and got a 5.9 Å map of the WEEV McMillan VLP:VLDLR_LBD_-Fc. To improve the quality of the density map, we collected a second dataset. The second dataset was processed separately and generated a 4.8 Å map of the WEEV McMillan VLP:VLDLR_LBD_-Fc. We then combined VLPs from both datasets after the first round of refinement and subjected them to refinement. Finally, the resolution of the combined WEEV McMillan VLP:VLDLR_LBD_-Fc complex map was 4.7 Å. We performed block-based reconstruction of q3 E2–E1 trimers with associated capsids of both datasets independently. We combined good blocks in the 3D classification results of both datasets and 1,720,156 blocks were used to perform 3D auto-refinement, which generated a 2.9 Å map of the q3 E2–E1 trimers and associated capsids. To improve features for the LA repeat density, an asymmetric unit masked refinement was performed. The resolution of masked refinement map is 2.8 Å, and DeepEMhancer-post-processing yielded a density map for model building with details of the E2–E1 heterodimers and capsid that were well-resolved. Maps were processed using DeepEMhancer (version 20210511)^63^ for cryo-EM volume post-processing. Additional information regarding the workflow, number of images, and particles is found in Figure S10A and Table S2.

#### Model building

For the WEEV CBA87 VLP:*Hs*PCDH10_EC1_-Fc, we used coordinates of PCDH10 EC1 from the crystal structure of human PCDH10 EC1–EC4 (PDB:6VFQ)^19^ and of WEEV E2–E1 and capsid predicted by AlphaFold2^69^ as initial models. These models were docked into the DeepEMhancer post-processed cryo-EM density map using UCSF Chimera (version 1.6.1).^66^ The atomic model was then generated through iterative rounds of model building and adjustment in *Coot* (version 0.9.8.91)^67^ and refined using real space refinement in Phenix (version 1.21rc1–5127).^68^

The model of the unliganded WEEV CBA87 VLP was generated based on WEEV CBA87 VLP:*Hs*PCDH10_EC1_-Fc model that was docked into the DeepEMhancer post-processed cryo-EM density map of the unliganded VLP using UCSF ChimeraX. For the WEEV Imperial 181 VLP:*Pd*PCDH10_EC1_-Fc complex, the structure CBA87 VLP:*Hs*PCDH10_EC1_-Fc as an initial model was fitted into the DeepEMhancer post-processed cryo-EM map of a E2–E1 trimer for the Imperial 181 complex. The initial coordinates of WEEV McMillan VLP:VLDLR_LBD_-Fc were generated based on WEEV CBA87 VLP:*Hs*PCDH10_EC1_-Fc model and VLDLR LA(1–2) structure predicted by AlphaFold2.^69^ These models were docked into the cryo-EM map of the WEEV McMillan: VLDLR_LBD_-Fc complex using UCSF ChimeraX.

The atomic models for the unliganded WEEV CBA87 VLP, WEEV Imperial 181 VLP:*Pd*PCDH10_EC1_-Fc, WEEV McMillan VLP:VLDLR_LBD_-Fc were generated through iterative rounds of model building and adjustment with their associated maps in *Coot* (version 0.9.8.91)^67^ and refined using real space refinement in Phenix (version 1.21rc1–5127).^68^ All models were validated using RCSB PDB and refinement statistics are provided in Table S2. Figures of structural presenting were made by either UCSF ChimeraX (version 1.6.1)^66^ or PyMOL (version 3.0.2) (https://www.pymol.org/pymol). Software used in this project was curated by SBGrid.^77^

#### Biosafety considerations

All studies examining the receptor-binding properties of alphavirus E2–E1 glycoproteins, including those with mutations that modulate receptor binding, were conducted using RVPs, which are single-cycle, non-replicative particles. The *in vivo* protection study, which used WT infectious WEEV McMillan, was performed under strict biosafety (biosafety level 3) guidelines at the University of Texas Medical Branch (UTMB). Appropriate safety measures were implemented to protect both researchers and animals.

### QUANTIFICATION AND STATISTICAL ANALYSIS

Data were deemed statistically significant when P values were <0.05 using version 10 of GraphPad Prism. Experiments were analyzed by one-way or two-way ANOVA with multiple comparison correction, or by log-rank (Mantel-Cox) test.

## Supplementary Material

1 Figure S1. Phylogenetic tree of WEEV strains and Highlands J virus, related to Figure 1.
Maximum likelihood phylogenetic tree of 57 WEEV strains using the coding sequences of the structural polyprotein genes. Highlands J virus strain 64A-1519 (GenBank: KT429021) is also included. Scale bar represents 0.05 nucleotide substitutions per site. SFV strain SFV4, VEEV strain TC-83, and Madariaga virus (MADV) strain 267113 were included in the phylogenetic analysis (not shown). Numbers at nodes indicate bootstrap values. In cases in which the branches are too small, bootstrap values may not be shown. The three lineages (A, B, and C) and B sublineages (B1, B2, and B3) are indicated. Taxon labels include strain name and year of isolation. GenBank accession numbers are provided in Table S1.

2 Figure S2. Binding experiments with PCDH10 EC1 and WEEV VLPs, related to Figures 1 and 2.
(A) Coomassie-stained SDS-PAGE gel of purified VLPs. The experiment was performed twice, and representative gel images are shown.
(B) Schematic diagrams of the PCDH10_EC1_-Fc, the PCDH10_EC1_ twin-strep tag, VLDLR_LBD_-Fc and VLDLR LA(1–2)-Fc constructs.
(C) Size exclusion chromatography of Fc-fusion and twin-strep tagged proteins. Insets are SDS-PAGE gels of pooled peak fractions visualized using a stain-free imaging system.
(D) Biolayer interferometry binding analysis of *Hs*PCDH10_EC1_-twin-strep with immobilized WEEV CBA87 VLPs.
(E) Scatchard plot for the biolayer interferometry binding analysis of *Hs*PCDH10_EC1_-twin-strep with WEEV CBA87 VLPs shown in (D). The measured apparent affinity is indicated.
(F) Representative ELISA results showing the binding of the *Hs*PCDH10_EC1_-Fc or *Pd*PCDH10_EC1_-Fc to WEEV CBA87, WEEV Imperial 181, or EEEV FL91–469 VLPs. Data shown are mean ± s.d. from one experiment performed with technical triplicates. The experiment was performed three times (*n* = 3) and representative data are shown. EC_50_ values shown are the mean of three independent experiments.

3 Figure S3. Cryo-EM reconstructions of WEEV CBA87 VLPs alone or bound to human PCDH10_EC1_-Fc, related to Figure 1.
(A) Workflow used for cryo-EM data processing of WEEV CBA87 VLPs bound to human PCDH10_EC1_-Fc.
(B) Workflow used for cryo-EM data processing of unliganded WEEV CBA87 VLPs.
(C and D) 3D representation of angular distribution of particles and local resolution map estimates using Relion of the WEEV CBA87 with PCDH10_EC1_-Fc complex (C) or WEEV CBA87 VLP alone (D).
(E) Top and side views of a representative q3 block of the WEEV CBA87 VLPs, displayed on the local resolution map.
(F and G) Fourier shell correlation curves of WEEV CBA87 VLP bound to PCDH10_EC1_-Fc (F) or WEEV CBA87 VLP alone (G) are shown. The threshold used to estimate the resolution is 0.143.
(H) Buried surface area (BSA) calculations for alphavirus E2–E1 receptor complexes described here and in prior publications^22–25,27,43^.
(I and J) Structural superposition of the X-ray crystal structure of PCDH10 EC1–4 homodimer (PDB ID: 6VFW)^19^ with the WEEV CBA87:PCDH10_EC1_-Fc complex. Panel (I) shows that there is no conformational change in PCDH10 EC1 when comparing the WEEV E2–E1 bound EC1 structure to EC1 within the EC1–EC4 homodimer. (J) Highlights steric clashes between one copy of EC1–EC4 in the EC1–EC4 homodimer and WEEV E2–E1.
(K) Human PCDH10 EC1 in ribbon rendering (left) or surface rendering (right) in the same orientation. Contact residues for WEEV E2–E1 and for EC4 in an antiparallel PCDH10 homodimer are shown in magenta and cyan respectively. Overlapping contact residues are shown in orange.
(L) Superposition of the X-ray crystal structure of PCDH10 EC1–6 (PDB ID: 6VG4)^19^ onto the EC1 protomers bound to the E2–E1 trimer of WEEV CBA87. Occupancy of the three receptor-binding sites on the trimer would result in steric hindrance between the EC2 repeats of neighboring receptor molecules. The left panel shows a top view, and the right panel shows a side view.

4 Figure S4. Representative cryo-EM density maps of WEEV E2–E1 bound to alternate receptors and domains involved in receptor binding, related to Figures 1, 2 and 3.
(A–C) Density maps of the indicated polypeptide segments from the following complexes: WEEV CBA87 VLP bound to *Hs*PCDH10_EC1_-Fc (A), WEEV Imperial 181 VLP bound to *Pd*PCDH10_EC1_-Fc (B), or WEEV McMillan VLP bound to VLDLR_LBD_-Fc (C).
(D–F) Ribbon diagrams of WEEV E2–E1 heterodimers bound to surface-rendered PCDH10 EC1 or VLDLR LA(1–2). E2 domains (domains A, B, and C) and E1 domains (DI–III) are indicated in different colors. FL: fusion loop.

5 Figure S5. Sequence alignments of alphavirus E2–E1 glycoproteins and protocadherin EC1 repeats, related to Figures 1, 2 and 7.
(A and B) Sequence alignments of the E2 (A) and E1 (B) glycoproteins of WEEV CBA87 (GenBank: DQ432026.1), WEEV 71V658 (GenBank: NP_640331.1), WEEV McMillan (GenBank: DQ393792.1), Imperial 181 (GenBank: GQ287641), EEEV FL91–469 (GenBank: Q4QXJ7.1), VEEV TC83 (GenBank: AAB02517.1), and SFV4 (GenBank: AKC01668.1). E2 and E1 residues that are with 4 Å of the indicated receptors in the structures are shown as indicated in the legend, and essential basic residues contacting LA repeats based on prior structural studies of LA repeat bound alphavirus E2–E1 proteins^22,24–27,40^ are highlighted in green. Residues that are completely conserved in all aligned sequences have a red background. Boxed residues show positions where a single majority residue or multiple chemically similar residues are found. Such residues are in red. Domains of E2 and E1 are indicated above the sequences.
(C) Sequence alignments of PCDH10 EC1 orthologs of *H. sapiens* PCDH10 (GenBank: NP_116586.1), *M. musculus* PCDH10 (GenBank: NP_001091642.1), *E. caballus* PCDH10 (GenBank: XP_023492316.1), *P. domesticus* PCDH10 (GenBank: XP_064272564.1), and *T. sirtalis* PCDH10 (GenBank: XP_013928164.1).
(D) Sequence alignments of human PCDH10 EC1 with the EC1 repeats of other non-clustered δ2 protocadherins PCDH17 (GenBank: NP_001035519.1) and PCDH19 (GenBank: NP_001171809.1). Residues that are completely conserved have a gray background. Boxed residues highlight positions where a single majority residue or multiple chemically similar residues could be identified. Such residues are highlighted in pink. The panels were generated using ESPript 3.0.^58^ PCDH10 residues that are with 4 Å of the indicated E2 or E1 proteins in the structures are shown as indicated in the legend.

6 Figure S6. Gating strategy, cell surface receptor staining, and WEEV McMillan VLDLR LA repeat dependencies, related to Figures 1, 2 and 3.
(A) Example of flow cytometry gating strategy used to quantify cells stained by antibodies. The example shown is for K562 cells expressing the PCDH10 stalk-FLAG construct.
(B) Cell surface immunostaining of K562 cell expressing PCDH10 truncation constructs by APC-conjugated anti-Flag or isotype control antibodies.
(C) Example of flow cytometry gating strategy used to quantify cells expressing GFP following infection by GFP-expressing RVPs. The example provided is with WEEV 71V RVP.
(D) Staining of K562 cells expressing the human orthologs of the indicated alphavirus receptors. PE: *R*-phycoerythrin. For staining of K562-ApoER2iso2, cells were incubated with RAP-Flag followed by allophycocyanin (APC)-conjugated anti-Flag or isotype control antibodies.
(E) Staining of K562 cells expressing mutant human PCDH10 containing sparrow PCDH10 polymorphisms.
(F) Schematic diagrams of WT VLDLR and single LA repeat constructs. A ΔLBD construct in which the entire LBD is replaced by an N-terminal Flag tag is used as a negative control in experiments.
(G) Infection of K562 cells stably expressing VLDLR, ΔLBD-Flag, or single LA repeat constructs by WEEV McMillan RVPs. Infection was quantified by flow cytometry. Data are mean ± s.d. from three experiments performed in triplicates (*n* = 3). One-way ANOVA with Dunnett’s multiple comparisons test, ****P<0.0001; ***P<0.001; **P<0.01.
(H) Cell surface immunostaining of K562 cells expressing VLDLR or Flag-tagged VLDLR truncation constructs by APC-conjugated anti-Flag or isotype control antibodies.

7 Figure S7. Cryo-EM reconstruction of WEEV Imperial 181 VLPs in complex with *Pd*PCDH10_EC1_-Fc, related to Figure 2.
(A) Coomassie-stained SDS-PAGE gel of purified WEEV Imperial 181 VLPs. NR: non-reducing. R: reducing.
(B) Workflow used for cryo-EM data processing of WEEV Imperial 181 VLPs bound to *Pd*PCDH10_EC1_-Fc.
(C) 3D representation of the angular distribution of particles and local resolution map estimates generated in Relion of WEEV Imperial 181 VLP in complex with *Pd*PCDH10_EC1_-Fc.
(D) Fourier shell correlation curves of WEEV Imperial 181 VLP in complex with *Pd*PCDH10_EC1_Fc. The threshold used to estimate the resolution is 0.143. See Methods for additional details.
(E) Interface between WEEV Imperial 181 E2–E1 or E2’–E1’ and *Pd*PCDH10 EC1. Residues that participate in interactions between WEEV Imperial 181 E2–E1 heterodimers and *Pd*PCDH10 EC1 are indicated, with polar contacts shown as gray dashed lines.
(F) K562 cells stably expressing human (*Hs*), sparrow (*Pd*) PCDH10 or MXRA8, or horse (*Ec*) PCDH10 were infected with GFP-expressing WT or mutant 71V RVPs. Infection was measured by flow cytometry. Data are mean from three experiments performed in duplicates or triplicates (*n* = 3). Two-way ANOVA with Dunnett’s multiple comparisons test; ****P < 0.0001.
(G) Interface between WEEV McMillan E2 and duck MXRA8 (PDB: 8DAN),^25^ showing E2 L149 and receptor residues in its vicinity. The closest distance between duck MXRA8 residue T117 and atoms on WEEV E2 L149 among the protomers is shown. The distance suggests that the L149Q substitution would be tolerated at the interface.

8 Figure S8. Contact residues, AF3 modeling of HJV–PCDH10 interactions, and summary of mouse virulence of McMillan mutants and the effects of mutagenesis on receptor dependencies, related to Figures 4, 6 and 7.
(A) WEEV E2 or E1 residues that contact PCDH10. WEEV CBA87 E2–E1 residues that contact *Hs*PCDH10 or WEEV Imperial 181 E2–E1 residues that contact *Pd*PCDH10 (< 4.0 Å) are indicated. A key E2 polymorphic residue (Q149) is colored pink.
(B) WEEV E2 or E1 residues that contact VLDLR. Key polymorphic residues (E2 K181 and E2 K81) are colored pink.
(C) pLDDT scores of the interaction interface for the AF3 model of HJV containing E2 A177K substitution in complex with human PCDH10 EC1. Atoms with very high confidence (pLDDT > 90) are shown in deep blue, confidence (90 > pLDDT > 70) in light blue, low confidence (70 > pLDDT > 50) in yellow, and very low confidence (pLDDT < 50) in orange.
(D) Summary of effects of WEEV E2–E1 mutagenesis on receptor-mediated entry into K562 cells. K562 infectivity assays for WEEV WT and mutant RVPs conducted in the current study are summarized. “Entry”, RVP infection with 20–100% GFP-positive cells achieved and significantly higher than infection of K562 cells expressing the negative control human MXRA8 (*P*<0.05); “Weak entry”, RVP infection with 5–20% GFP-positive cells achieved and significantly high than infection of K562 cells expressing human MXRA8 (*P*<0.05); “No entry”, RVP infection levels that are not significantly different than infection levels on K562 cells expressing human MXRA8 (*P*>0.05).
(E) Effects of WEEV E2 McMillan mutations on mortality and mean time-to-death (MTD) when five-week-old female CD1 mice were inoculated 1000 PFU subcutaneously in the left thigh, summarized from Mossel et al.^41^ The expected effects of E2 mutations on PCDH10 or VLDLR/ApoER2 are based on the results of K562 infectivity assays with McMillan RVPs shown in Figure 3G.

9 Figure S9. Sequence alignments of PCDH10 orthologs, related to Figure 2.
Sequence alignments of PCDH10 orthologs of *H. sapiens* (GenBank: NP_116586.1); *M. musculus* (GenBank: NP_001091642.1); *E. caballus* (GenBank: XP_023492316.1); *P. domesticus* (GenBank: XP_064272564.1); *T. sirtalis* (GenBank: XP_013928164.1). Residues that are completely conserved have a red background. The red background denotes residues that are completely conserved in all sequences. Boxed residues highlight positions where a single majority residue or multiple chemically similar residues are found. Such residues are highlighted in red. WEEV contact residues on PCDH10 EC1 are indicated as shown in the legend. Domains of PCDH10 are indicated above the sequences.

10 Figure S10. Cryo-EM reconstruction of WEEV McMillan VLP in complex with VLDLR_LBD_-Fc, related to Figure 3.
(A) Workflow used for cryo-EM data processing of WEEV VLPs bound to VLDLR_LBD_-Fc.
(B) 3D representation of the angular distribution of particles and local resolution map estimates generated using Relion of WEEV McMillan VLP in complex with VLDLR_LBD_-Fc.
(C) Fourier shell correlation curves for WEEV McMillan VLP in complex with VLDLR_LBD_-Fc. The threshold used to estimate the resolution is 0.143. See Methods for additional details.
(D) Fitting of VLDLR LA2 into the LA repeat density of site 2. An elongated side chain density that could only be explained by an arginine (present in LA2) but not a serine (present in LA3) allowed for unambiguous identification of LA2 as the bound LA repeat at this site.
(E) Comparison of WEEV McMillan E2 and CBA87 E2 near the key polymorphic E2 residues at positions 181 and 214. E2 E181 in CBA87 makes a salt bridge with E2 R214 (left panel). The Q214R substitution in McMillan E2 may locally destabilize E2 by positioning three basic residues (K181, K190, and R214) in proximity (right panel).

11 Figure S11. Sequence alignments of WEEV and HJV E2 glycoproteins, related to Figures 5 and 6.
The strain information and accession numbers are as follows: WEEV California (GenBank: KJ554965.1), WEEV Fleming (GenBank: MN477208.1), WEEV McMillan (GenBank: DQ393792.1), WEEV Y62–33 (GenBank: KT844544.1), WEEV CU71-CPA (GenBank: KT844545.1), WEEV BFS932 (GenBank: KJ554966.1), WEEV BFS2005 (GenBank: GQ287644.1), WEEV BFS09997 (GenBank: KJ554974.1), WEEV EP6 (GenBank: KJ554967.1), WEEV Montana-64 (GenBank: GQ287643.1), WEEV 71V1659 (GenBank: NP_640331.1), WEEV 85–452NM (GenBank: GQ287647.1), WEEV PV012357A (GenBank: KJ554987.1), WEEV R0PV00348A (GenBank: KJ554991.1), WEEV R02PV003422B (GenBank: KJ554990.1), WEEV Imperial 181 (GenBank: GQ287641.1), WEEV CBA87 (GenBank: DQ432026.1), WEEV EQ1090 (GenBank: PP544260.1), WEEV DIAVE218 (GenBank: PP620644.1), WEEV Ar Enc MV (GenBank: KT844542), WEEV TR25717 (GenBank: KT844541), WEEV AG80–646 (GenBank: NC_075015), Highlands J virus 585–01 (GenBank: NC_012561.1). The red background denotes residues that are completely conserved in all sequences. Boxed residues highlight positions where a single majority residue or multiple chemically similar residues are found. Such residues are highlighted in red. Receptors contact residues and N-linked glycan sites are indicated as shown in the legend. E2 domains are indicated above the sequence alignment.

12 Figure S12. Sequence alignments of WEEV and HJV E1 glycoproteins, related to Figures 5 and 6.
The strain information and accession numbers are as follows: WEEV California (GenBank: KJ554965.1), WEEV Fleming (GenBank: MN477208.1), WEEV McMillan (GenBank: DQ393792.1), WEEV Y62–33 (GenBank: KT844544.1), WEEV CU71-CPA (GenBank: KT844545.1), WEEV BFS932 (GenBank: KJ554966.1), WEEV BFS2005 (GenBank: GQ287644.1), WEEV BFS09997 (GenBank: KJ554974.1), WEEV EP6 (GenBank: KJ554967.1), WEEV Montana-64 (GenBank: GQ287643.1), WEEV 71V1659 (GenBank: NP_640331.1), WEEV 85–452NM (GenBank: GQ287647.1), WEEV PV012357A (GenBank: KJ554987.1), WEEV R0PV00348A (GenBank: KJ554991.1), WEEV R02PV003422B (GenBank: KJ554990.1), WEEV Imperial 181 (GenBank: GQ287641.1), WEEV CBA87 (GenBank: DQ432026.1), WEEV EQ1090 (GenBank: PP544260.1), WEEV DIAVE218 (GenBank: PP620644.1), WEEV Ar Enc MV (GenBank: KT844542), WEEV TR25717 (GenBank: KT844541), WEEV AG80–646 (GenBank: NC_075015), Highlands J virus 585–01 (GenBank: NC_012561.1). The red background highlights residues completely conserved in all sequences aligned. Boxed residues highlight positions where a single majority residue or multiple chemically similar residues could be identified. Such residues are highlighted in red. Receptors contact residues and N-linked glycan sites are indicated as shown in the legend. E1 domains are indicated above the sequence alignment.

13 Table S1. Strain information for viral sequences used for RVP production or phylogenetic analyses, related to Figures 1, 6 and S1.
Table S2. Cryo-EM data collection and validation statistics, related to Figures 1, 2 and 3.

## Figures and Tables

**Table:** Key resources table

REAGENT or RESOURCE	SOURCE	IDENTIFIER
Antibodies		
Anti-PCDH10 antibody	Proteintech	Cat#: 21859-1-AP; RRID: AB_2878929
Anti-VLDLR antibody	GeneTex	Cat#: GTX79552; RRID: AB_11171827
Anti-MXRA8 antibody	MBL International	Cat#: W040-3; RRID: AB_2801291
Rabbit IgG isotype	Proteintech	Cat#: 30000-0-AP; RRID: AB_2819035
Mouse IgG isotype	BD Biosciences	Cat#: BDB557351; RRID: AB_10050406
PE-conjugated donkey anti-rabbit F(ab’)2 fragment	Jackson ImmunoResearch	Cat#: 711-116-152; RRID: AB_2340599
PE-conjugated donkey anti-mouse F(ab’)2 fragment	Jackson ImmunoResearch	Cat#: 715-116-150; RRID: AB_2340798
APC-conjugated rat anti-DYKDDDDK (Flag) antibody	BioLegend	Cat#: 637307; RRID: AB_2561496
APC-conjugated rat isotype control antibody	BioLegend	Cat#: 402306; RRID: AB_3097097
Anti-Human IgG (Fc specific)-Peroxidase antibody	Sigma	Cat#: A0170; RRID: AB_257868
Anti-WEEV E2-E1 monoclonal antibody SKW11	Sutton et al.^59^	N/A
		
Bacterial and virus strains		
Highlands J virus (strain B 230)	ATCC	Cat#: VR-622
		
Chemicals, peptides, and recombinant proteins		
DMEM	Gibco	Cat#: C11995500BT
Expi293^™^ Expression Medium	Thermo Fisher Scientific	Cat#: A1435101
Neurobasal medium	Thermo Fisher Scientific	Cat#: 21103
Glutamax	Thermo Fisher Scientific	Cat#: 35050
B-27	Thermo Fisher Scientific	Cat#: 17504
Y-27632	Stemcell Technologies	Cat#: 72302
Fetal bovine serum (FBS)	ATLAS Biologicals	Cat#: F-0500-D
HEPES	Thermo Fisher Scientific	Cat#: 15630080
RPMI1640	Thermo Fisher Scientific	Cat#: 11835055
Pen strep (penicillin-streptomycin)	Gibco	Cat#: 10378016
Opti-MEM	Thermo Fisher Scientific	Cat#: 31985088
Dulbecco’s Phosphate Buffered Saline (DPBS)	Thermo Fisher Scientific	Cat#: 14190-144
Pierce Chromogenic Endotoxin Quantification Kit	Thermo Fisher Scientific	Cat# A39552
MabSelectTM PrismA protein A affinity resin	Cytiva	Cat#: 17549801
Superdex 200 increase 10/300 GL column	Cytiva	Cat#: 28990944
Human PCDH10_EC1_-Fc	Li et al.^15^	N/A
Human VLDLR_LBD_-Fc	Clark et al.^34^	N/A
Human VLDLR LA(1-2)-Fc	This study	N/A
Sparrow PCDH10_EC1_-Fc	This study	N/A
Human MXRA8_ect_-Fc	Clark et al.^34^	N/A
Human PCDH10_EC1_-twin-strep	This study	N/A
Receptor associated protein (RAP)	Clark et al.^34^	N/A
		
Critical commercial assays		
ExpiFectamineTM 293 Transfection Kit	Thermo Fisher Scientific	Cat#: A14525
Lipofectamine 3000	Thermo Fisher Scientific	Cat#: L3000150
e-Myco PCR detection kit	Bulldog Bio	Cat#: 25234
		
Deposited data		
WEEV CBA87 VLP density map	This study	EMD-47117
WEEV CBA87 VLP model	This study	PDB ID: 9DQX
WEEV CBA87 VLP/human PCDH10_EC1_-Fc density map	This study	EMD-47116
WEEV CBA87 VLP/human PCDH10_EC1_-Fc model	This study	PDB ID: 9DQV
WEEV Imperial 181 VLP/sparrow PCDH10_EC1_-Fc density map	This study	EMD-47118
WEEV Imperial 181 VLP/sparrow PCDH10_EC1_-Fc model	This study	PDB ID: 9DQY
WEEV McMillan VLP/VLDLR_LBD_-Fc density map	This study	EMD-47119
WEEV McMillan VLP/ VLDLR_LBD_-Fc model	This study	PDB ID: 9DQZ
		
Experimental models: Cell lines		
Expi293F^™^ cells	Thermo Fisher Scientific	Cat#: A14527, RRID: CVCL_D615
HEK 293T (human kidney epithelial)	ATCC	Cat#: CRL-11268, RRID:CVCL_0063
Vero E6 (*Cercopithecus aethiops* kidney epithelial)	ATCC	Cat#: CRL-1586, RRID: CVCL_0574
K562 (human chronic myelogenous leukemia)	ATCC	Cat#: CCL-243, RRID:CVCL_0004
Embryonic day 17 mouse cortical neurons	Thermo Fisher Scientific	Cat#: A15586
K562 cells expressing *P. domesticus* PCDH10	Li et al.^15^	N/A
K562 cells expressing *E. caballus* PCDH10	Li et al.^15^	N/A
K562 cells expressing *P. domesticus* MXRA8	This study	N/A
K562 cells expressing *M. musculus* PCDH10	Li et al.^15^	N/A
K562 cells expressing *T. sirtalis* PCDH10	Li et al.^15^	N/A
		
Experimental models: Organisms/strains		
CD-1 IGS mice	Charles River	Strain Code 022
		
Recombinant DNA		
pVRC-human PCDH10_EC1_-Fc	Li et al.^15^	N/A
pVRC-sparrow PCDH10_EC1_-Fc	This study	N/A
pVRC-human VLDLR_LBD_-Fc	Clark et al.^34^	N/A
pVRC-human MXRA8_ect_-Fc	Clark et al.^34^	N/A
pVRC-human PCDH10_EC1_-twin-strep	This study	N/A
pCAGGS-human RAP	Clark et al.^34^	N/A
pCAGGS-WEEV 71V1658 E3-E2-6K/TF-E1 (and mutants)	Li et al.^15^ and this study	N/A
pCAGGS-WEEV Fleming E3-E2-6K/TF-E1 (and mutants)	Li et al.^15^ and this study	N/A
pCAGGS-WEEV McMillan E3-E2-6K/TF-E1 (and mutants)	Li et al.^15^ and this study	N/A
pCAGGS-WEEV Imperial 181 E3-E2-6K/TF-E1 (and mutants)	Li et al.^15^ and this study	N/A
pCAGGS-WEEV CBA87 E3-E2-6K/TF-E1	Li et al.^15^	N/A
pCAGGS-WEEV BFS09997 E3-E2-6K/TF-E1	This study	N/A
pCAGGS-WEEV EP6 E3-E2-6K/TF-E1	This study	N/A
pCAGGS-WEEV AG80-646 E3-E2-6K/TF-E1	This study	N/A
pCAGGS-WEEV TR25717 E3-E2-6K/TF-E1	This study	N/A
pCAGGS-WEEV EQ1090 E3-E2-6K/TF-E1	This study	N/A
pCAGGS-WEEV DILAVE218 E3-E2-6K/TF-E1	This study	N/A
pCAGGS-HJV 585-01 E3-E2-6K/TF-E1 (and mutants)	This study	N/A
lentiGuide-Puro-human PCDH10	Li et al.^15^	N/A
lentiGuide-Puro-human PCDH10 stalk-Flag	Li et al.^15^	N/A
lentiGuide-Puro-human PCDH10 EC1-Flag (and mutants)	Li et al.^15^	N/A
lentiGuide-Puro-human VLDLR	Clark et al.^34^	N/A
lentiGuide-Puro-human VLDLR ΔLBD-Flag	Clark et al.^34^	N/A
lentiGuide-Puro-human VLDLR Flag-tagged single LA repeat	Yang et al.^24^	N/A
lentiGuide-Puro-human MXRA8	Clark et al.^34^	N/A
lentiGuide-Puro-human ApoER2 iso2	Clark et al.^34^	N/A
lentiGuide-Puro-sparrow PCDH10	Li et al.^15^	N/A
lentiGuide-Puro-sparrow MXRA8	This study	N/A
lentiGuide-Puro-garter snake PCDH10	Li et al.^15^	N/A
lentiGuide-Puro-mouse PCDH10	Li et al.^15^	N/A
lentiGuide-Puro horse PCDH10	Li et al.^15^	N/A
WEEV CBA87 virus-like particle in pVRC plasmid	Ko et al.^32^	N/A
WEEV McMillan virus-like particle in pVRC plasmid	Li et al.^15^	N/A
WEEV Imperial 181 virus-like particle in pVRC plasmid	This study	N/A
Western equine encephalitis virus (strain McMillan) molecular clone	Gardner et al.^60^	
		
Software and algorithms		
RELION v3.1.4	Zivanov et al.^61^	N/A
crYOLO v1.8.2	Wagner et al.^62^	N/A
DeepEMhancer v20210511	Sanchez-Garcia et al.^63^	N/A
MotionCor2 v1.6.4	Zheng et al.^64^	N/A
CTFFIND-4.1 v4.1.14	Rohou et al.^65^	N/A
UCSF ChimeraX v1.6.1	Pettersen et al.^66^	N/A
PyMOL v3.0.2	(https://www.pymol.org/pymol)	N/A
*Coot* v0.9.8	Emsley et al.^67^	N/A
Phenix v1.21rc1-5127	Adams et al.^68^	N/A
AlphaFold2	Jumper et al.^69^	N/A
GraphPad Prism v10.2.3	GraphPad	N/A
FlowJo v10.6.2	FlowJo, LLC	N/A
Incucyte S3 Software v2023B	Sartorius	N/A
MEGA v11.0.10	MEGA Development Team	N/A
		
Other		
Quantifoil grids R 0.6/1 300 mesh, gold	Electron Microscopy Sciences	Cat#: Q350AR-06
Quantifoil grids R 2/2 300 mesh, gold	Electron Microscopy Sciences	Cat#: Q3100AR2
		
