# Supplementary material for: Molecular basis for shifted receptor recognition by an encephalitic arbovirus
Source: Cell. Author manuscript; Available in PMC 2025 Sep 3. (PMC12406711; doi:10.1016/j.cell.2025.03.029)
Supplement: 1 — Figure S1. Phylogenetic tree of WEEV strains and Highlands J virus, related to Figure 1. Maximum likelihood phylogenetic tree of 57 WEEV strains using the coding sequences of the structural polyprotein genes. Highlands J virus strain 64A-1519 (GenBank: KT429021) is also included. Scale bar represents 0.05 nucleotide substitutions per site. SFV strain SFV4, VEEV strain TC-83, and Madariaga virus (MADV) strain 267113 were included in the phylogenetic analysis (not shown). Numbers at nodes indicate bootstrap values. In cases in which the branches are too small, bootstrap values may not be shown. The three lineages (A, B, and C) and B sublineages (B1, B2, and B3) are indicated. Taxon labels include strain name and year of isolation. GenBank accession numbers are provided in Table S1. [file NIHMS2067620-supplement-1.pdf]

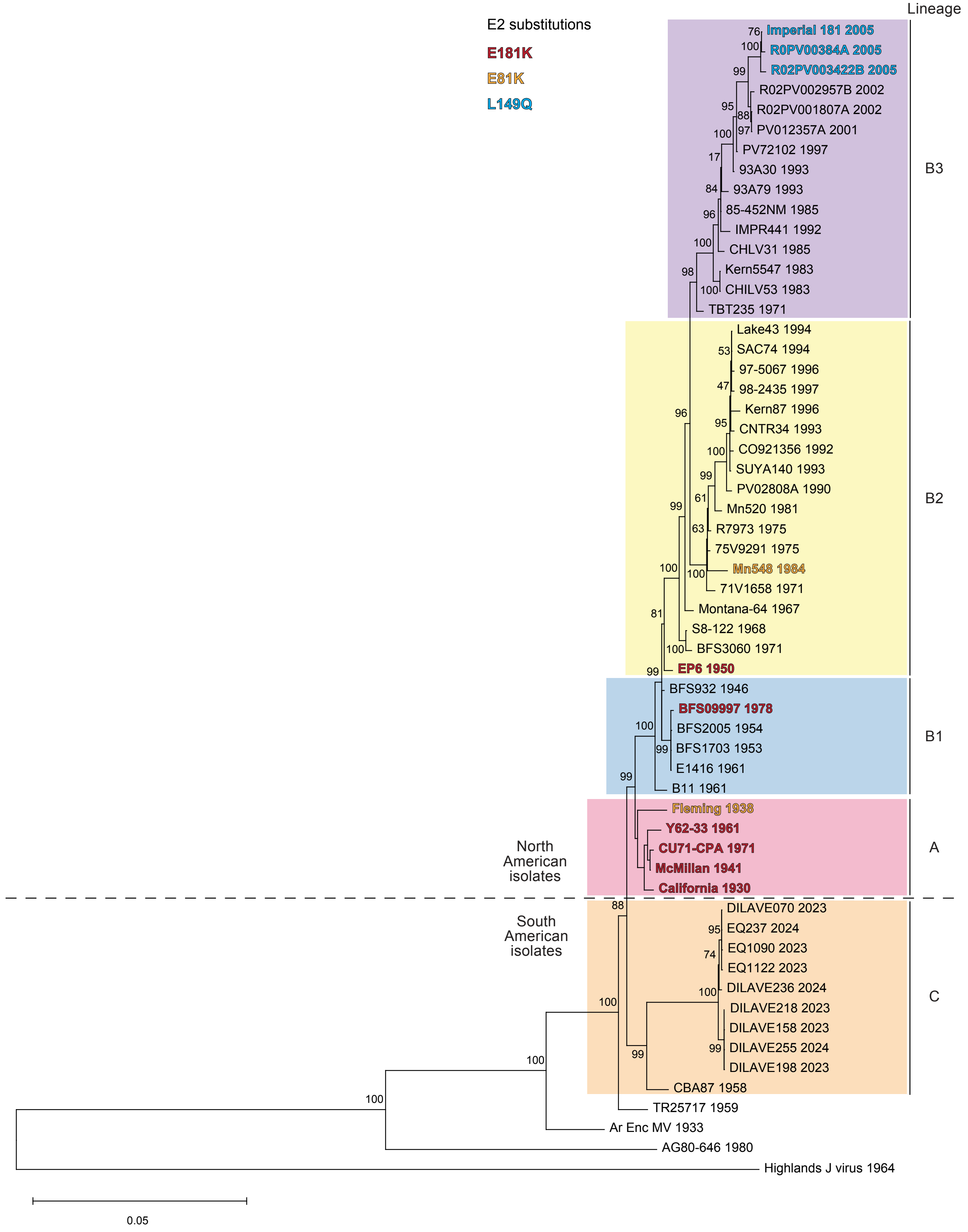

Lineage
E2 substitutions
E181K
E81K
L149Q
Imperial 181 2005
R0PV00384A 2005
R02PV003422B 2005
R02PV002957B 2002
R02PV001807A 2002
PV012357A 2001
PV72102 1997
93A30 1993
93A79 1993
85-452NM 1985
IMPR441 1992
CHLV31 1985
Kern5547 1983
CHILV53 1983
TBT235 1971
B3
Lake43 1994
SAC74 1994
97-5067 1996
98-2435 1997
Kern87 1996
CNTR34 1993
CO921356 1992
SUYA140 1993
PV02808A 1990
Mn520 1981
R7973 1975
75V9291 1975
Mn548 1984
71V1658 1971
Montana-64 1967
S8-122 1968
BFS3060 1971
EP6 1950
B2
BFS932 1946
BFS09997 1978
BFS2005 1954
BFS1703 1953
E1416 1961
B11 1961
B1
Fleming 1938
Y62-33 1961
CU71-CPA 1971
McMillan 1941
California 1930
A
North American isolates
South American isolates
DILAVE070 2023
EQ237 2024
EQ1090 2023
EQ1122 2023
DILAVE236 2024
DILAVE218 2023
DILAVE158 2023
DILAVE255 2024
DILAVE198 2023
CBA87 1958
C
TR25717 1959
Ar Enc MV 1933
AG80-646 1980
Highlands J virus 1964
0.05
