# Supplementary figures and images for: Molecular basis for shifted receptor recognition by an encephalitic arbovirus
Source: Cell. Author manuscript; Available in PMC 2025 Sep 3. (PMC12406711; doi:10.1016/j.cell.2025.03.029)

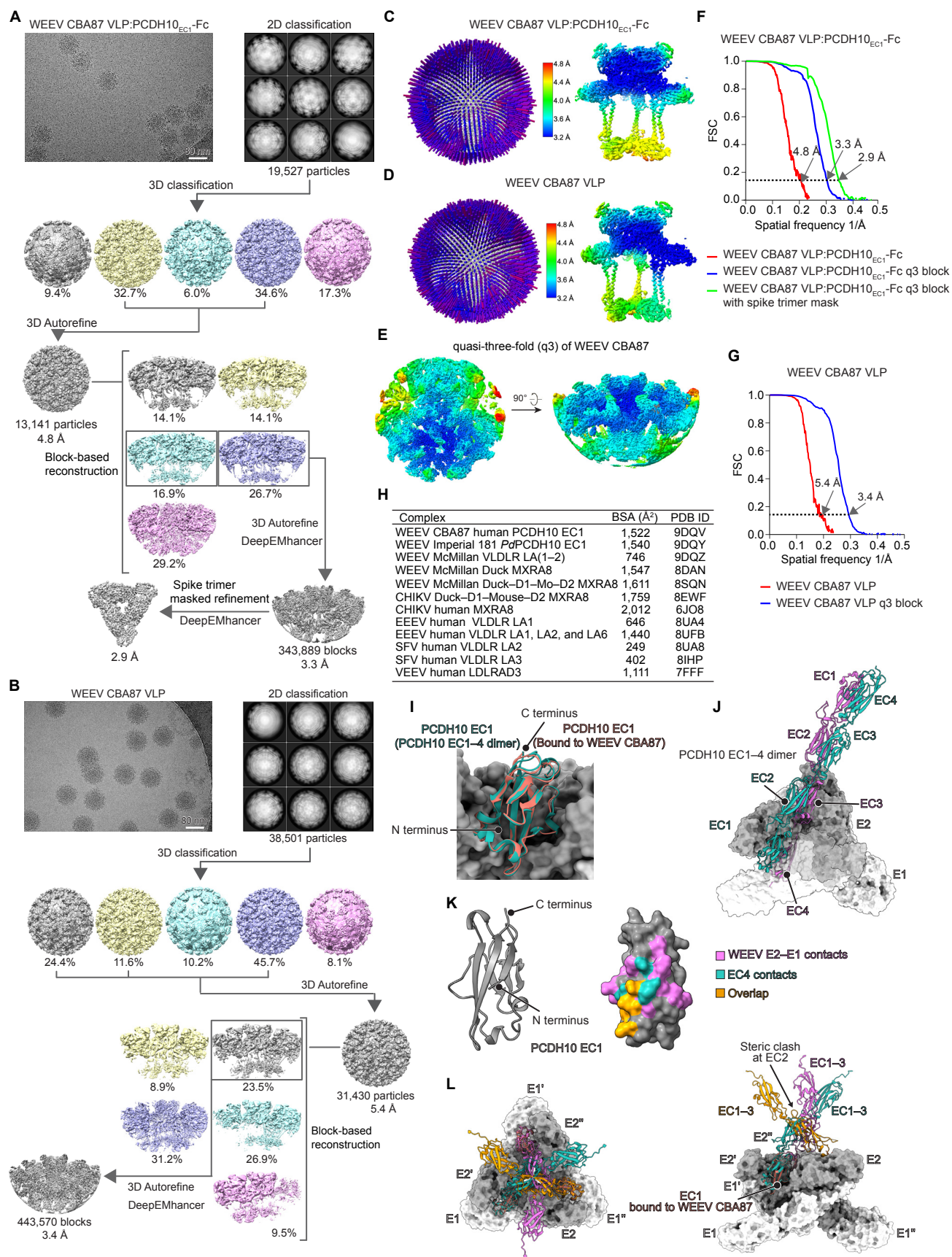

Supplement: 3 — Figure S3. Cryo-EM reconstructions of WEEV CBA87 VLPs alone or bound to human PCDH10EC1-Fc, related to Figure 1. (A) Workflow used for cryo-EM data processing of WEEV CBA87 VLPs bound to human PCDH10EC1-Fc. (B) Workflow used for cryo-EM data processing of unliganded WEEV CBA87 VLPs. (C and D) 3D representation of angular distribution of particles and local resolution map estimates using Relion of the WEEV CBA87 with PCDH10EC1-Fc complex (C) or WEEV CBA87 VLP alone (D). (E) Top and side views of a representative q3 block of the WEEV CBA87 VLPs, displayed on the local resolution map. (F and G) Fourier shell correlation curves of WEEV CBA87 VLP bound to PCDH10EC1-Fc (F) or WEEV CBA87 VLP alone (G) are shown. The threshold used to estimate the resolution is 0.143. (H) Buried surface area (BSA) calculations for alphavirus E2–E1 receptor complexes described here and in prior publications22–25,27,43. (I and J) Structural superposition of the X-ray crystal structure of PCDH10 EC1–4 homodimer (PDB ID: 6VFW)19 with the WEEV CBA87:PCDH10EC1-Fc complex. Panel (I) shows that there is no conformational change in PCDH10 EC1 when comparing the WEEV E2–E1 bound EC1 structure to EC1 within the EC1–EC4 homodimer. (J) Highlights steric clashes between one copy of EC1–EC4 in the EC1–EC4 homodimer and WEEV E2–E1. (K) Human PCDH10 EC1 in ribbon rendering (left) or surface rendering (right) in the same orientation. Contact residues for WEEV E2–E1 and for EC4 in an antiparallel PCDH10 homodimer are shown in magenta and cyan respectively. Overlapping contact residues are shown in orange. (L) Superposition of the X-ray crystal structure of PCDH10 EC1–6 (PDB ID: 6VG4)19 onto the EC1 protomers bound to the E2–E1 trimer of WEEV CBA87. Occupancy of the three receptor-binding sites on the trimer would result in steric hindrance between the EC2 repeats of neighboring receptor molecules. The left panel shows a top view, and the right panel shows a side view. [file NIHMS2067620-supplement-3.pdf]

**A**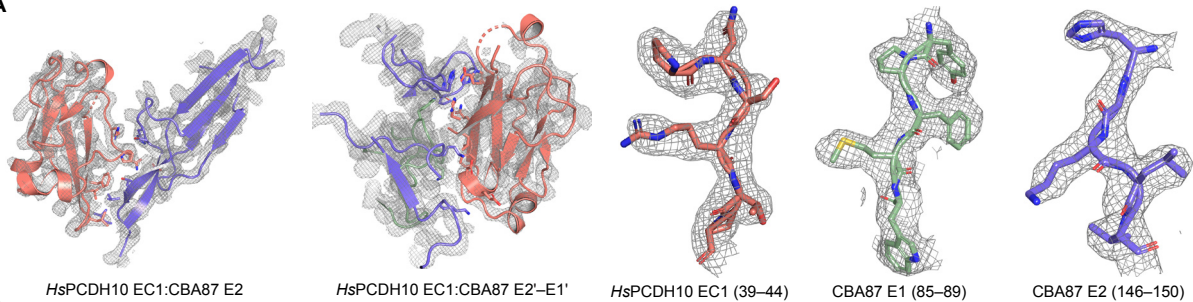**B**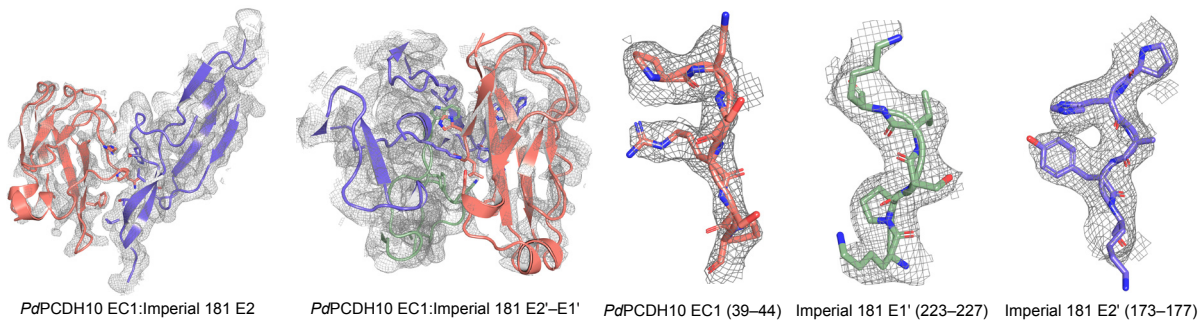**C**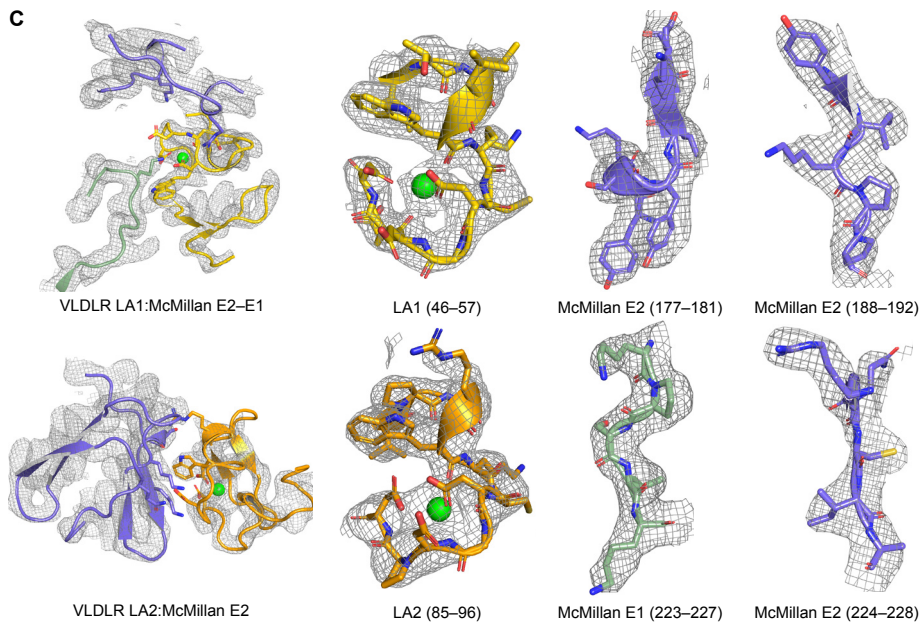**D**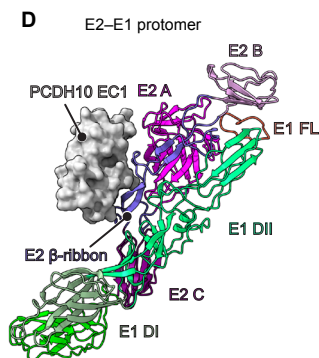**E**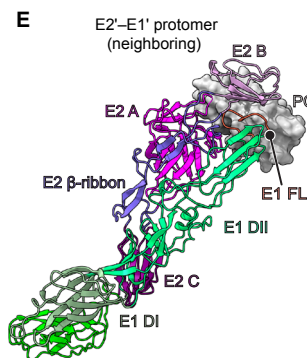**F**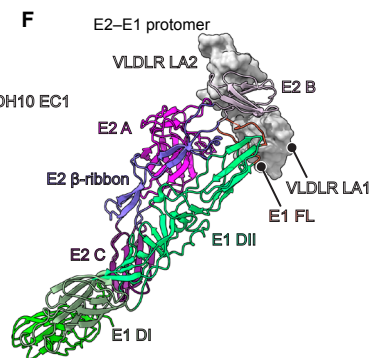

Supplement: 4 — Figure S4. Representative cryo-EM density maps of WEEV E2–E1 bound to alternate receptors and domains involved in receptor binding, related to Figures 1, 2 and 3. (A–C) Density maps of the indicated polypeptide segments from the following complexes: WEEV CBA87 VLP bound to HsPCDH10EC1-Fc (A), WEEV Imperial 181 VLP bound to PdPCDH10EC1-Fc (B), or WEEV McMillan VLP bound to VLDLRLBD-Fc (C). (D–F) Ribbon diagrams of WEEV E2–E1 heterodimers bound to surface-rendered PCDH10 EC1 or VLDLR LA(1–2). E2 domains (domains A, B, and C) and E1 domains (DI–III) are indicated in different colors. FL: fusion loop. [file NIHMS2067620-supplement-4.pdf]

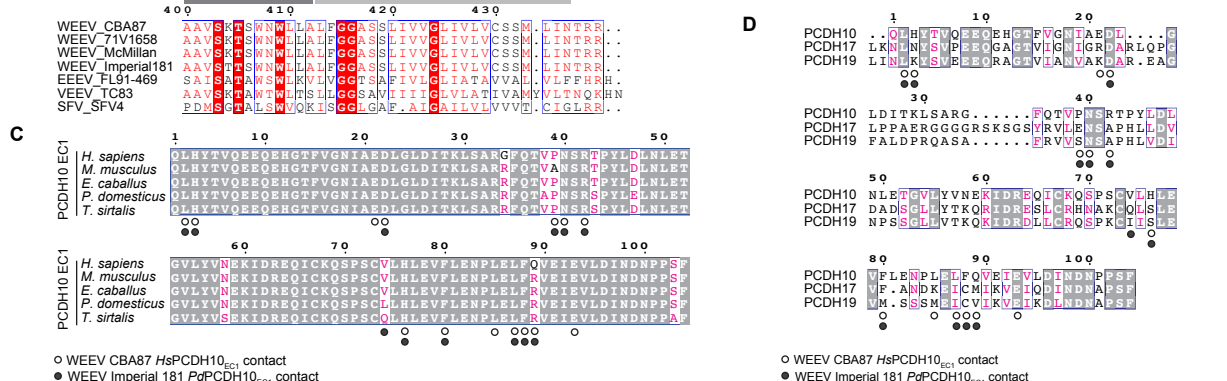

Supplement: 5 — Figure S5. Sequence alignments of alphavirus E2–E1 glycoproteins and protocadherin EC1 repeats, related to Figures 1, 2 and 7. (A and B) Sequence alignments of the E2 (A) and E1 (B) glycoproteins of WEEV CBA87 (GenBank: DQ432026.1), WEEV 71V658 (GenBank: NP_640331.1), WEEV McMillan (GenBank: DQ393792.1), Imperial 181 (GenBank: GQ287641), EEEV FL91–469 (GenBank: Q4QXJ7.1), VEEV TC83 (GenBank: AAB02517.1), and SFV4 (GenBank: AKC01668.1). E2 and E1 residues that are with 4 Å of the indicated receptors in the structures are shown as indicated in the legend, and essential basic residues contacting LA repeats based on prior structural studies of LA repeat bound alphavirus E2–E1 proteins22,24–27,40 are highlighted in green. Residues that are completely conserved in all aligned sequences have a red background. Boxed residues show positions where a single majority residue or multiple chemically similar residues are found. Such residues are in red. Domains of E2 and E1 are indicated above the sequences. (C) Sequence alignments of PCDH10 EC1 orthologs of H. sapiens PCDH10 (GenBank: NP_116586.1), M. musculus PCDH10 (GenBank: NP_001091642.1), E. caballus PCDH10 (GenBank: XP_023492316.1), P. domesticus PCDH10 (GenBank: XP_064272564.1), and T. sirtalis PCDH10 (GenBank: XP_013928164.1). (D) Sequence alignments of human PCDH10 EC1 with the EC1 repeats of other non-clustered δ2 protocadherins PCDH17 (GenBank: NP_001035519.1) and PCDH19 (GenBank: NP_001171809.1). Residues that are completely conserved have a gray background. Boxed residues highlight positions where a single majority residue or multiple chemically similar residues could be identified. Such residues are highlighted in pink. The panels were generated using ESPript 3.0.58 PCDH10 residues that are with 4 Å of the indicated E2 or E1 proteins in the structures are shown as indicated in the legend. [file NIHMS2067620-supplement-5.pdf]

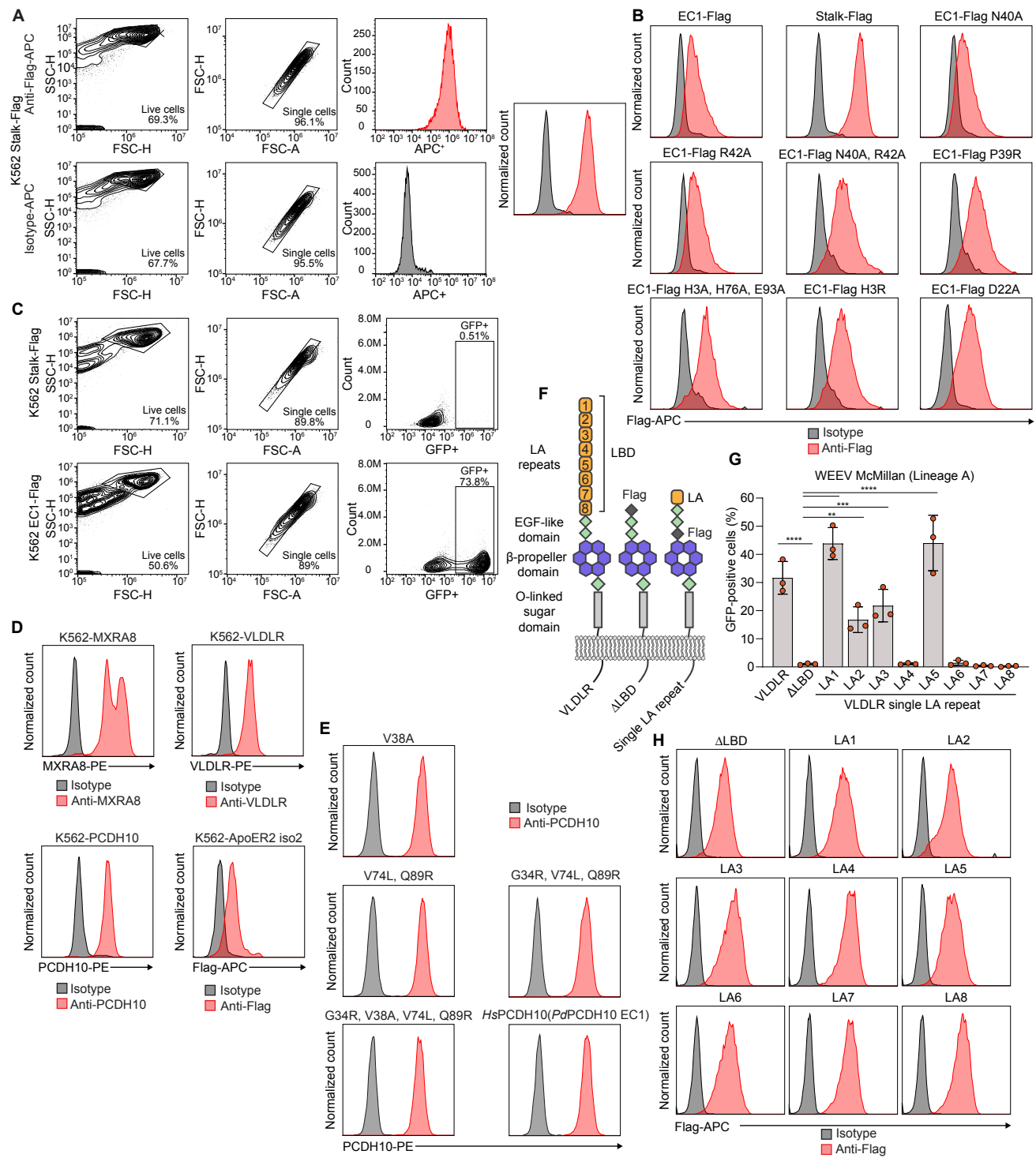

Supplement: 6 — Figure S6. Gating strategy, cell surface receptor staining, and WEEV McMillan VLDLR LA repeat dependencies, related to Figures 1, 2 and 3. (A) Example of flow cytometry gating strategy used to quantify cells stained by antibodies. The example shown is for K562 cells expressing the PCDH10 stalk-FLAG construct. (B) Cell surface immunostaining of K562 cell expressing PCDH10 truncation constructs by APC-conjugated anti-Flag or isotype control antibodies. (C) Example of flow cytometry gating strategy used to quantify cells expressing GFP following infection by GFP-expressing RVPs. The example provided is with WEEV 71V RVP. (D) Staining of K562 cells expressing the human orthologs of the indicated alphavirus receptors. PE: R-phycoerythrin. For staining of K562-ApoER2iso2, cells were incubated with RAP-Flag followed by allophycocyanin (APC)-conjugated anti-Flag or isotype control antibodies. (E) Staining of K562 cells expressing mutant human PCDH10 containing sparrow PCDH10 polymorphisms. (F) Schematic diagrams of WT VLDLR and single LA repeat constructs. A ΔLBD construct in which the entire LBD is replaced by an N-terminal Flag tag is used as a negative control in experiments. (G) Infection of K562 cells stably expressing VLDLR, ΔLBD-Flag, or single LA repeat constructs by WEEV McMillan RVPs. Infection was quantified by flow cytometry. Data are mean ± s.d. from three experiments performed in triplicates (n = 3). One-way ANOVA with Dunnett’s multiple comparisons test, ****P<0.0001; ***P<0.001; **P<0.01. (H) Cell surface immunostaining of K562 cells expressing VLDLR or Flag-tagged VLDLR truncation constructs by APC-conjugated anti-Flag or isotype control antibodies. [file NIHMS2067620-supplement-6.pdf]

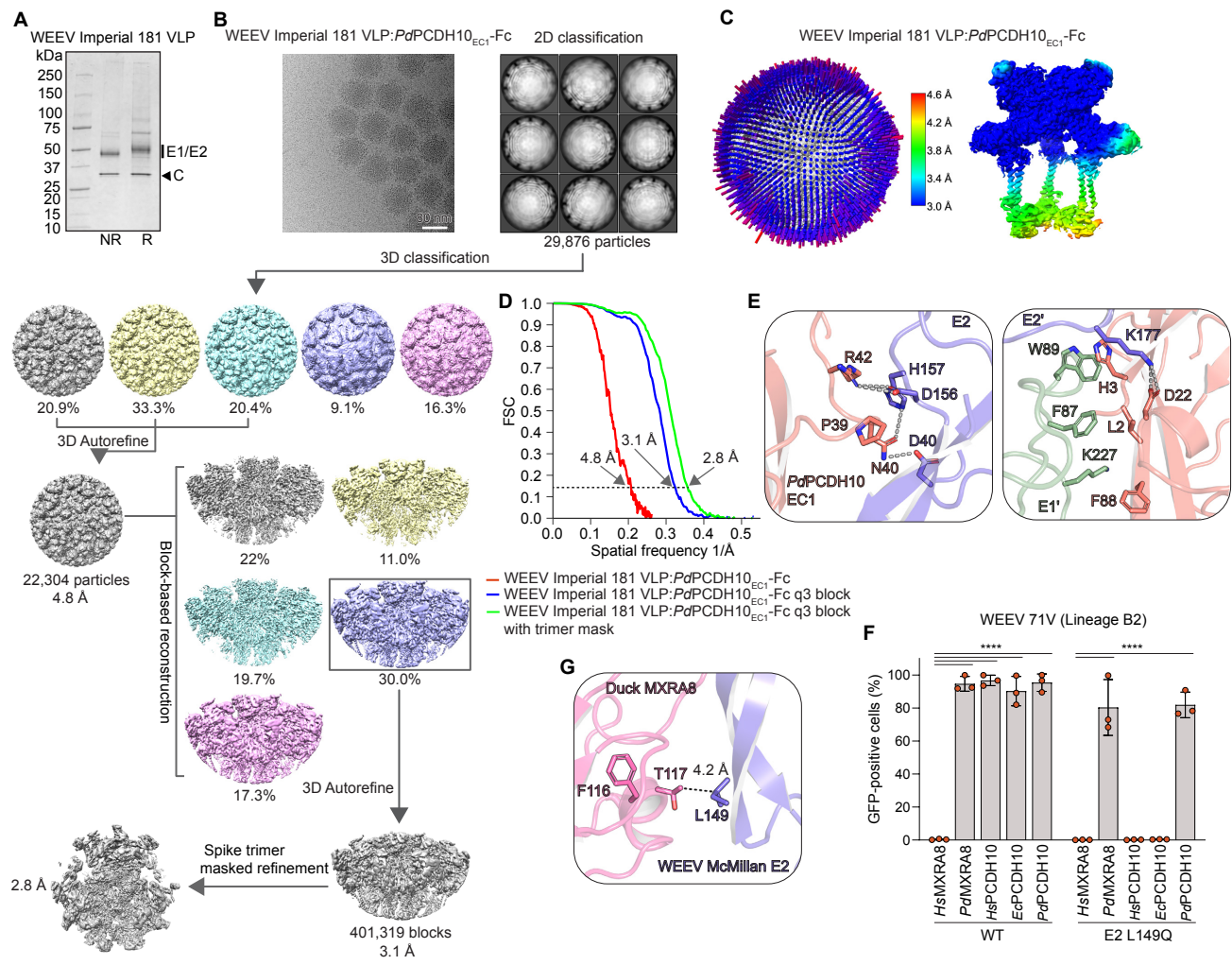

Supplement: 7 — Figure S7. Cryo-EM reconstruction of WEEV Imperial 181 VLPs in complex with PdPCDH10EC1-Fc, related to Figure 2. (A) Coomassie-stained SDS-PAGE gel of purified WEEV Imperial 181 VLPs. NR: non-reducing. R: reducing. (B) Workflow used for cryo-EM data processing of WEEV Imperial 181 VLPs bound to PdPCDH10EC1-Fc. (C) 3D representation of the angular distribution of particles and local resolution map estimates generated in Relion of WEEV Imperial 181 VLP in complex with PdPCDH10EC1-Fc. (D) Fourier shell correlation curves of WEEV Imperial 181 VLP in complex with PdPCDH10EC1Fc. The threshold used to estimate the resolution is 0.143. See Methods for additional details. (E) Interface between WEEV Imperial 181 E2–E1 or E2’–E1’ and PdPCDH10 EC1. Residues that participate in interactions between WEEV Imperial 181 E2–E1 heterodimers and PdPCDH10 EC1 are indicated, with polar contacts shown as gray dashed lines. (F) K562 cells stably expressing human (Hs), sparrow (Pd) PCDH10 or MXRA8, or horse (Ec) PCDH10 were infected with GFP-expressing WT or mutant 71V RVPs. Infection was measured by flow cytometry. Data are mean from three experiments performed in duplicates or triplicates (n = 3). Two-way ANOVA with Dunnett’s multiple comparisons test; ****P < 0.0001. (G) Interface between WEEV McMillan E2 and duck MXRA8 (PDB: 8DAN),25 showing E2 L149 and receptor residues in its vicinity. The closest distance between duck MXRA8 residue T117 and atoms on WEEV E2 L149 among the protomers is shown. The distance suggests that the L149Q substitution would be tolerated at the interface. [file NIHMS2067620-supplement-7.pdf]

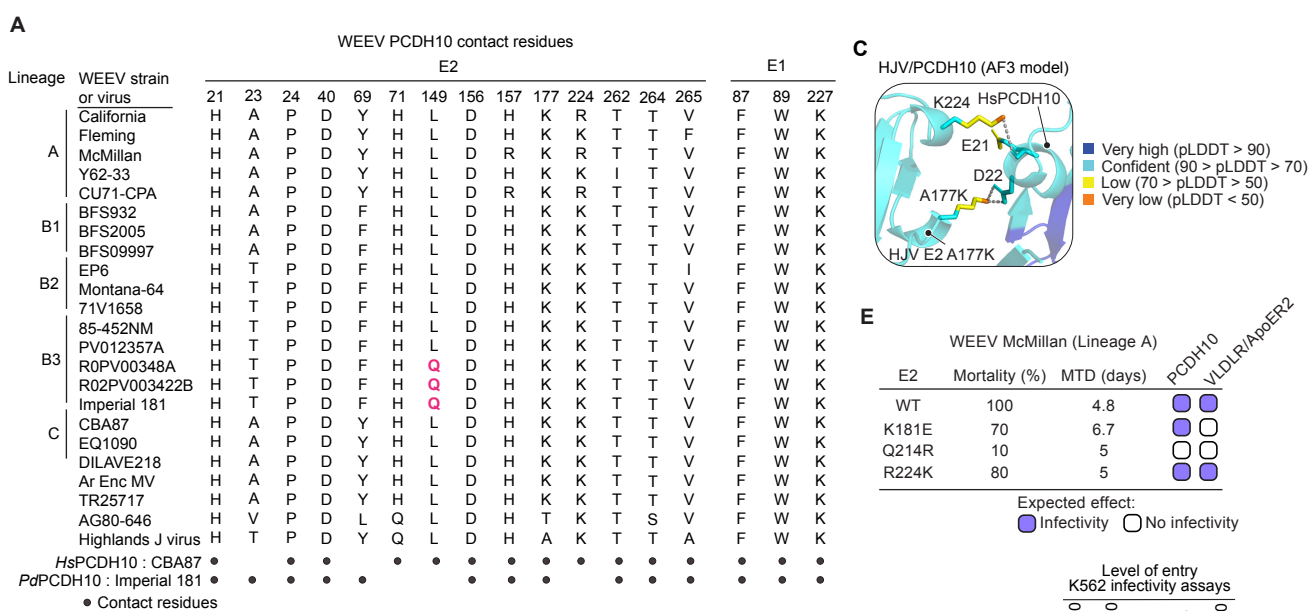

Supplement: 8 — Figure S8. Contact residues, AF3 modeling of HJV–PCDH10 interactions, and summary of mouse virulence of McMillan mutants and the effects of mutagenesis on receptor dependencies, related to Figures 4, 6 and 7. (A) WEEV E2 or E1 residues that contact PCDH10. WEEV CBA87 E2–E1 residues that contact HsPCDH10 or WEEV Imperial 181 E2–E1 residues that contact PdPCDH10 (< 4.0 Å) are indicated. A key E2 polymorphic residue (Q149) is colored pink. (B) WEEV E2 or E1 residues that contact VLDLR. Key polymorphic residues (E2 K181 and E2 K81) are colored pink. (C) pLDDT scores of the interaction interface for the AF3 model of HJV containing E2 A177K substitution in complex with human PCDH10 EC1. Atoms with very high confidence (pLDDT > 90) are shown in deep blue, confidence (90 > pLDDT > 70) in light blue, low confidence (70 > pLDDT > 50) in yellow, and very low confidence (pLDDT < 50) in orange. (D) Summary of effects of WEEV E2–E1 mutagenesis on receptor-mediated entry into K562 cells. K562 infectivity assays for WEEV WT and mutant RVPs conducted in the current study are summarized. “Entry”, RVP infection with 20–100% GFP-positive cells achieved and significantly higher than infection of K562 cells expressing the negative control human MXRA8 (P<0.05); “Weak entry”, RVP infection with 5–20% GFP-positive cells achieved and significantly high than infection of K562 cells expressing human MXRA8 (P<0.05); “No entry”, RVP infection levels that are not significantly different than infection levels on K562 cells expressing human MXRA8 (P>0.05). (E) Effects of WEEV E2 McMillan mutations on mortality and mean time-to-death (MTD) when five-week-old female CD1 mice were inoculated 1000 PFU subcutaneously in the left thigh, summarized from Mossel et al.41 The expected effects of E2 mutations on PCDH10 or VLDLR/ApoER2 are based on the results of K562 infectivity assays with McMillan RVPs shown in Figure 3G. [file NIHMS2067620-supplement-8.pdf]

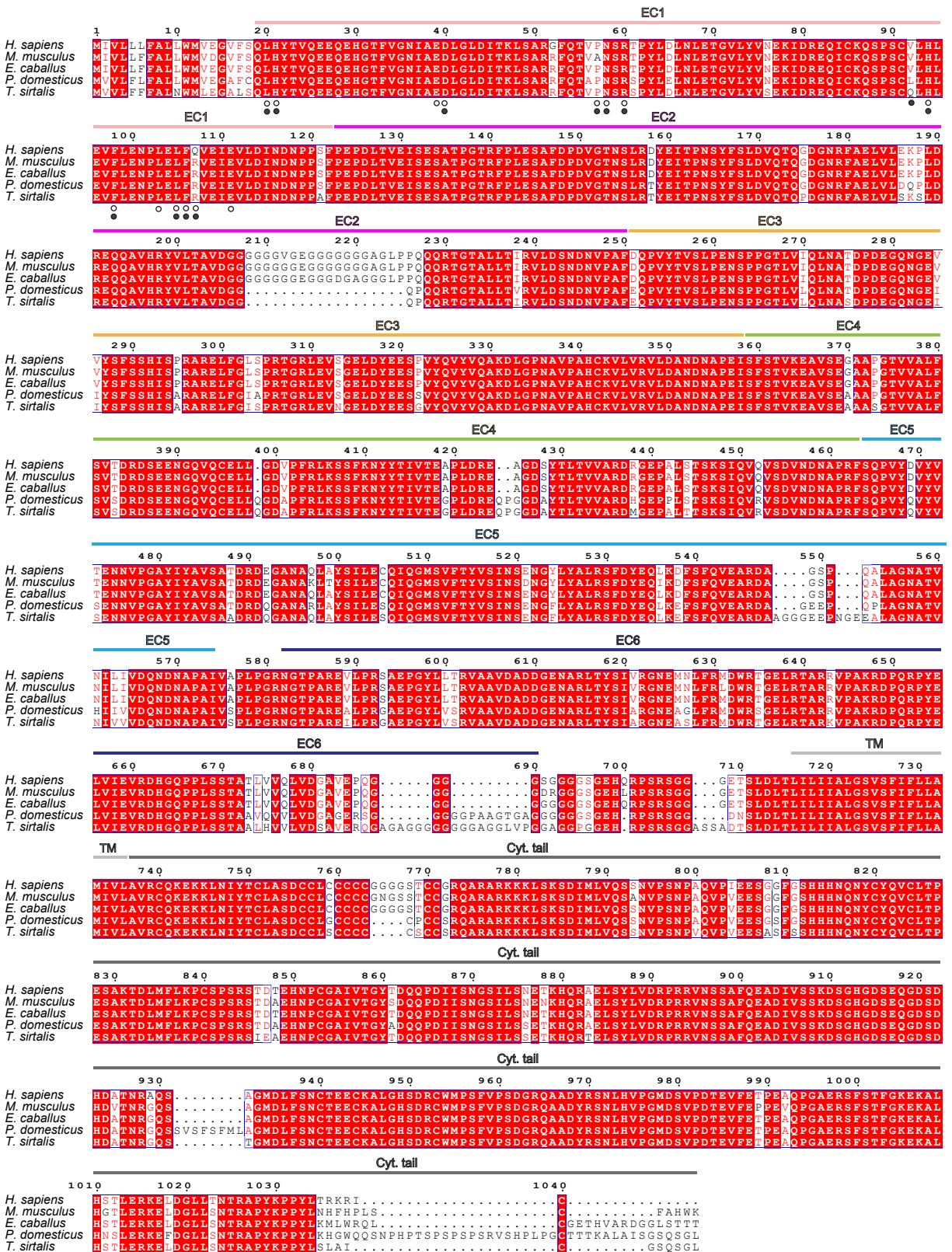

○ WEEV CBA87 *Hs*PCDH10<sub>EC1</sub> contact

● WEEV Imperial 181 *Pd*PCDH10<sub>EC1</sub> contact

Supplement: 9 — Figure S9. Sequence alignments of PCDH10 orthologs, related to Figure 2. Sequence alignments of PCDH10 orthologs of H. sapiens (GenBank: NP_116586.1); M. musculus (GenBank: NP_001091642.1); E. caballus (GenBank: XP_023492316.1); P. domesticus (GenBank: XP_064272564.1); T. sirtalis (GenBank: XP_013928164.1). Residues that are completely conserved have a red background. The red background denotes residues that are completely conserved in all sequences. Boxed residues highlight positions where a single majority residue or multiple chemically similar residues are found. Such residues are highlighted in red. WEEV contact residues on PCDH10 EC1 are indicated as shown in the legend. Domains of PCDH10 are indicated above the sequences. [file NIHMS2067620-supplement-9.pdf]

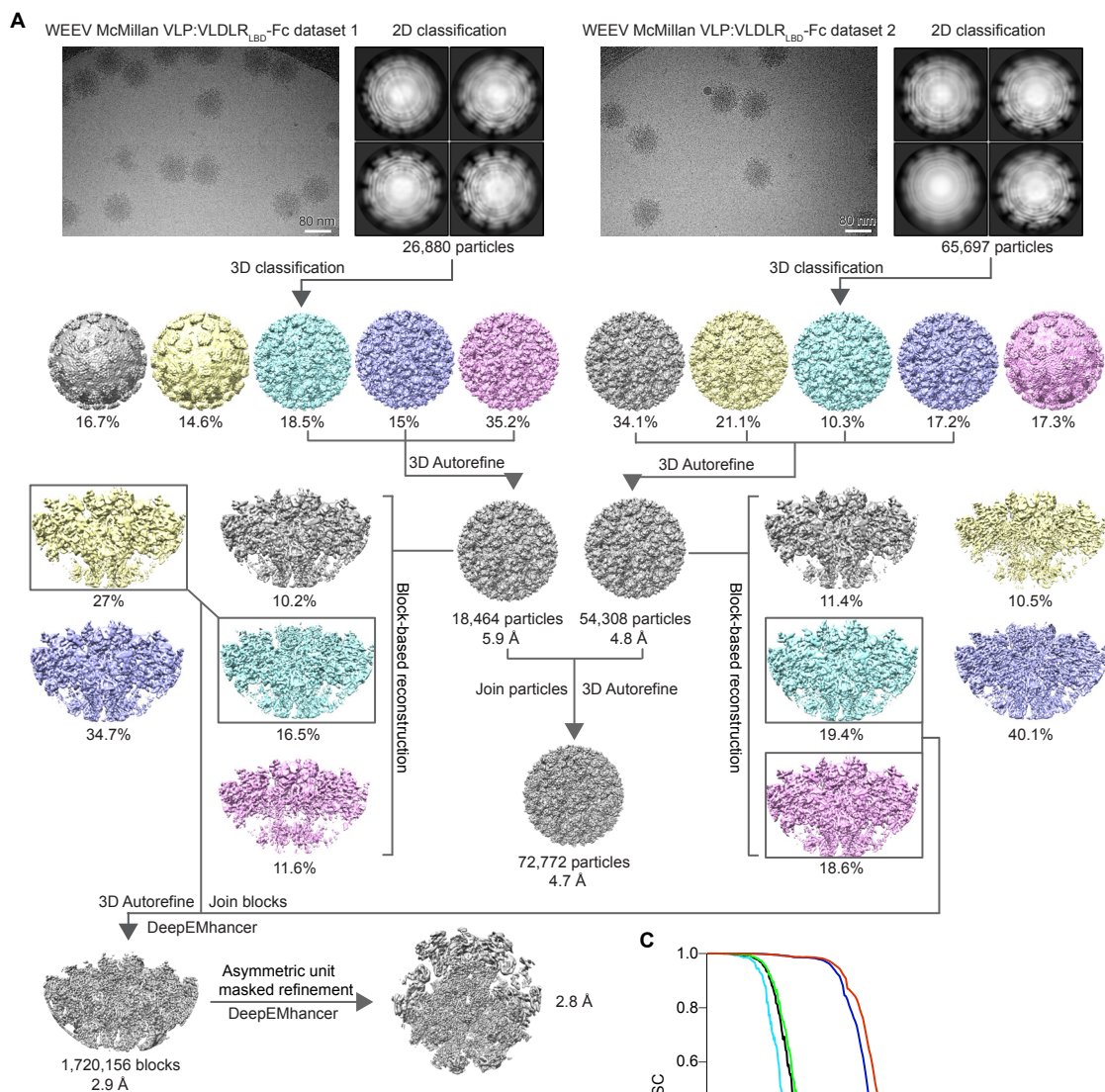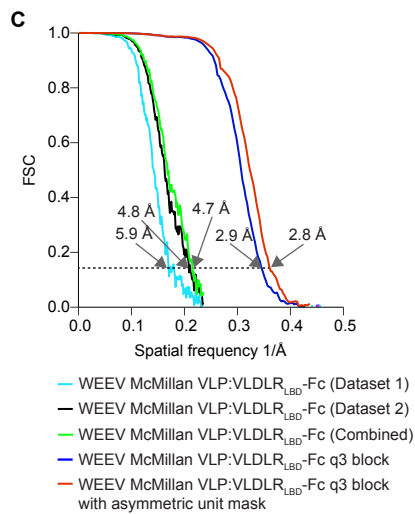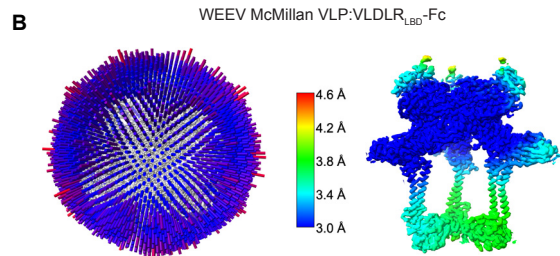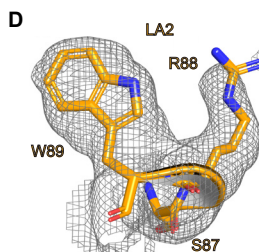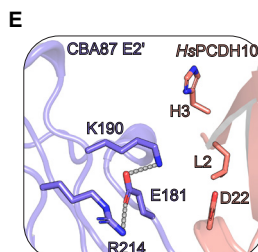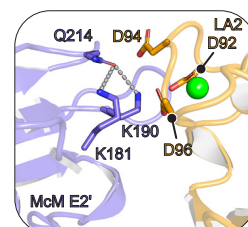

Supplement: 10 — Figure S10. Cryo-EM reconstruction of WEEV McMillan VLP in complex with VLDLRLBD-Fc, related to Figure 3. (A) Workflow used for cryo-EM data processing of WEEV VLPs bound to VLDLRLBD-Fc. (B) 3D representation of the angular distribution of particles and local resolution map estimates generated using Relion of WEEV McMillan VLP in complex with VLDLRLBD-Fc. (C) Fourier shell correlation curves for WEEV McMillan VLP in complex with VLDLRLBD-Fc. The threshold used to estimate the resolution is 0.143. See Methods for additional details. (D) Fitting of VLDLR LA2 into the LA repeat density of site 2. An elongated side chain density that could only be explained by an arginine (present in LA2) but not a serine (present in LA3) allowed for unambiguous identification of LA2 as the bound LA repeat at this site. (E) Comparison of WEEV McMillan E2 and CBA87 E2 near the key polymorphic E2 residues at positions 181 and 214. E2 E181 in CBA87 makes a salt bridge with E2 R214 (left panel). The Q214R substitution in McMillan E2 may locally destabilize E2 by positioning three basic residues (K181, K190, and R214) in proximity (right panel). [file NIHMS2067620-supplement-10.pdf]
