# Supplementary material for: Molecular basis for shifted receptor recognition by an encephalitic arbovirus
Source: Cell. Author manuscript; Available in PMC 2025 Sep 3. (PMC12406711; doi:10.1016/j.cell.2025.03.029)
Supplement: 11 — Figure S11. Sequence alignments of WEEV and HJV E2 glycoproteins, related to Figures 5 and 6. The strain information and accession numbers are as follows: WEEV California (GenBank: KJ554965.1), WEEV Fleming (GenBank: MN477208.1), WEEV McMillan (GenBank: DQ393792.1), WEEV Y62–33 (GenBank: KT844544.1), WEEV CU71-CPA (GenBank: KT844545.1), WEEV BFS932 (GenBank: KJ554966.1), WEEV BFS2005 (GenBank: GQ287644.1), WEEV BFS09997 (GenBank: KJ554974.1), WEEV EP6 (GenBank: KJ554967.1), WEEV Montana-64 (GenBank: GQ287643.1), WEEV 71V1659 (GenBank: NP_640331.1), WEEV 85–452NM (GenBank: GQ287647.1), WEEV PV012357A (GenBank: KJ554987.1), WEEV R0PV00348A (GenBank: KJ554991.1), WEEV R02PV003422B (GenBank: KJ554990.1), WEEV Imperial 181 (GenBank: GQ287641.1), WEEV CBA87 (GenBank: DQ432026.1), WEEV EQ1090 (GenBank: PP544260.1), WEEV DIAVE218 (GenBank: PP620644.1), WEEV Ar Enc MV (GenBank: KT844542), WEEV TR25717 (GenBank: KT844541), WEEV AG80–646 (GenBank: NC_075015), Highlands J virus 585–01 (GenBank: NC_012561.1). The red background denotes residues that are completely conserved in all sequences. Boxed residues highlight positions where a single majority residue or multiple chemically similar residues are found. Such residues are highlighted in red. Receptors contact residues and N-linked glycan sites are indicated as shown in the legend. E2 domains are indicated above the sequence alignment. [file NIHMS2067620-supplement-11.pdf]

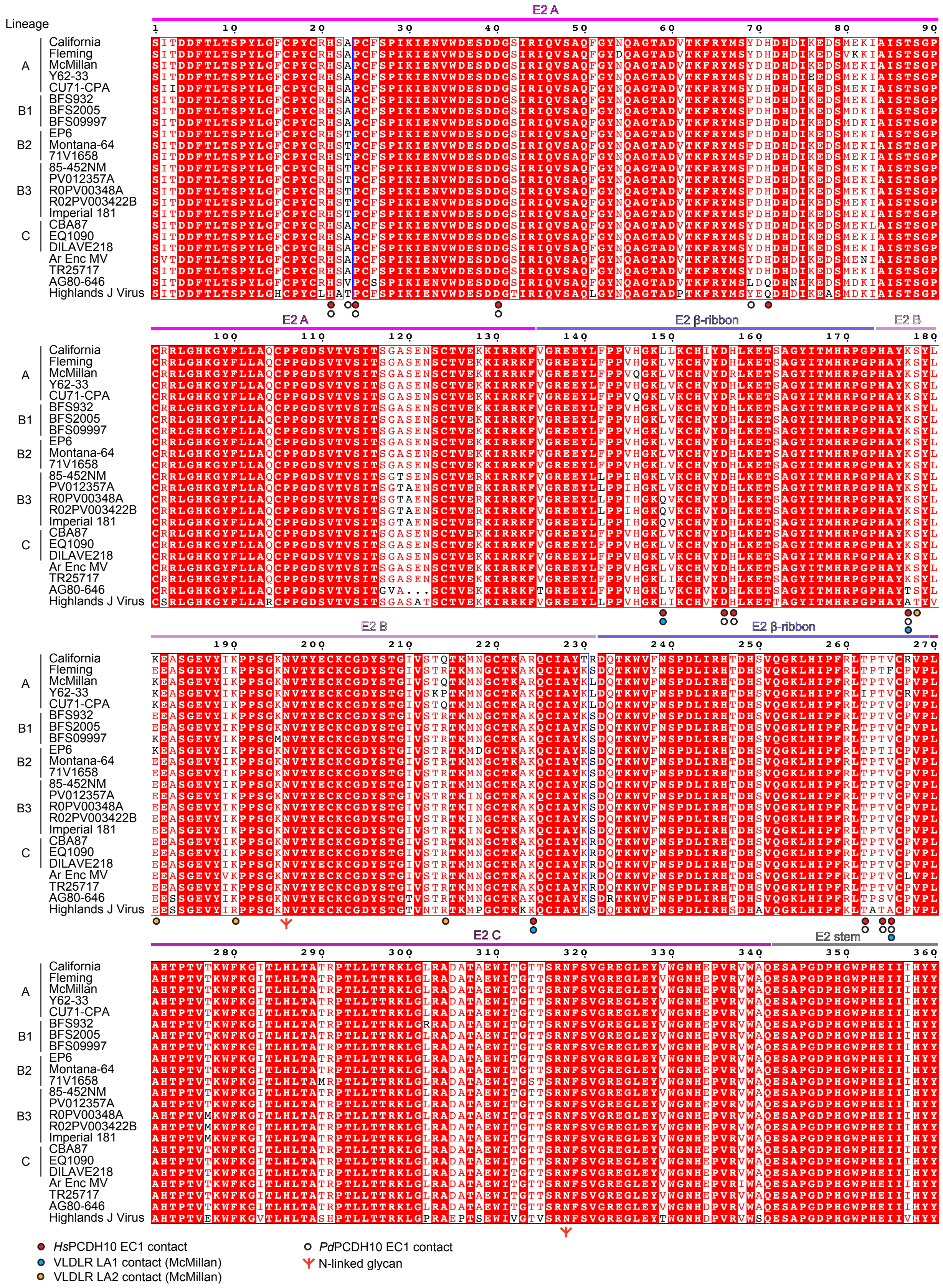

Lineage
A
B1
B2
B3
C
California
Fleming
McMillan
Y62-33
CU71-CPA
BFS932
BFS2005
BFS09997
EP6
Montana-64
71V1658
85-452NM
PV012357A
R0PV00348A
R02PV003422B
Imperial 181
CBA87
EQ1090
DILAVE218
Ar Enc MV
TR25717
AG80-646
Highlands J Virus
E2 A
E2 β-ribbon
E2 B
E2 C
E2 stem
HsPCDH10 EC1 contact
PdPCDH10 EC1 contact
VLDLR LA1 contact (McMillan)
N-linked glycan
VLDLR LA2 contact (McMillan)
