# Supplementary material for: Molecular basis for shifted receptor recognition by an encephalitic arbovirus
Source: Cell. Author manuscript; Available in PMC 2025 Sep 3. (PMC12406711; doi:10.1016/j.cell.2025.03.029)
Supplement: 13 — Table S1. Strain information for viral sequences used for RVP production or phylogenetic analyses, related to Figures 1, 6 and S1. Table S2. Cryo-EM data collection and validation statistics, related to Figures 1, 2 and 3. [file NIHMS2067620-supplement-13.pdf]

Table S1. Strain information for viral sequences used for RVP production or phylogenetic analyses, related to Figures 1, 6 and S1.

Virus	Strain	Host	Year	Location	Passage history	GenBank accession number
WEEV	California	Horse	1930	San Joaquin Valley, California, USA	p (?), sm (27), C6 (1)	KJ554965
	Ar Enc MV	Horse	1933	Monte Veloz, Buenos Aires, Argentina		KT844542
	Fleming	Human	1938	California, USA	sm (5), v (3)	MN477208
	McMillan	Human	1941	Ontario, Canada	mp (2), sm (2), v (2), C6 (1)	GQ287640
	BFS932	Mosquito	1946	Bakersfield, California, USA	sm (1), v (1)	KJ554966
	EP-6	Mosquito	1950	Missouri, USA	ce (1), C6 (1)	KJ554967
	BFS1703	Mosquito	1953	Bakersfield, California, USA	sm (1), C6 (1)	KJ554968
	BFS2005	Mosquito	1954	Bakersfield, California, USA	de (1)	GQ287644
	CBA87	Horse	1958	Oncativo, Córdoba, Argentina	sm (14), bhk (2), v (2)	KT844543
	TR25717	Horse	1959	Guyana		KT844541
	B11		1961	USA	p (?), v (5), bhk (2)	DQ432027
	E1416	White-crowned sparrow	1961	Kern County, California, USA	bhk (4), C6 (1)	KJ554969
	Y62-33	Mosquito	1962	Russia		KT844544
	Montana-64	Horse	1967	Montana, USA	de (1), C6 (1)	GQ287643
	S8-122	Grey squirrel	1968	Butte County, California, USA	sm (1), C6 (1)	KJ554970
	CU71-CPA		1971	Cuba		KT844545
	BFS3060	Mosquito	1971	Butte County, California, USA	ce (1), sm (1), C6 (1)	KJ554972
	71V1658	Horse	1971	Oregon, USA	v (2), smb (1)	GQ287645
	TBT235	Tortoise	1971	Texas, USA	wc (1), de (1), sm (1), bhk (1), C6 (1)	KJ554971
	75V9291	Mosquito	1975	Wilkin City, Minnesota, USA	v (2), C6 (1)	KJ554973
	R7973	Human	1975	Larimer County, Colorado, USA		OQ184867
	BFS09997	Mosquito	1978	Kern County, California, USA	v (1), C6 (1)	KJ554974
	AG80-646	Mosquito	1980	Chaco, Argentina	v (2), sm (1)	GQ287646
	Mn520		1981	Manitoba, Canada	p (?), v (2)	DQ393793
	Kern5547	Mosquito	1983	Kern County, California, USA	v (1), C6 (1)	KJ554975
	CHLV53	Mosquito	1983	Riverside County, California, USA	v (1), C6 (1)	KJ554976
	Mn548		1984	Manitoba, Canada	p (?), v (2)	DQ393794
	85-452NM	Mosquito	1985	New Mexico, USA	sm (2), C6 (1)	GQ287647
	CHLV31	Mosquito	1985	Riverside County, California, USA		KT844547

	PV02808A	Mosquito	1990	Lubbock County, Texas, USA	v (1) or sm (1), C6 (1)	KJ554977
	CO921356	Mosquito	1992	Larimer City, Colorado, USA	v (1), C6 (1)	KJ554979
	IMPR441	Mosquito	1992	Imperial County, California, USA	sm (1), C6 (1)	KJ554978
	93A30	Mosquito	1993	Phoenix, Arizona, USA	v (1), C6 (1)	KJ554982
	93A79	Mosquito	1993	Yuma, Arizona, USA	v (1), C6 (1)	KJ554983
	SUYA140	Mosquito	1993	Sutter County, California, USA		KT844548
	CNTR34	Mosquito	1993	Contra Costa County, California, USA	v (1), C6 (1)	KJ554984
	SAC 74	Mosquito	1993	Sacramento County, California, USA		KT844549
	Lake43	Mosquito	1994	Lake County, California, USA	v (2), C6 (1)	KJ554985
	Kern87	Mosquito	1996	Kern County, California, USA		KT844550
	97-5067	Turkey	1996	Madera County, California, USA		KU978771
	98-2345	Emu	1997	USA		KU978772
	PV72102	Mosquito	1997	El Paso County, Texas, USA	v (1) or sm (1), C6 (1)	KJ554986
	PV012357 A	Mosquito	2001	El Paso County, Texas, USA	v (1) or sm (1), C6 (1)	KJ554987
	R02PV002 957B	Mosquito	2002	El Paso County, Texas, USA	v (1) or sm (1), C6 (1)	KJ554988
	R02PV001 807A	Mosquito	2002	El Paso County, Texas, USA	v (1) or sm (1), C6 (1)	KJ554989
	Imperial 181	Mosquito	2005	Imperial County, California, USA	v (2)	GQ287641
	R02PV003 422B	Mosquito	2005	El Paso County, Texas, USA	v (1) or sm (1), C6 (1)	KJ554990
	R0PV0038 4A	Mosquito	2005	El Paso County, Texas, USA	v (1) or sm (1), C6 (1)	KJ554991
	DILAVE070	Horse	2023	Paysandú, Uruguay		PP620641
	DILAVE158	Horse	2023	San José, Uruguay		PP620642
	DILAVE198	Horse	2023	San José, Uruguay		PP620643
	DILAVE218	Horse	2023	Paysandú, Uruguay		PP620644
	EQ1090	Horse	2023	Rio Grande do Sul, Brazil	direct detection from brain samples	PP544260
	EQ1122	Horse	2023	Rio Grande do Sul, Brazil	direct detection from brain samples	PP669618
	DILAVE236	Horse	2024	Rocha, Uruguay		PP620645
	DILAVE255	Horse	2024	San José, Uruguay		PP620646
	EQ237	Horse	2024	Rio Grande do Sul, Brazil	direct detection from brain samples	PP669617
HJV	64A-1519	Horse	1964	Hillsborough County, Florida, USA	p (4), sm (1), v(1)	KT429021
	585-01	Red-tailed hawk	2001	Georgia, USA	v (1)	NC_012561
SFV	SFV4			Lab construct	Derived from molecular clone SFV3	AKC01668
EEEV	FL91-469	Mosquito	1991	Florida, USA	sm (1), v (3), bhk (2)	AY705241

MADV	267113	Human	2017	Darién, Panama		OR644805
SINV	360	Mosquito	2001	Queensland, Australia	C6 (1)	OL856089
CHIKV	37997	Mosquito	1983	Senegal	ap1, v2	AY726732
MAYV	BeH407	Human	1955	Brazil		MK573238
RRV	T48	Mosquito	1959	Australia	sm (6), C6 (1), v (1)	GQ433359
ONNV	UVRI0804	Human	2019	Uganda	v (1)	ON595759
GETV	AMM2021	Mosquito	1955	Malaysia	v (2), p (?)	MT121984
VEEV	TC83	Human	2017	Venezuela	Derived from VEEV strain TrD, passaged in the presence of SRI-34329	MZ399799

Passage numbers are in parentheses. "?" indicates unknown passage number. Abbreviations: mp: mouse; sm: suckling mouse; smb: suckling mouse brain; v: Vero cells; bhk: baby hamster kidney cells; wc: wet chicks; de: duck embryonic fibroblasts; ce: chick embryonic fibroblasts; C6: C6/36 (*Aedes albopictus*) cells; ehe: embryonated hen's eggs; gp: guinea pig; p: unknown media.

Table S2. Cryo-EM data collection and validation statistics, related to Figures 1, 2 and 3.

	WEEV CBA87 VLP: Human PCDH10$_{EC1}$-Fc		WEEV Imperial 181 VLP: Sparrow PCDH10$_{EC1}$-Fc	WEEV McMillan VLP: VLDLR$_{LBD}$-Fc		WEEV CBA87 VLP unliganded
				Dataset 1	Dataset 2	
Magnification	81,000		130,000	81,000	81,000	81,000
Voltage (kV)	300		300	300	300	300
Pixel Size (Å)	1.06		0.94	1.06	1.06	1.06
Electron dose (e/Å^2)	52.2		50	53.3	54.4	53.9
Defocus range (µm)	-0.6 to -1.8		-0.8 to -1.8	-0.8 to -1.8	0.8 to -1.8	-0.6 to -1.8
VLP reconstruction						
Initial particles	22,075		38,927	34,210	95,449	43,025
Final particles	13,141		22,304	18,464	54,308	31,430
Symmetry imposed	Icosahedral symmetry					
FSC threshold	0.143		0.143	0.143		0.143
Map resolution (Å)	4.8		4.8	5.9	4.8	5.4
Block-based reconstruction						
				Dataset 1	Dataset 2	
Initial Blocks	788,460		1,338,240	1,107,840	3,258,480	1,885,800
Final Blocks	343,889		401,369	1,720,156		443,570
Symmetry imposed	C1		C1	C1		C1
FSC threshold	0.143		0.143	0.143		0.143
	q3 block	E2–E1 trimer	E2–E1 trimer	q3 block	E2–E1 trimer	q3 block
Map resolution (Å)	3.3	2.9	2.8	2.9	2.8	3.4
**Model refinement and validation**						
Initial model used	AlphaFold2					
R.m.s deviations						
Bonds lengths (Å)	0.004		0.004	0.004		0.004
Bonds angles (º)	0.892		0.779	0.800		0.872
Validation						
Clashscore	8		6	7		9
Favored (%)	94		95	94		94
Allowed (%)	6		5	6		6
Disallowed (%)	0		0	0		0
