# Supplementary material for: Molecular basis for shifted receptor recognition by an encephalitic arbovirus
Source: Cell. Author manuscript; Available in PMC 2025 Sep 3. (PMC12406711; doi:10.1016/j.cell.2025.03.029)
Supplement: 2 — Figure S2. Binding experiments with PCDH10 EC1 and WEEV VLPs, related to Figures 1 and 2. (A) Coomassie-stained SDS-PAGE gel of purified VLPs. The experiment was performed twice, and representative gel images are shown. (B) Schematic diagrams of the PCDH10EC1-Fc, the PCDH10EC1 twin-strep tag, VLDLRLBD-Fc and VLDLR LA(1–2)-Fc constructs. (C) Size exclusion chromatography of Fc-fusion and twin-strep tagged proteins. Insets are SDS-PAGE gels of pooled peak fractions visualized using a stain-free imaging system. (D) Biolayer interferometry binding analysis of HsPCDH10EC1-twin-strep with immobilized WEEV CBA87 VLPs. (E) Scatchard plot for the biolayer interferometry binding analysis of HsPCDH10EC1-twin-strep with WEEV CBA87 VLPs shown in (D). The measured apparent affinity is indicated. (F) Representative ELISA results showing the binding of the HsPCDH10EC1-Fc or PdPCDH10EC1-Fc to WEEV CBA87, WEEV Imperial 181, or EEEV FL91–469 VLPs. Data shown are mean ± s.d. from one experiment performed with technical triplicates. The experiment was performed three times (n = 3) and representative data are shown. EC50 values shown are the mean of three independent experiments. [file NIHMS2067620-supplement-2.pdf]

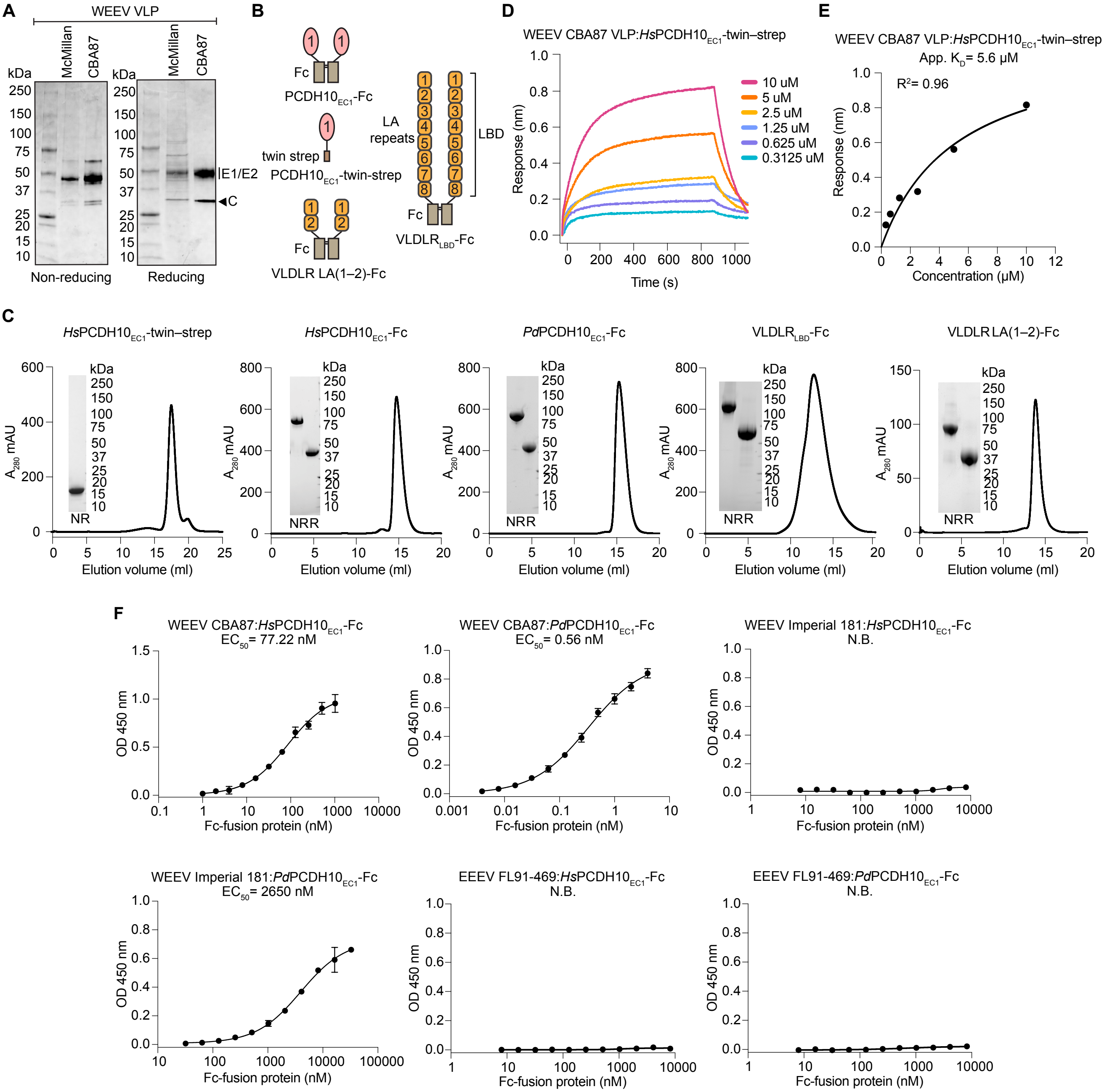

A
WEEV VLP
McMillan
CBA87
kDa
250
150
100
75
50
37
25
20
15
10
Non-reducing
Reducing
E1/E2
C
B
PCDH10EC1-Fc
twin strep
PCDH10EC1-twin-strep
VLDLR LA(1–2)-Fc
LA repeats
LBD
VLDLRLBD-Fc
Fc
D
WEEV CBA87 VLP:HsPCDH10EC1-twin–strep
10 uM
5 uM
2.5 uM
1.25 uM
0.625 uM
0.3125 uM
Response (nm)
Time (s)
E
WEEV CBA87 VLP:HsPCDH10EC1-twin–strep
App. KD= 5.6 µM
R2= 0.96
Response (nm)
Concentration (µM)
C
HsPCDH10EC1-twin–strep
HsPCDH10EC1-Fc
PdPCDH10EC1-Fc
VLDLRLBD-Fc
VLDLR LA(1–2)-Fc
A280 mAU
NR
NRR
Elution volume (ml)
F
WEEV CBA87:HsPCDH10EC1-Fc
EC50= 77.22 nM
WEEV CBA87:PdPCDH10EC1-Fc
EC50= 0.56 nM
WEEV Imperial 181:HsPCDH10EC1-Fc
N.B.
WEEV Imperial 181:PdPCDH10EC1-Fc
EC50= 2650 nM
EEEV FL91-469:HsPCDH10EC1-Fc
N.B.
EEEV FL91-469:PdPCDH10EC1-Fc
N.B.
OD 450 nm
Fc-fusion protein (nM)
